# Non-cell-autonomous cancer progression from chromosomal instability

**DOI:** 10.1038/s41586-023-06464-z

**Published:** 2023-08-23

**Authors:** Jun Li, Melissa J. Hubisz, Ethan M. Earlie, Mercedes A. Duran, Christy Hong, Austin A. Varela, Emanuele Lettera, Matthew Deyell, Bernardo Tavora, Jonathan J. Havel, Su M. Phyu, Amit Dipak Amin, Karolina Budre, Erina Kamiya, Julie-Ann Cavallo, Christopher Garris, Simon Powell, Jorge S. Reis-Filho, Hannah Wen, Sarah Bettigole, Atif J. Khan, Benjamin Izar, Eileen E. Parkes, Ashley M. Laughney, Samuel F. Bakhoum

**Affiliations:** 1 Human Oncology and Pathogenesis Program, Memorial Sloan Kettering Cancer Center, New York, NY USA; 2 Department of Radiation Oncology, Memorial Sloan Kettering Cancer Center, New York, NY USA; 3 Department of Physiology, Biophysics, and Systems Biology, Weill Cornell Medicine, New York, NY USA; 4 Meyer Cancer Center, Weill Cornell Medicine, New York, NY USA; 5 Institute for Computational Biomedicine, Weill Cornell Medicine, New York, NY USA; 6 Bioinformatics Facility, Institute of Biotechnology, Cornell University, Ithaca, NY USA; 7 Volastra Therapeutics Inc., New York, NY USA; 8 Department of Oncology, Medical Sciences Division, University of Oxford, Oxford, UK; 9 Columbia Center for Translational Immunology, New York, NY USA; 10 Division of Hematology and Oncology, Columbia University Medical Center, New York, NY USA; 11 Department of Pathology, Harvard Medical School, Boston, MA USA; 12 Center for Systems Biology, Massachusetts General Hospital, Boston, MA USA; 13 Department of Pathology and Laboratory Medicine, Memorial Sloan Kettering Cancer Center, New York, NY USA

**Keywords:** Chronic inflammation, Chromosomes, Cancer microenvironment, Metastasis, Tumour immunology

## Abstract

Chromosomal instability (CIN) is a driver of cancer metastasis^1–4^, yet the extent to which this effect depends on the immune system remains unknown. Using ContactTracing—a newly developed, validated and benchmarked tool to infer the nature and conditional dependence of cell–cell interactions from single-cell transcriptomic data—we show that CIN-induced chronic activation of the cGAS–STING pathway promotes downstream signal re-wiring in cancer cells, leading to a pro-metastatic tumour microenvironment. This re-wiring is manifested by type I interferon tachyphylaxis selectively downstream of STING and a corresponding increase in cancer cell-derived endoplasmic reticulum (ER) stress response. Reversal of CIN, depletion of cancer cell STING or inhibition of ER stress response signalling abrogates CIN-dependent effects on the tumour microenvironment and suppresses metastasis in immune competent, but not severely immune compromised, settings. Treatment with STING inhibitors reduces CIN-driven metastasis in melanoma, breast and colorectal cancers in a manner dependent on tumour cell-intrinsic STING. Finally, we show that CIN and pervasive cGAS activation in micronuclei are associated with ER stress signalling, immune suppression and metastasis in human triple-negative breast cancer, highlighting a viable strategy to identify and therapeutically intervene in tumours spurred by CIN-induced inflammation.

## Abstract (web summary)

Chromosomal instability in cancer is linked to endoplasmic reticulum stress signalling, immune suppression and metastasis, which is mediated by the cGAS–STING pathway, suppression of which can reduce metastasis.

## Main

Chromosomal instability (CIN) is a cancer hallmark^5^ that is associated with therapeutic resistance^6^, immune evasion^7,8^ and metastasis^2^. CIN arises from ongoing errors in chromosome segregation during mitosis^9,10^. In normal cells, chromosome missegregation is poorly tolerated^11^ and can suppress oncogenic transformation^12,13^. Yet, advanced human cancers are often characterized by elevated rates of chromosome missegregation and aneuploidy^2,14,15^, invoking adaptive processes that allow tumours to withstand and co-opt CIN^3^. Using isogenic models that enable genetic manipulation of chromosome missegregation rates in cancer cells^16^, we have previously shown that CIN promotes metastasis by inducing a cytosolic double-stranded DNA (dsDNA) response in tumour cells, mediated by the cGAS–STING innate immune pathway^2^. Errors in chromosome segregation lead to the formation of rupture-prone micronuclei^17^ and exposure of genomic dsDNA to the cytoplasm^2,18,19^. These findings were based on partially immune compromised tumour models^2^; thus, it remained unknown whether the effect of CIN on tumour progression is cancer cell autonomous or rather dependent on the immune system. Moreover, it is unclear how chromosomally unstable tumours adapt to CIN and evade immune surveillance that would arise from cGAS–STING activation and a downstream type I interferon (IFN) response^20^.

## Immune dependence of CIN-driven metastasis

**Extended Data Fig. 1:**
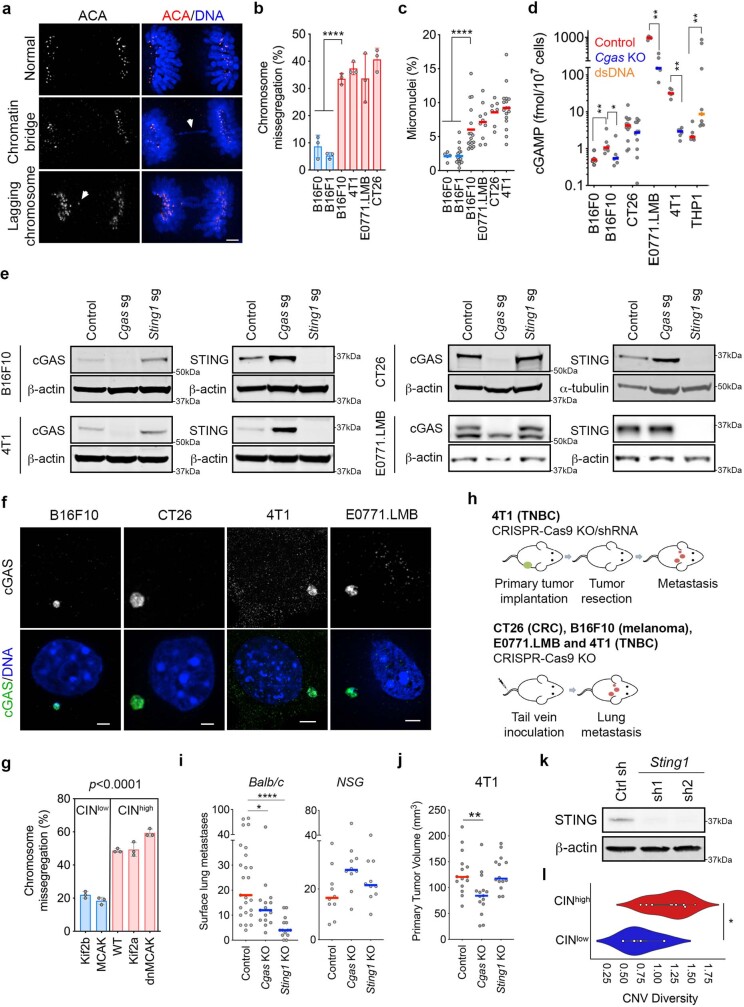
CIN-induced cGAS–STING activation drives metastasis in immunocompetent models. **a**, Representative images of 4T1 TNBC cells undergoing anaphase with various chromosome segregation defects stained using DAPI (DNA) and anti-centromere antibody (ACA), scale bar 5 μm. **b**, Frequency of anaphase cells with chromosome segregation defects in poorly metastatic (B16F0 and B16F1) and highly metastatic (B16F10, 4T1, EO771.LMB and CT26) cells; bars represent average ± SD, **** *p* < 0.0001, two-sided t-test, n = 3 independent counting per cell line, ~ 150 division events per counting. **c**, Percentage of micronuclei in the various cell lines; bars represent median, **** *p* < 0.0001, two-sided Mann-Whitney test, n = 5–17. **d**, cGAMP levels in cell lysates; bars represent median values, * *p* < 0.05, ** *p* < 0.01, two-sided Mann-Whitney test, n = 5–12. **e**, Immunoblots for cGAS and STING of control, *Cgas*-KO, and *Sting1*-KO B16F10, 4T1, and CT26 cells with β-Actin as a loading control. **f**, Representative images of B16F10, CT26, 4T1 and EO771.LMB cells with micronuclei stained using DAPI (DNA) and anti-cGAS antibody, scale bar 5 μm. **g**, Percentage of 4T1 cells undergoing anaphase with evidence for chromosome missegregation, bars represent mean ± SD, n = 150 cells in 3 biological replicates, **** *p* < 0.0001, two-sided t-test. **h**, Experimental schema for metastasis experiments. **i**, The number of surface lung metastasis metastases arising after 4T1 tumor resection in BALB/c hosts (n = 14–25) or arising from tail-vein injection of 4T1 cells into NSG hosts (n = 10); lines in the plot represent the median, * p < 0.05, **** p < 0.0001, two-sided Mann-Whitney test. **j**, Volume of resected orthotopically transplanted primary 4T1 tumors; bars represent median values, ** p < 0.01, two-sided Mann-Whitney test, n = 14–15 animals per group. **k**, Immunoblots of cGAS and STING of control and *Sting1*-depleted 4T1 cells with β-actin as a loading control. **l**, Violin plot showing the distribution of tumor cell CNV diversity (Methods) in CIN^low^ (n = 4) and CIN^high^ (n = 9) murine tumor samples (one-sided t-test p-value <0.05). Overlaid box plots denote the minima, maxima, median, and 1^st^ and 3^rd^ quartiles. Source Data

To interrogate the influence of the immune system on CIN-driven metastasis, we used four syngeneic metastatic cancer models, including triple-negative breast cancer (TNBC) (4T1 and EO771.LMB), colorectal adenocarcinoma (CT26) and melanoma (B16F10). All models exhibited elevated rates of chromosome segregation errors during anaphase and a preponderance of micronuclei (Extended Data Fig. 1a–c). Highly metastatic melanoma cells (B16F10) had significantly higher rates of CIN compared with their less metastatic parental counterparts (B16F0 and B16F1, Extended Data Fig. 1b,c). In all models, we observed CIN-dependent activation of cGAS–STING, as evidenced by cGAS localization in micronuclei, measurable cGAMP levels from cell lysates in a manner dependent on cGAS expression and detectable STING protein levels (Extended Data Fig. 1d–f). We also manipulated CIN levels in 4T1 cells through expression of the non-motile kinesin-13 proteins, Kif2b or MCAK^16^, either of which led to significant reductions in anaphase chromosome missegregation compared with wild-type (WT) cells, or cells expressing a dominant-negative MCAK mutant (dnMCAK)^21^ (Extended Data Fig. 1g). Expression of Kif2a, a kinesin-13 family member that possesses microtubule depolymerizing activity but lacks a centromere or kinetochore targeting domain, had no impact on CIN (Extended Data Fig. 1g).

**Fig. 1:**
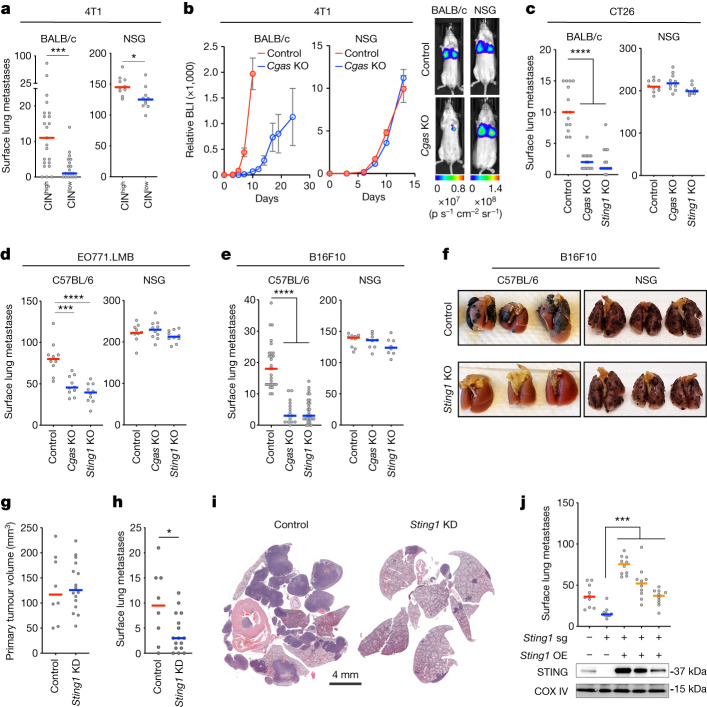
CIN drives cancer progression through tumour cell non-autonomous mechanisms. **a**, Number of surface lung metastases arising from orthotopically transplanted and resected CIN^high^ or CIN^low^ 4T1 tumours in BALB/c hosts (*n* = 19 and 23 animals for CIN^low^ and CIN^high^, respectively) or from tail-vein-injected CIN^high^ or CIN^low^ 4T1 cells in NSG hosts (*n* = 10); bars represent the median; ***P < 0.001, *P < 0.05, two-sided Mann–Whitney test. **b**, Normalized bioluminescence (BLI) signal from BALB/c or NSG mice tail-vein injected with 4T1 control and *Cgas*-KO cells (*n* = 10 animals per condition) and representative bioluminescence images on days 5 and 8 for BALB/c and NSG mice, respectively; mean ± s.e.m. **c**–**e**, Number of surface lung metastases upon tail-vein injection of control, *Cgas-*KO or *Sting1*-KO CT26 (**c**), EO771.LMB (**d**) or B16F10 cells (**e**) into immune competent hosts (BALB/c for CT26, C57BL/6 for EO771.LMB and B16F10) or NSG hosts; ****P < 0.0001, ***P < 0.001, two-sided-Mann–Whitney test; *n* = 8–29 mice per group. **f**, Representative lung images from C57BL/6 or NSG animals tail-vein-injected with control or *Sting1*-KO B16F10 cells. **g**, Volume of resected orthotopically transplanted control and *Sting1*-depleted primary 4T1 tumours; *n* = 8–16 mice per condition. **h**, Number of surface lung metastases in animals arising after tumour resection; lines in the plot represent the median; *P < 0.05, two-sided *t*-test after testing for normality. **i**, Representative haematoxylin and eosin (H&E)-stained lungs 3 weeks after resection of control or *Sting1*-depleted orthotopically transplanted 4T1 tumours. **j**, Number of surface lung metastases arising from tail-vein injection of 4T1 control, *Sting*1-KO and *Sting*1-KO cells with exogenous overexpression (OE) of STING and immunoblot for STING and CoxIV of the cells; lines in the plot represent the median; ***P < 0.001, two-sided Mann–Whitney test. KD, knockdown; p s^−1^ cm^−2^ sr^−1^, photon second^–1^ centimeter^–2^ steradian^–1^; sg, single guide. Source Data

We next transplanted CIN^high^ (WT, Kif2a or dnMCAK expressing) and CIN^low^ (Kif2b or MCAK expressing) 4T1 tumours in immune competent (BALB/c) and severely immune compromised (NOD-scid IL2Rγ^null^, thereafter referred to as NSG) mice. There was an 11-fold difference in the median number of surface lung metastases in the BALB/c mice when comparing CIN^high^ and CIN^low^ tumours as opposed to only a 1.1-fold difference in NSG hosts (Fig. 1a). We then depleted *Cgas* or *Sting1* from CIN^high^ 4T1, B16F10, EO771.LMB and CT26 cells using CRISPR–Cas9 knockout (KO) (Extended Data Fig. 1e). Tail-vein inoculation or orthotopic transplantation of WT, *Cgas*-KO or *Sting1*-KO cells in BALB/c (4T1 and CT26) or C57BL/6 (B16F10 and EO771.LMB) led to a significant reduction in lung colonization and metastasis as assessed directly through enumeration of surface lung metastases or using bioluminescence imaging (Fig. 1b–f and Extended Data Fig. 1h,i). Strikingly, this phenotype was entirely dependent on the immune system, as transplantation of these cells in NSG hosts completely abolished the effect of *Cgas* or *Sting1* KO on metastasis (Fig. 1b–f and Extended Data Fig. 1h,i). Loss of cancer cell *Sting1* did not impact primary tumour size, whereas *Cgas*-KO tumours were slightly smaller compared with control tumours, as previously reported^22^ (Extended Data Fig. 1j). To rule out potential off-target effects from CRISPR–Cas9-mediated KO, we depleted *Sting1* using short hairpin RNA (shRNA) and observed a similar reduction in lung metastasis with no impact on primary tumour formation (Fig. 1g–i and Extended Data Fig. 1k). Furthermore, complementation of *Sting1*-KO cells with constructs expressing WT *Sting1* using different promoters revealed a dose-dependent relationship between *Sting1* re-expression and metastasis (Fig. 1j).

## CIN and STING promote immune suppression

**Fig. 2:**
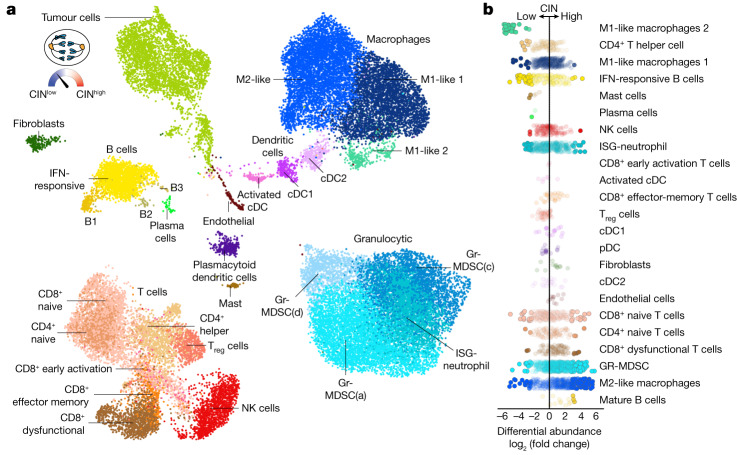
CIN-induced STING signalling engenders an immune-suppressive tumour microenvironment. **a**, Uniform manifold approximation and projection (UMAP) of all single cells coloured by cell subtype assignment; includes carcinoma, as well as immune and other stromal cell types within the TME (*n* = 39,234 cells). Macro cell-type assignments are capitalized. Inset, schematic showing that tumour cell rates of CIN were genetically dialled-up or dialled-down. **b**, Strip plot showing CIN-dependent effects on differential abundance, log_2_(fold change (FC)), at the neighbourhood level grouped by cell subtype and ranked by mean log_2_(FC) within each cell subtype. Node opacity is scaled by *P* value, such that more significant neighbourhoods are more opaque and *P* ≤ 0.1 neighbourhoods are completely opaque. cDC, classical dendritic cells; pDC, plasmacytoid dendritic cells; T_reg_, regulatory T cells.

**Extended Data Fig. 2:**
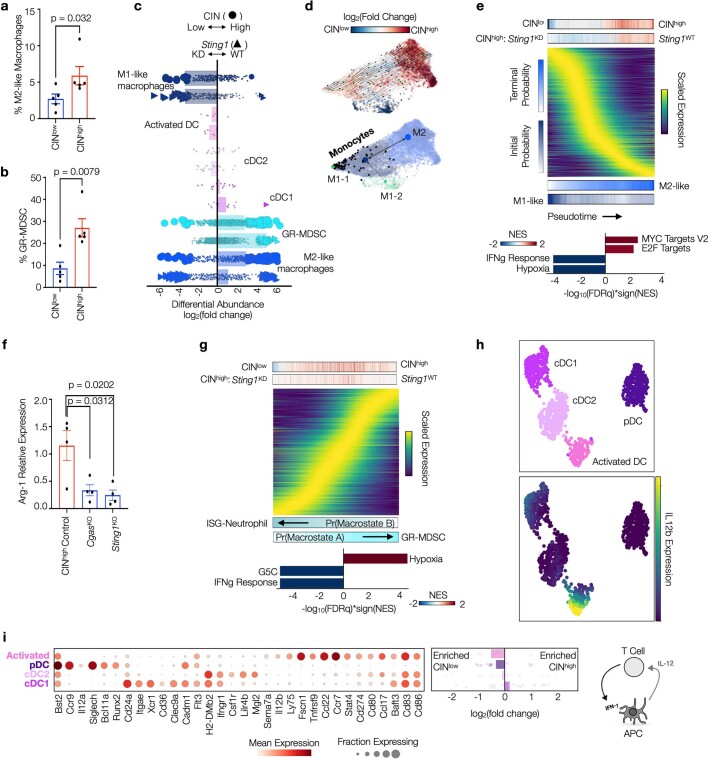
CIN and STING dependent effects on the tumor myeloid landscape. **a**, Percentage of M2 macrophage among total infiltrating CD45+ cells in CIN^low^ and CIN^high^ 4T1 primary tumors resected on day 14; bars represent mean ± s.e.m., two-sided Mann-Whitney test, n = 5 independent biological replicates. **b**, Percentage of GR-MDSC among total infiltrating CD45+ cells in CIN^low^ and CIN^high^ 4T1 primary tumors resected on day 14; bars represent mean ± s.e.m., two-sided Mann-Whitney test, n = 5 independent biological replicates. **c**, Myeloid population strip plot showing conserved CIN- and STING-dependent differential abundance effects (mean enrichment must be both positive or both negative) at the neighborhood level grouped by cell subtype and ranked by mean CIN-dependent log_2_(fold change) of neighborhoods within each cell subtype. Node size is scaled by p-value, so that more significant differential abundance neighborhoods are larger. Bar plots show the mean log_2_(fold change) of neighborhoods with significant scores (p-value <= 0.1); if fewer than two significant neighborhoods are detected, all neighborhoods are used in computing the mean. **d**, Force-directed layout with transition vectors of all macrophages (n = 9,800 cells) colored by CIN-dependent differential abundance computed in local neighborhoods using Milo and mapped to single cells for visualization (Supplementary Information, *top*) and cell subtype (*bottom*). The overlayed directed partitioned-based graph abstraction (PAGA) shows the inferred transitions between subtype clusters based on Palantir pseudotime with nodes scaled by relative subtype size and arrows scaled by transition confidence. The overlaid black nodes show cells with Monocyte probability >=95%, computed by CellAssign; the green node highlights the initial seed cell for Palantir pseudotime. **e**, Scaled imputed expression of transition genes (Supplementary Information) for all macrophages (n = 9,800 cells) ranked along pseudotime. For each gene, expression was modeled using a generalized additive model (GAM) along the M2-like macrophage lineage. Ranked color bars above heatmap show CIN- and STING-dependent differential abundance, log_2_(fold change), computed in local neighborhoods using Milo and mapped to single cells for visualization (Supplementary Information). Additional ranked color bars below the heatmap show CellRank terminal state probability and initial state probability along macrophage pseudotime. The bar plot shows top two most enriched gene signatures enriched along macrophage pseudotime (FDR < 0.005). The x-axis shows the −log_10_(FDR q-value) times the sign of the pathway normalized enrichment score (NES) and color indicates the pathway NES. Complete list of genes and gene set enrichment analysis (GSEA) results for cells ranked along macrophage pseudotime, including nominal and corrected p-values, are provided in Supplementary Table 8. **f**, Relative expression of Arg-1 in macrophages cultured for 24 h with conditioned medium from 4T1 tumor cells; bars represent mean ± s.e.m., two-sided t-test, n = 4 independent biological replicates. **g**, Scaled imputed expression of transition genes (Supplementary Information) for all ISG-Neutrophils and GR-MDSCs (n = 12,593 cells) ranked along CellRank ISG-Neutrophil macrostate probability. For each gene, expression was modeled using a generalized additive model (GAM) as in **(e)**. Ranked color bars above heatmap show CIN- and STING-dependent differential abundance, log_2_(fold change), computed in local neighborhoods using Milo and mapped to single cells for visualization (Supplementary Information). Additional ranked color bars below the heatmap show CellRank ISG-Neutrophil macrostate and GR-MDSC(a) macrostate probabilities ranked by ISG-Neutrophil macrostate probability. The bar plot shows top gene signatures enriched along ISG-Neutrophil macrostate probability with FDR < 0.05 and abs(NES) > 2.5. The x-axis shows the −log_10_(FDR q-value) times the sign of the pathway normalized enrichment score (NES) and color indicates the pathway NES. Complete list of genes and gene set enrichment analysis (GSEA) results for cells ranked along macrophage pseudotime, including nominal and corrected p-values, are provided in Supplementary Table 8. **h**, UMAP projection for the dendritic cell subset (n = 1,075 cells) colored by DC subtype (*top*) and imputed IL12b expression (*bottom*). **i**, *Left*, dot plot showing relative frequency of dendritic cells expressing canonical lineage markers (any counts detected) and the average log-transformed expression of each gene per dendritic cell subtype. Genes are clustered using the average cosine distance and subtypes are ordered according to (*middle*) average CIN-dependent differential abundance of local neighborhoods mapped to dendritic cell subtypes (Supplementary Information). Complete DEG and GSEA results per dendritic cell subtype (relative to all other dendritic cells), including nominal and corrected p-values, are provided in Supplementary Table 8. *Right*, Example of IFN-promoting feedback loop between antigen presenting cells (APCs) and T cells. Source Data

**Extended Data Fig. 3:**
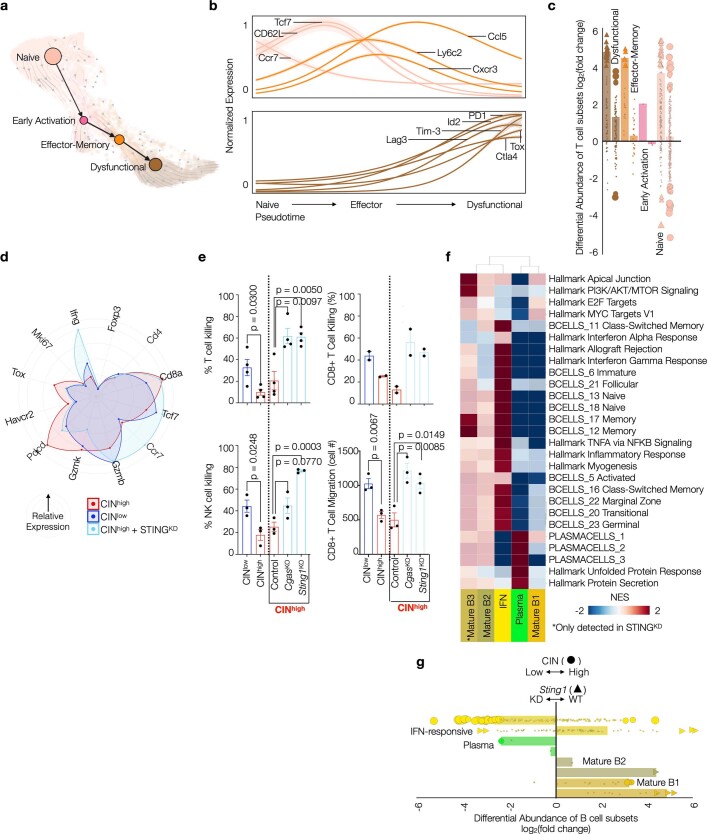
CIN and STING dependent effects on the tumor lymphoid landscape. **a**, Force-directed layout with transition vectors of all CD8+ T cells color by subtype (n = 4,797 cells). The overlaid, directed PAGA shows inferred transition between subtype clusters based on Palantir pseudotime with nodes scaled by subtype size and arrows scaled by transition confidence. **b**, Activated (*top*) and dysfunctional (*bottom*) gene trends along CD8+ T cell pseudotime using scaled imputed expression modeled by a GAM. Complete list of correlation-ranked genes and GSEA results along CD8+ T cell pseudotime, including nominal and corrected p-values, are provided in Supplementary Table 8. **c**, Strip plot showing CIN- (circular nodes) and STING- (triangle nodes) dependent differential abundance at the neighborhood level grouped by cell subtype. Node size is scaled by p-value, so that more significant differential abundance neighborhoods are larger. Bar plots show the mean log_2_(fold change) of neighborhoods with significant (p-value <= 0.1) differential abundance scores; if fewer than two significant neighborhoods detected, all neighborhoods are used in computing the mean. **d**, Radar plot showing relative expression of key marker genes per condition in CD8+ T cells. Gene expression is normalized by 1 or the max average marker expression across all conditions, whichever is higher. **e**, Percentage of 4T1 cells killed after co-cultured with T cells, CD8+ T cells, and natural killer cells, number of immune cells migrating from the upper compartment to the bottom compartment where 4T1 tumor cells seeded; bars represent mean ± s.e.m., two-sided t-test, n = 4 (T cells), 3 (NK cells), 2 (CD8+, no error bar), or 3 (migration). **f**, Clustered heatmap showing the normalized enrichment score (NES) of relevant gene signatures differentially expressed within B cell subtypes (relative to all other B cells) with an FDR q-value < 0.05 in at least one subtype. Signatures not meeting the FDR q-value threshold are opaque. Complete DEG and GSEA results per B cell subtype (relative to all other B cells), including nominal and corrected p-values, are provided in Supplementary Table 8. **g**, Same as **(c)**, but for B cell subtypes and ordered by mean CIN-dependent log_2_(fold change).

We orthotopically transplanted CIN^high^, CIN^low^ and *Sting1*-depleted CIN^high^ 4T1 cells in the mammary fat pad of BALB/c mice and performed single-cell RNA sequencing (scRNA-seq) of freshly resected 14-day-old tumours (Fig. 2a). As expected, CIN^high^ tumour cells exhibited significantly higher karyotype diversity as inferred from scRNA-seq data compared with their CIN^low^ counterparts (Extended Data Fig. 1l). At a high level, CIN engendered a pro-metastatic tumour microenvironment (TME) that was markedly enriched in immune-suppressive macrophages, granulocytic myeloid-derived suppressor cells (Gr-MDSCs) and dysfunctional T cells (Fig. 2b and Extended Data Figs. 2a–g and 3a–d). Conversely, CIN^low^ tumours were enriched in pro-inflammatory macrophages, IFN-responsive B cells, activated dendritic cells and CD4^+^ T helper cells (Fig. 2b and Extended Data Figs. 2c,h,I and 3). Importantly, depleting cancer cell *Sting1* in CIN^high^ tumours abolished many of the effects of CIN on the TME, ultimately restoring it to a CIN^low^-like state (Extended Data Figs. 2c,e–g and 3). Some of the scRNA-seq findings were validated through flow cytometry, revealing enrichment of CD11b^+^ and CD206^+^ as well as CD11b^+^Ly6G^+^ cells in CIN^high^ compared with CIN^low^ tumours (Extended Data Fig. 2a,b). Coculture of CIN^high^ tumour cells with macrophages led to significant reduction in relative arginase expression upon loss of cancer cell *Cgas* or *Sting1* (Extended Data Fig. 2f). And suppression of CIN or knockout of either *Cgas* or *Sting1* in CIN^high^ cells enhanced CD8^+^ T cell migration and led to increased tumour cell killing by pan-T cells, CD8^+^ T cells or natural killer (NK) cells (Extended Data Fig. 3e).

## ContactTracing to map cell–cell interactions

**Fig. 3:**
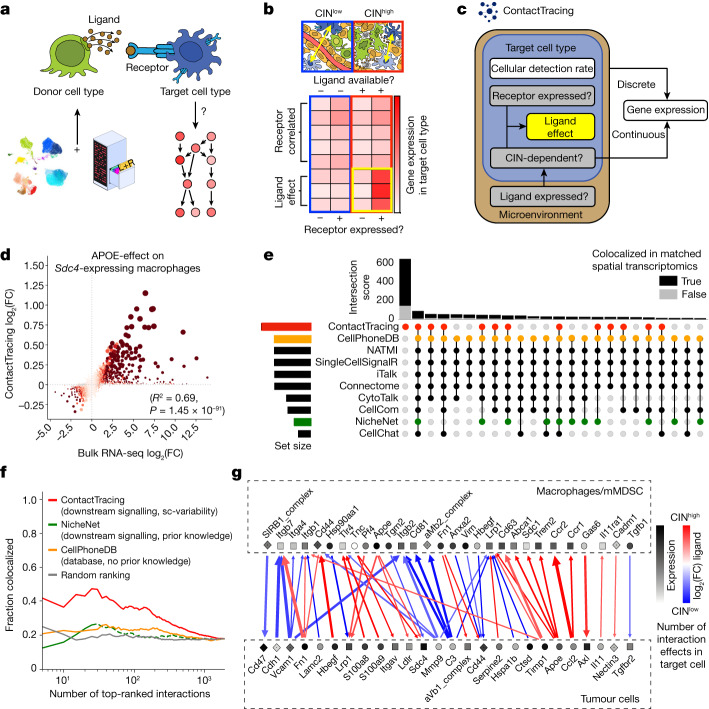
ContactTracing infers conditionally dependent ligand effects in vivo from single-cell variability. **a**, ContactTracing infers the effect of ligand–receptor-mediated interactions on target (receptor-expressing) cells. (**b**) Inferences are based on intrinsic biological variability in receptor expression on target cells and ligand abundance in the TME; we focus on CIN- and STING-dependent ligand effects. **c**, Plate diagram of the ContactTracing Hurdle model. Plates represent the conditional dependence of variables within the TME and within a target cell population. Hurdle models are fitted using MAST, which splits models into discrete and continuous components. White boxes depict variables predicted by MAST, and grey boxes indicate variables that ContactTracing calculates to identify CIN-dependent ligand effects (yellow box). **d**, Correlation between APOE effect on macrophages inferred from single-cell variability (ContactTracing) and defined through bulk RNA-seq comparison of ligand-treated versus untreated macrophages. Each node represents a gene; log(FC) expression in bulk RNA-seq (*x* axis) as compared with that inferred from scRNA-seq (*y* axis) for APOE receptor, Sdc4. Node size is proportional to −log_10_(FDR) of scRNA-seq target test, and node colour is proportional to −log_10_(FDR) of bulk RNA-seq test for differential expression. *R*^2^ is Pearson’s correlation coefficient; *P* value is two-sided and testing for correlation. **e**, UpSet plot showing intersection between top 1,000 interactions (each defined by a unique receptor and target cell type) predicted by ContactTracing and other cell–cell interaction methods in human TNBC samples, for which there exist matched single-cell and spatial transcriptomics data. Histogram shows fraction of significantly colocalized interactions in a 200-μm radius on matched spatial transcriptomics data (Methods) for each set. **f**, Colocalization of non-secreted interactions within a 50-μm visium spot, reported as a function of number of top-ranked interactions. Each interaction is defined by [ligand, receptor, target cell type], and is designated as colocalized by a nominal one-sided permutation-based *P* < 0.05; fraction colocalized was assessed for ContactTracing (considers downstream signalling, no prior knowledge), CellPhoneDB (no downstream signalling), NicheNet (prioritizes interactions exhibiting downstream signalling based on prior knowledge) and for randomly ranked interactions as a function of number of top interactions. Lines represent the average fraction of colocalized interactions across four patient tumours with matched spatial transcriptomics data. Dotted lines represent interactions that did not pass prefiltering steps of NicheNet or CellPhoneDB; these interactions were sorted randomly and assigned the lowest score. **g**, CIN- and STING-dependent interactions between tumour cells and macrophages, predicted by ContactTracing. Significant interactions are defined by receptor-expressing target cells that exhibit at least 10 significant interaction effects (FDR < 0.25) when the cognate ligand is conditionally available in the TME, ligand abs(log_2_(FC)) > 0.12 at FDR < 0.05, with log_2_(FC) having the same sign for both the CIN and STING comparisons. abs, absolute; mMDSC, myeloid-derived suppressor cell; sc-variability, single cell-variability.

**Extended Data Fig. 4:**
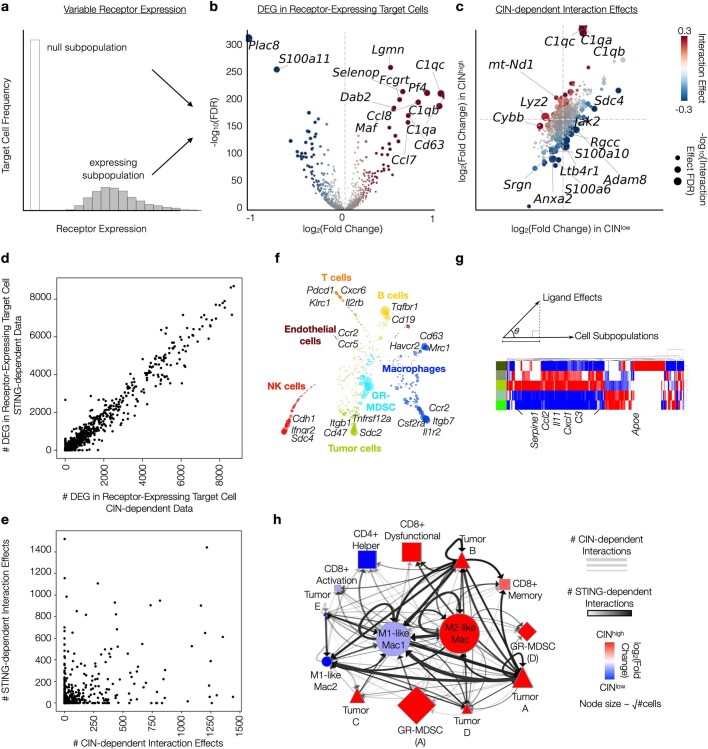
*ContactTracing* inference of cell-cell interactions from single cell data. **a**, Illustrative histogram showing log-transformed expression of the Mrc1 receptor in Macrophages (target cell type) for the receptor-null (white) and receptor-expressing (gray) subsets. **b**, Volcano plot showing genes differentially expressed in Mrc1-expressing vs. Mrc1-null macrophages (target cell type). Nodes are scaled by the absolute value of the transcriptional response score; top up- and down-regulated genes are labeled. **c**, Visual summary of MAST results from the CIN-dependent interaction test (see Methods); shown here for Mrc1 receptor expression in macrophage target cells. Each node represents a highly variable gene. The x-axis shows the log2-fold change estimate in receptor-expressing vs. receptor-null target cells in the CIN^low^ condition. The y-axis shows the same parameter estimate, but computed in the CIN^high^ condition. Node size is proportional to the significance of the interaction effect, and the node color represents the magnitude of the interaction effect, which here shows CIN-dependent amplification of the transcriptional response. **d**, Each node represents a receptor/cell type combination, on the x-axis is the number of genes with significant transcriptional response (FDR < 0.05) in the CIN^high^/CIN^low^ dataset; on the y-axis is the same value for the CIN^high^ *Sting1*^WT^/*Sting1*^KD^ data set. **e**, As in **d**, except the number of genes with significant conditionally-dependent interaction effect (FDR < 0.05) is shown. **f**, UMAP projection based on STING-dependent interaction effects in CIN^high^ tumors. The effect matrix has a row for each receptor/cell-type combination with at least one significant interaction effect (FDR q-value < 0.05), and a column for every gene. Each entry in the matrix is −log_10_(p-value)*interaction_coef. Node color reflects the cell type in which the ligand effect is measured and node size reflects the number of significant condition-specific interaction effects in target cells expressing the receptor. **g**, Transcriptional response states are mapped to individual cell clusters by taking the dot-product between the transcriptional response score for a given gene (given by x-axis) and its log2(expression fold change), here shown for one tumor subcluster vs. all other tumor cells (y-axis) and visualized using a clustered heatmap based on the average Euclidean distance metric. Red: positive dot-product, blue: negative dot-product, white: any value with abs(dot product) < 0.5. The log2(expression fold change) was set to zero if it was not significant (FDR q-value > 0.15) prior to computing the dot product. **h**, *ContactTracing* network plot corresponding to data in Fig. 4a. Here, nodes represent cell subtypes; node size is scaled by their relative fraction in the TME, and color reflects their average CIN-dependent differential abundance. Directed arrows represent interactions between cell subtypes (emanating from ligand-producing, donor cell subtype to receptor-expressing, target cell subtype), with arrow thickness encoding the total number of CIN-dependent interactions predicted between each pair of subtypes, and arrow darkness reflecting the number of STING-dependent interactions.

To determine how CIN-induced STING signalling reprograms the TME, we developed a fundamentally new, systems-level approach to predict the effect of conditionally dependent cell–cell interactions in the TME, called ContactTracing. Our strategy exploited intrinsic variability in scRNA-seq data to infer cellular responses to ligand–receptor-mediated interactions. Importantly, this was done without relying on prior knowledge of downstream target genes, allowing unbiased discovery of heretofore unknown cellular responses to receptor engagement. This method was based on the simple premise that, within a given tumour, it is unlikely that all donor (ligand-producing) cells and target (receptor-expressing) cells are fully engaged in a particular cell–cell interaction. Exploiting inherent biological variability in (1) receptor expression on target cells and (2) sample-level ligand availability in the TME, we predicted the effect of a ligand on its target cell in its native, in vivo context (Fig. 3a–c and Methods). For all putative ligand–receptor-mediated interactions, we performed a likelihood ratio test between receptor-expressing and receptor-null target cells (Extended Data Fig. 4a,b), which could capture unwanted confounding (correlation) between receptor expression and the expression of other genes. However, by exploiting secondary variability in ligand availability across experimental conditions—such as levels of CIN or cancer cell STING expression (Extended Data Fig. 4c)—we distinguished ligand effects from genes merely co-expressed with the relevant receptor (Fig. 3b,c and Extended Data Fig. 4c). True ligand effects were not correlated across conditions, unlike their unobserved confounders (Extended Data Fig. 4d,e). Ligand effects (that is, distinct transcriptional responses in receptor-expressing target cells when the ligand is present) largely clustered by cell type (Extended Data Fig. 4f), and were mapped back to subpopulations within the target cell type (Extended Data Fig. 4g).

**Extended Data Fig. 5:**
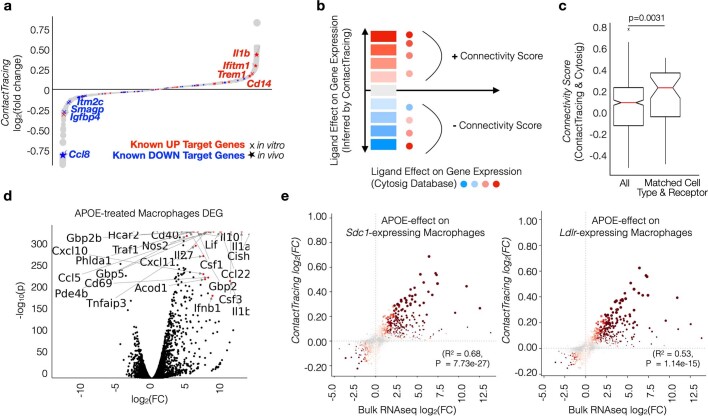
*ContactTracing* validation. **a**, *ContactTracing*-predicted ligand effect in CCR2-expressing macrophages ranked and scaled by observed log_2_(fold change). Known target genes are annotated *in vivo* if defined in, or *in vitro* if within the top or bottom 20 responses reported in the CytoSig databases (ranked based on log_2_(fold change) of cytokine-treated cell lines in culture). **b**, Illustration of connectivity score used to compare ligand effects inferred by *ContactTracing* to the those reported in the CytoSig database (tests whether up-regulated genes in one list are also up-regulated in another, Methods). **c**, Distribution of connectivity scores between all CIN-dependent interaction effects predicted by *ContactTracing* compared to gene responses reported by CytoSig for ligands matching the same set of receptors in cell types relevant to the breast cancer TME (Methods); the left box shows the distribution of connectivity scores across all comparisons (n = 558). The right box shows enrichment of connectivity scores for comparisons with matched receptors in similar macro cell types (n = 44, two-sided Mann-Whitney test, p = 0.003). The boxes span the 1^st^–3^rd^ quartiles, with red line indicating the median, whereas whiskers denote the rest of distribution within 1.5x the interquartile range, other outliers indicated with an x **d**, Volcano plot showing differentially expressed genes induced by APOE treatment of macrophages in vitro (bulk sequencing of treated vs. untreated macrophages) **e**, As in Fig. 3d, compares the effect of APOE on macrophages inferred by *ContactTracing* as compared to ligand effect measured in bulk for two additional, expressed receptors that compliment APOE (Sdc1 and Ldlr). R^2^ is Pearson’s correlation coefficient, p-value is two-sided and testing for correlation.

We performed multiple orthogonal validations of ligand effects predicted by ContactTracing. First, we compared target genes inferred by ContactTracing with those previously reported in experimental assays^23^ (Methods). ContactTracing predicted many transcriptional responses, including those that were context-dependent and could not be inferred from in vitro cytokine assays, such as target genes induced in CCR2-expressing macrophages upon activation in vivo^23,24^ (Extended Data Fig. 5a–c). Second, we observed significant correlation between empirically derived transcriptional responses inferred from bulk RNA sequencing (RNA-seq) of ligand (in this case APOE)-treated and untreated cells (RAW264.7 macrophages) (Extended Data Fig. 5d) and those predicted by ContactTracing using scRNA-seq of APOE-treated RAW264.7 cells only (Fig. 3d and Extended Data Fig. 5e).

**Extended Data Fig. 6:**
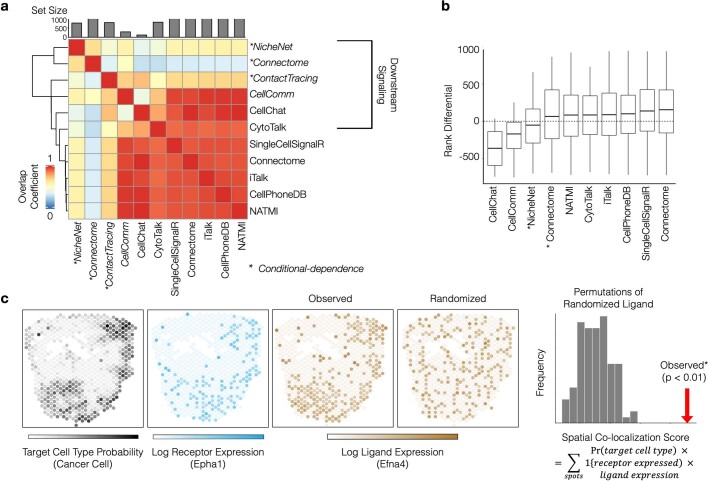
*ContactTracing* benchmarking. **a**, Clustered Heatmap of the overlap coefficient between top 1,000 CIN-dependent interactions predicted by *ContactTracing* and existing methods for inferring cell-cell interactions from single cell data. Alternate tools were run (Methods) using the same ligand-receptor database as *ContactTracing* with “differential” workflows when possible, and results were aggregated and ranked by using the best score for every receptor/cell type combination regardless of ligand source. **b**, Distributions of rank differences between the top CIN-dependent interactions predicted by *ContactTracing* and other methods for inferring cell-cell interactions from single cell data. Rank differences are included for top 1000 interactions (unless fewer detected, exceptions listed) predicted by the following methods: CellChat (n = 116 interactions), CellComm (n = 282 interactions), NicheNet (n = 746 interactions), Differential Connectome, NATMI, CytoTalk (n = 788 interactions), iTALK, CellPhoneDB, SingleCellSignalR, and Connectome. Boxes range from 1^st^ to 3^rd^ quartile, with median indicated, and whiskers extending to min/max of each distribution (there were no outliers). **c**, Co-localization was determined by summing the product of log_10_(ligand expression), probability(receptor cell type) and indicator(receptor expressed) per spot in each sample, and computing a one-sided p-value by comparing this value to 100 permutations in which ligand expression is permuted in the spatial data; co-localized interactions have p < 0.05.

To benchmark our approach, we compared the top 1,000 CIN-dependent interactions predicted by ContactTracing with those identified by existing cell–cell interaction methods (Methods). Similar to other methods that considered downstream signalling, interactions predicted by ContactTracing were largely orthogonal to those predicted by methods that merely relied on the mutual expression of ligand–receptor pairs (Extended Data Fig. 6a,b). An analysis of human TNBC scRNA-seq data^25^ likewise revealed many unique CIN-dependent interactions predicted by ContactTracing (Fig. 3e). We then used matched spatial transcriptomics data to determine the veracity of these interactions. Strikingly, many unique predictions made by ContactTracing were found to colocalize on spatial transcriptomics data from the same human tumour samples (Fig. 3e and Extended Data Fig. 6c). Furthermore, ContactTracing prioritized interactions in a way that better captured their probability of colocalization on spatial transcriptomics data (Fig. 3f).

## Immune suppression from endoplasmic reticulum stress

**Fig. 4:**
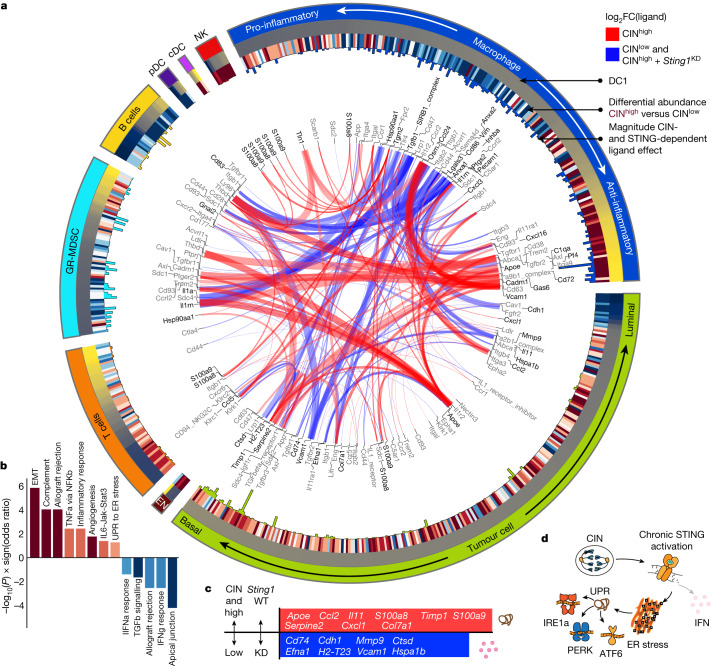
ContactTracing identifies ER stress as a central mediator of CIN-induced immune suppression. **a**, ContactTracing Circos plot highlighting all CIN- and STING-dependent interactions. Each segment represents a cell type, and cell types are further divided into ligands and receptors, which are ordered according to the first diffusion component (DC1) computed on differentially expressed genes (DEGs) in each cell type conditioned on ligand/receptor expression. Outer rings encode CIN-dependent interactions, which include target (receptor-expressing) cells distinguished by ≥10 CIN-dependent interaction effects (two-sided *P* value, FDR *Q* value < 0.25), as well as CIN-dependent ligands complementing those receptors (FDR *Q* value < 0.05 and abs(log_2_(ligand expression FC) > 0.12)). The outer circle represents cell type. The next circle shows the DC1 score for ligand/receptor represented at that coordinate; for example, macrophage response states were organized from pro-inflammatory to anti-inflammatory polarization states. The next circle shows the correlation between the log-normalized expression of that ligand/receptor and its CIN-dependent differential abundance (log_2_(FC) as computed by Milo in local neighbourhoods and mapped to single cells as the described in the Methods). The histogram in the next inner circle shows the number of significant CIN-dependent interaction effects (FDR *Q* value < 0.25). Ribbons in the middle link interacting [ligand, donor cell type] and [receptor, target cell type] pairs; ribbon thickness is proportional to the number of genes exhibiting a CIN- and STING-dependent interaction effect (whichever is greater) and colour represents CIN- and STING-dependent log_2_(FC) of its complementary ligand measured in the donor cell type (whichever is greater). Links are only shown if they exhibit (1) CIN- and STING-dependent expression of ligand in donor cells (in the same direction with FDR *Q* value < 0.05 and abs(log_2_(expression FC) > 0.12)) and (2) at least 10 CIN-dependent and 10 STING-dependent interaction effects in the target cell type. Ligands/receptors are labelled at ribbon ends; ligands are in black and receptors in grey. The data encoded in the ContactTracing Circos plot are provided in Supplementary Table 9 and may be explored interactively at http://contacttracing.laughneylab.com/circos. **b**, Differentially expressed pathways associated with CIN- and STING-dependent, tumour-derived ligands that effect the TME with nominal *P* < 0.05. The *y* axis is scaled by −log_10_(*P* values) times the sign of the odds ratio and colour indicates the pathway odds ratio. **c**, Bar plot highlighting CIN- and STING-dependent tumour-derived ligands that affect the TME, as described in **a**. **d**, Schematic illustrating the impact of chronic STING activation on functions associated with ligand effects.

All CIN- and STING-dependent cell–cell interactions were then visualized for cell pairs (Fig. 3g) or across all major cell types in the TME using a Circos plot (Fig. 4a). Cell–cell interactions in CIN^high^ tumours largely involved cancer cells, immune-suppressive macrophages, Gr-MDSCs and dysfunctional T cells (Extended Data Fig. 4h). Tumour cell-derived factors contributing to these interactions had well-established roles in immune suppression and metastasis, including *Ccl2*, *Cxcl1*, *Il11*, *Apoe* and *Serpine2* (refs. ^26–30^) (Fig. 4a,c). Conversely, CIN^low^ tumours were characterized by interaction between tumour cells, pro-inflammatory macrophages, and helper and cytotoxic T cells (Extended Data Fig. 4h).

**Extended Data Fig. 7:**
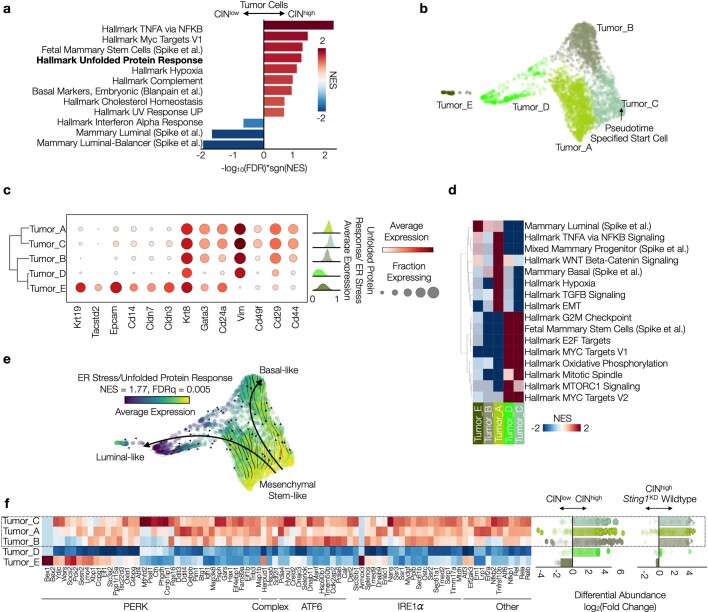
ER stress signaling enriched in mesenchymal stem-like tumor cells. **a**, Directed bar plot showing relevant tumor gene signatures (annotated in Supplementary Table 7) differentially expressed in a CIN-dependent manner (FDR < 0.25). The x-axis shows the -log_10_(FDR) and bar color is scaled by the normalized enrichment score (NES) of the gene signature. **b**, Force-directed layout of all tumor cells (n = 3,596 cells) colored by Louvain subtype. **c**, Dot plot showing relative frequency of tumor cells expressing canonical stem cell and lineage markers (any counts detected) and the average log-transformed expression of each gene per subtype. Hierarchical clustering of genes and subtypes is computed using the complete linkage of the Pearson correlation matrix. Average expression of Hallmark Unfolded Protein Response (annotated in Supplementary Table 7) for all cells per tumor subtype (*right*). **d**, Clustered heatmap showing the normalized enrichment score (NES) of gene signatures differentially expressed within at least one tumor subtype, having a positive NES with FDR < 0.001. Signatures not meeting the FDR threshold are opaque. Complete DEG and GSEA results per tumor cell subtype (relative to all other tumor cells), including nominal and corrected p-values, are provided in Supplementary Table 8. **e**, Force-directed layout with transition vectors of all tumor cells colored by average log-transformed expression of the Hallmark Unfolded Protein Response (UPR) gene signature. Overlayed arrows show two major branches from a more mesenchymal stem-like phenotype to a more luminal or basal state. **f**, Z-normalized average imputed expression of Unfolded Protein Response/ER stress related genes across tumor subclusters. Genes groups are organized according to transcriptional arms of the Unfolded Protein Response to ER stress and clustered within gene groups using the average cosine distance. Tumor subtypes are hierarchically clustered using the average Euclidean distance. *Right*, strip plots showing CIN- and STING-dependent differential abundance (two pairwise-comparisons), log2(fold change), of tumor subpopulations ranked along the subtype hierarchically clustering (*left*). Node opacity is scaled by p-value, so that more significant differential abundance neighborhoods are darker. Bar plots show the mean log_2_(fold change) of neighborhoods with significant (p-value <= 0.1) differential abundance scores; if fewer than two significant neighborhoods detected, all neighborhoods are used in computing the mean.

Interestingly, CIN- and STING-dependent ligands that measurably impacted recipient cells in the TME were associated with an unfolded protein response (UPR) to endoplasmic reticulum (ER) stress, in addition to canonical pathways associated with CIN such as NF-κB and IL6-Jak-Stat3 signalling^2,31^, whereas effectual ligands emanating from CIN^low^ or *Sting1*-depleted CIN^high^ tumour cells were associated with IFN responses (Fig. 4b–d). Accordingly, pairwise comparison of CIN^high^ and CIN^low^ tumour cells revealed significant enrichment of ER stress-related and NF-κB target genes and reduced IFN signalling (Extended Data Fig. 7a). On the other hand, pairwise analysis between CIN^low^ and *Sting1*-depleted CIN^high^ tumour cells did not reveal significant enrichment in the ER stress (normalized enrichment score (NES) = −0.85, false discovery rate (FDR) = 0.83) or type I IFN (NES = 0.56, FDR = 0.95) pathways, suggesting that *Sting1* depletion abolishes CIN-dependent effects in tumour cells. Transcriptional targets of all three arms of the ER stress response^32^ were upregulated in basal stem-like tumour cells that were enriched in CIN^high^ tumours relative to the luminal-like subpopulations that primarily belonged to CIN^low^ and *Sting1*-depleted CIN^high^ tumours (Extended Data Fig. 7b–f).

## STING is required for ER stress response

**Fig. 5:**
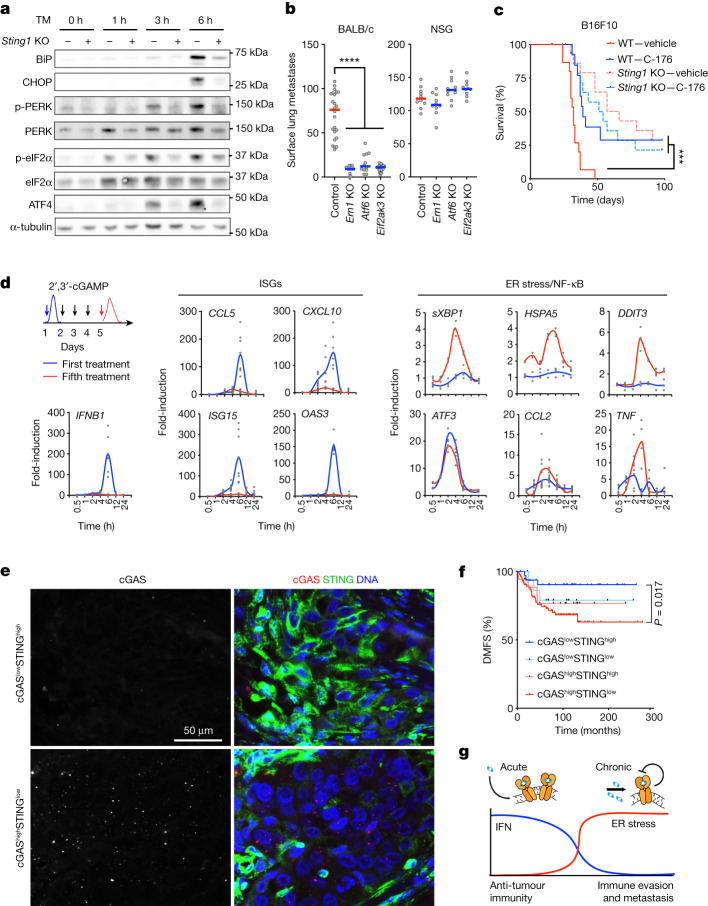
Chronic STING activation promotes IFN tachyphylaxis and ER stress-dependent transcription. **a**, Immunoblots for BiP, CHOP, phosphorylated PERK, total PERK, phosphorylated eIF2α, total eIF2α and ATF4 of 4T1 WT and *Sting1*-KO cells at indicated time points post TM treatment with α-tubulin as loading control. **b**, Number of surface lung metastases in BALB/c or NSG mice that were tail-vein-injected with control 4T1 cells or cells lacking key mediators of the ER stress response; bars represent the median; ****P < 0.0001, two-sided Mann–Whitney test; *n* = 12–24 and 10 animals per group for the BALB/c and NSG injected hosts, respectively. **c**, Survival of C57BL/6 mice upon tail-vein inoculation of WT or *Sting1*-KO B16F10 cells with C-176 or a corresponding vehicle control, log-rank test; ***P < 0.001; *n* = 15 animals per arm. **d**, Relative expression levels of ISGs and ER stress/NF-κB target genes at indicated time points after the first (blue) and the fifth (red) cGAMP stimulations of IMR90 human lung fibroblasts. **e**, Representative images from the same TNBC tumour stained using DAPI (DNA), anti-cGAS and anti-STING antibodies, illustrating the inverse correlation between the frequency of cGAS^+^ micronuclei and STING expression in cancer cells. **f**, DMFS of patients with TNBC stratified based on tumour cGAS and STING expression intensity, log-rank test; *n* = 159 patients. **g**, Schematic illustrating the functional consequences of acute and chronic STING signalling. Source Data

**Extended Data Fig. 8:**
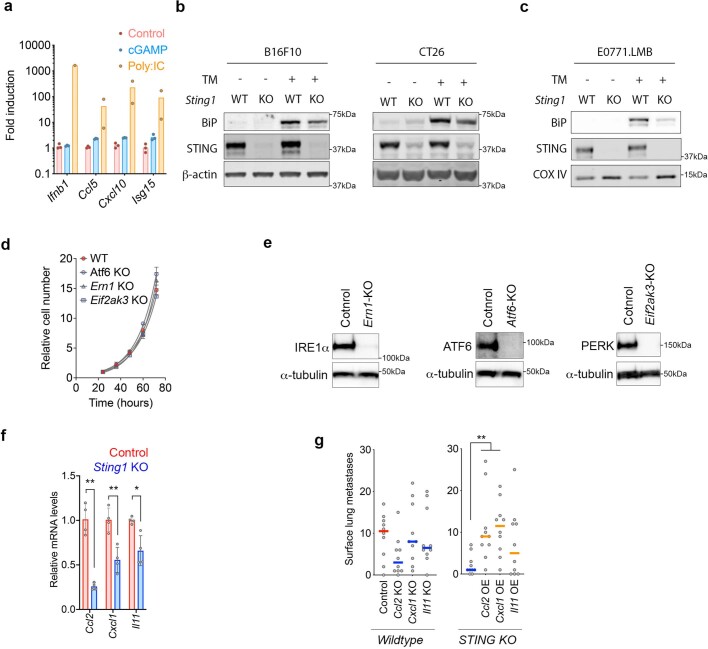
Tumor progression through STING-dependent ER stress response. **a**, Relative expression levels of Ifnb1 and ISGs in mock, cGAMP, or Poly(I:C)-transfected 4T1 cells; bars represent mean ± SD, two-way ANOVA test, n = 3 independent experiments (n = 2 for Poly(I:C)-treated), each with two technical replicates. **b-c**, Immunoblots for BiP and STING of WT and *Sting* KO cells of B16F10, CT26 (**b**) and EO771.LMB (**c**) with β-actin (**b**) or COX-IV (**c**) as loading control. **d**, Cellular growth curves for of Control and IRE1α (*Ern1*), PERK (*Eif2ak3*), or ATF6 (*Atf6*)-KO 4T1 cells; data are presented as mean values ± SD., n = 3 per condition. **e**, Immunoblots of Control and IRE1α (*Ern1*), PERK (*Eif2ak3*), or ATF6 (*Atf6*)-KO 4T1 cells blotted for IRE1a, PERK, ATF6, and α-tubulin as a loading control. **f**, Relative expression levels of *Ccl2, Cxcl1*, and *Il11* in tumor cells isolated from primary tumors resected on day 7; bars represent mean values ± SD, * p < 0.05, ** p < 0.01, two-sided t-test, n = 4 animals per group. **g**, Number of surface lung metastasis in mice inoculated with control 4T1 cells or cells lacking cytokines (left) or *Sting1*-depleted cells or *Sting1*-depleted cells overexpressing cytokines (right); bars represent the median, ** p < 0.01, two-sided Mann-Whitney test, n = 10 animals per group. Source Data

Despite constitutive cGAS–STING activation, CIN^high^ cells exhibited low baseline expression of IFN-stimulated genes (ISGs), with minimal induction upon treatment with exogenous cGAMP but not with Poly(I:C), an activator of the dsRNA sensing pathway, which led to a robust induction of ISGs (Extended Data Fig. 8a). We then treated CIN^high^ cells (4T1, B16F10, EO771.LMB and CT26) with tunicamycin (TM), an ER stress inducer, which promoted robust and time-dependent ER stress response signalling (Fig. 5a and Extended Data Fig. 8b,c). Notably, ER stress response signalling was blunted in *Sting1*-KO cells (Fig. 5a and Extended Data Fig. 8b,c). We next knocked out each of the three main ER stress sensors, IRE1α (*Ern1*), PERK (*Eif2ak3*) or ATF6 (*Atf6*), using CRISPR–Cas9 ribonucleoprotein transfection in 4T1 cells and observed a significant reduction in the number of surface lung metastases after tail-vein inoculation, without impacting cellular proliferation rates (Fig. 5b and Extended Data Fig. 8d,e). Strikingly, this effect was again entirely dependent on the immune system (Fig. 5b).

**Extended Data Fig. 9:**
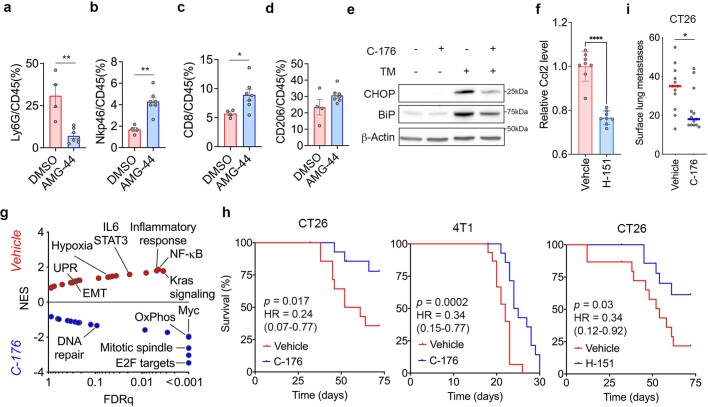
Pharmacological suppression of STING and ER stress attenuates tumor progression. **a–d**, Abundance of Gr-MDSCs (**a**), NK-cells (**b**), CD8+ T-cells (**c**), and M2-like macrophages (**d**) in freshly resected CIN^high^ 4T1 14-day-old tumors treated with vehicle or AMG44 a PERK inhibitor; bars represent mean values ± s.e.m, * *p* < 0.05, ** *p* < 0.01, 2-sided t-test, n = 4 (vehicle) or 7 (AMG44). **e**, Immunoblots for CHOP and BiP in 4T1 cells with or without tunicamycin treatment in the presence STING inhibitor C-176 or vehicle with β-actin as loading control. **f**, Relative Ccl2 production levels in vehicle and H-151 treated 4T1 cells; bars represent mean ± SD, **** *p* < 0.0001, two-sided Welch’s t test, n = 8. **g**, Gene-set enrichment analysis (GSEA) results showing HALLMARK gene sets that are differentially enriched between vehicle and C-176-treated B16F10 cells, one-sided weighted Smirnov-Kolmogorov test corrected for multiple tests. **h**, Animal survival upon tail vein inoculation of CT26 or 4T1 cells in BALB/c hosts that were treated with C-176, H151 or a corresponding vehicle control, two-sided log-rank test, n = 15 animals per experimental arm. **i**, The number of surface lung metastases after tail vein inoculation of CT26 cells; bars represent median values, * *p* < 0.05, two-sided Mann-Whitney test, n = 12-13 animals per group. Source Data

Next, we examined the expression of three ER stress-related cytokines identified from ContactTracing, *Ccl2*, *Cxcl1* and *Il11*, in 4T1 cells and validated their dependence on tumour-intrinsic STING activation (Extended Data Fig. 8f). While KO of individual cytokines in CIN^high^ 4T1 cells was not sufficient to significantly suppress metastasis, overexpression of either *Ccl2* or *Cxcl1* led to a significant increase in metastasis of *Sting1*-KO cells (Extended Data Fig. 8g). Treatment of CIN^high^ tumours with AMG44, a selective PERK inhibitor, led to a significant decrease in Gr-MDSCs and a corresponding increase in NK cells and CD8^+^ T cell infiltration, yet did not measurably impact macrophage polarization (Extended Data Fig. 9a–d).

## STING inhibitors suppress metastasis

Given that signalling downstream of STING in chromosomally unstable cancer cells is skewed towards an ER stress response as opposed to its canonical IFN function, we reasoned that STING inhibition might represent a viable therapeutic strategy in tumours with CIN. Treatment with C-176, a covalent inhibitor that blocks activation-induced palmitoylation of STING^33^, dampened ER stress response signalling, as evidenced by lower CHOP and BiP protein levels in TM-treated CIN^high^ 4T1 cells, and reduced baseline CCL2 levels in conditioned media (Extended Data Fig. 9e,f). Transcriptomic analysis of C-176-treated B16F10 CIN^high^ cells revealed downregulation of pathways related to inflammation, epithelial-to-mesenchymal transition, as well as the UPR/ER stress response (Extended Data Fig. 9g). We next delivered C-176 or H-151, a second covalent STING inhibitor, through daily intraperitoneal injections to tumour-bearing immune competent animals after tail-vein inoculation of CIN^high^ 4T1, B16F10 or CT26 tumour cells. In all instances, treatment with C-176 or H-151 prolonged survival (Fig. 5c and Extended Data Fig. 9h). We necropsied another subset of animals 13 d after inoculation of CT26 cells and observed a significant reduction in surface lung metastases (Extended Data Fig. 9i). Reduced metastasis by the STING inhibitor did not match complete *Sting1* KO, and this might be due to incomplete target exposure by the drug or dichotomous contributions of cancer cell and host cell STING, both of which would be inhibited with drug treatments. We thus administered C-176 to C57BL/6 mice inoculated with *Sting1*-KO B16F10 cells. In these mice, C-176 treatment did not provide an additional survival advantage beyond *Sting1* KO (Fig. 5c). Prolonged daily treatment with the STING inhibitor was well tolerated and did not lead to any clinically evident toxicity when compared with vehicle-treated control animals.

## IFN tachyphylaxis downstream of STING

**Extended Data Fig. 10:**
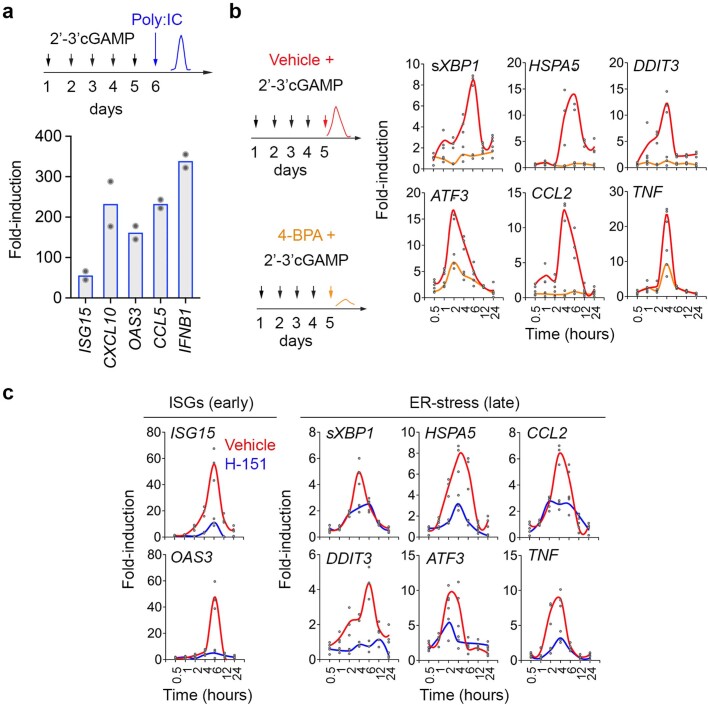
Repetitive cGAMP stimulation reveals signal re-wiring downstream of STING. **a**, Relative expression levels of Ifnb1 and ISGs in mock, cGAMP, or Pol(I:C)-transfected 4T1 cells; bars represent mean, n = 3 independent experiments (n = 2 for Poly(I:C)-treated), each with two technical replicates. **b**, Relative expression level of ER-stress genes in IMR90 cells after the fifth cGAMP stimulation in the presence of 4-Phenylbutyric Acid (4-BPA, orange) or vehicle (red). **c**, Relative expression level of interferon-stimulated genes (ISGs) after the first cGAMP stimulation and ER-stress/NF-κB target genes after the fifth cGAMP stimulation in IMR90 cells in the presence of STING inhibitor H-151 (blue) or vehicle control (red).

To better define the context-dependent nature of cellular responses to STING activation, we developed a tractable model system using non-immortalized IMR90 human lung fibroblasts, which have an intact cGAS–STING pathway that is unstimulated at baseline, yet primed to respond upon cGAMP treatment^34^. We treated IMR90 fibroblasts with cGAMP for five consecutive daily doses and assessed time-dependent expression of key ISGs and ER stress response target genes after the first and fifth daily doses of cGAMP. We observed expected induction of *IFNB1* and ISGs after the first cGAMP treatment (Fig. 5d). However, by the fifth daily treatment, the expression of ISGs was nearly completely abolished (Fig. 5d). This reduction in IFN responsiveness to repetitive stimulation—a process known as tachyphylaxis—was limited to STING, as transfection with Poly:IC after the fifth cGAMP stimulation led to an acute and robust ISG induction (Extended Data Fig. 10a), mirroring observations derived from cancer cells (Extended Data Fig. 8a). Conversely, repetitive treatment with cGAMP led to increased expression of ER stress and NF-κB target genes (Fig. 5d), which was abolished when cells were cotreated with the chemical chaperone and ER stress inhibitor 4-phenylbutyric acid (4-BPA) (Extended Data Fig. 10b). Treatment of IMR90 fibroblasts with the STING antagonist H-151 reduced both acute (early) and chronic (late) STING-dependent effects (Extended Data Fig. 10c).

**Extended Data Fig. 11:**
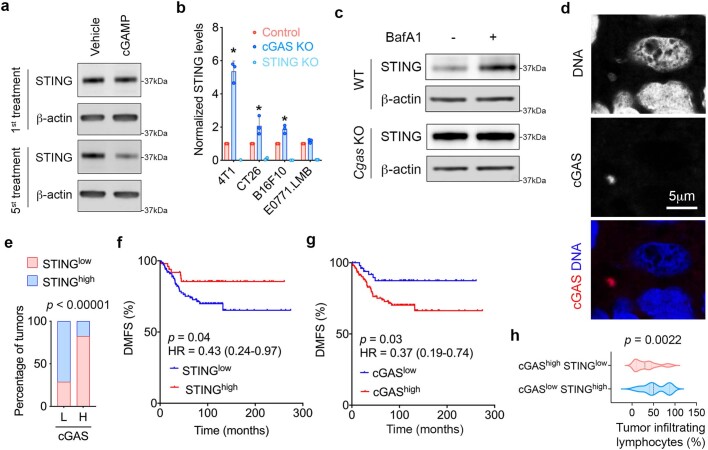
Chronic cGAS–STING activation forebodes poor prognosis in human TNBC. **a**, Immunoblots for STING in IMR90 after the first and fifth cGAMP stimulation with β-actin as loading control. **b**, Normalized STING protein levels in control, *Cgas* KO, and *Sting1* KO cells; bars represent mean ± SD, * p < 0.05, two-sided ratio-paired t-test, n = 3 independent experiments. **c**, Immunoblots for STING of 4T1 WT and *Cgas* KO cells treated with BafA1 or vehicle in the presence of translation inhibitor cycloheximide with β-Actin as loading control. **d**, Representative high-resolution image of human tumor sample stained with DAPI (DNA) and anti-cGAS antibody showing selective localization of cGAS at micronuclei, scale bar 5-μm. **e**, Bar graph depicting the relationship between tumor cGAS and STING protein levels in TNBC, two-sided Chi-Square *χ*^2^-test, n = 179 tumors. **f-g**, Distant metastasis-free survival (DMFS) of patients with TNBC stratified based tumor STING (**f**) and cGAS (**g**) expression intensity, log-rank test, n = 155 patients. **h**, Percentage of tumor infiltrating lymphocytes in TNBC tumors stratified based on protein expression of cGAS and STING, bars represent mean ± s.e.m, n = 16 and 57 patients in the cGAS^low^STING^high^ and cGAS^high^STING^low^ tumors, respectively, two-sided t-test. Source Data

Repeated stimulation of IMR90 cells with cGAMP led to reductions in STING protein levels (Extended Data Fig. 11a), in line with autophagy-lysosomal-dependent degradation of STING mediated by its own activation^35^. Thus, we asked whether CIN-induced chronic STING activation might also explain reduced STING protein levels often observed in cancer cells. Indeed, alleviating chronic activation of STING through *Cgas* KO led to a significant rebound in STING protein levels in three of the four CIN^high^ cancer cell lines examined (Extended Data Figs. 1e and 11b). Furthermore, treatment with the autophagy inhibitor bafilomycin A1 led to an increase in STING protein levels in CIN^high^ WT but not *Cgas*-KO cells (Extended Data Fig. 11c).

## Prognostic relevance of CIN in human TNBC

We then asked whether the inverse relationship between cGAS activity and STING protein levels can be recapitulated in human tumour samples. Using antibodies that were validated on WT and *CGAS*-depleted cell pellets, we observed an inverse correlation between the frequency of cGAS^+^ micronuclei and tumour cell-intrinsic STING expression in human TNBC (Extended Data Fig. 11d,e and Methods). Tumours with a preponderance of cGAS^+^ micronuclei had low, but detectable, STING protein levels within cancer cells (cGAS^high^STING^low^), whereas those with a paucity of cGAS^+^ micronuclei had higher STING protein expression (cGAS^low^STING^high^). This inverse correlation between the expression of cGAS and STING in cancer cells was also observed within spatially heterogeneous tumours (Fig. 5e). cGAS^high^STING^low^ tumours exhibited fewer tumour infiltrating lymphocytes and were associated with reduced distant metastasis-free survival (DMFS), whereas cGAS^low^STING^high^ tumours had a more favourable prognosis (Extended Data Fig. 11f–h and Fig. 5f). Unlike cancer cells, stromal cells consistently displayed strong STING protein expression without evidence of cGAS^+^ micronuclei.

**Extended Data Fig. 12:**
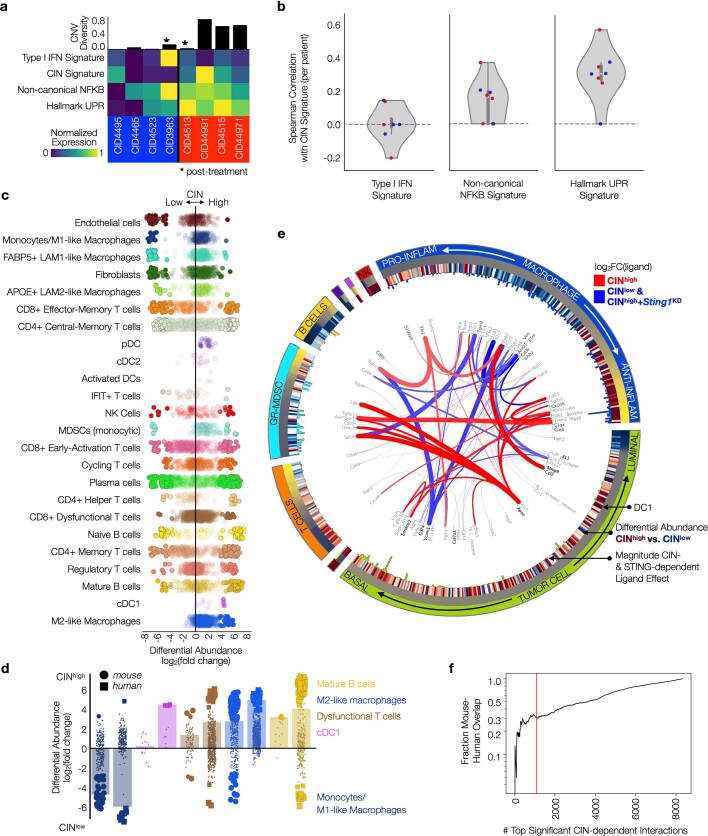
CIN is associated with immune suppression in human tumors. **a**, Clustered heatmap (average Euclidean distance) showing min-max normalized average log-transformed expression of key pathways and tumor cell CNV Diversity (Methods) used to stratify the 8 human TNBC tumors into CIN^low^ and CIN^high^ subsets. **b**, Violin plots for the significant (p < 0.05) within-sample Spearman correlations between the mean CIN signature and mean Type I IFN (*left*), non-canonical NF-kB (*middle*), and hallmark UPR (*right*) signatures computed using all tumor cells within each sample (n = 10,836 cells from 8 human tumors). Nodes are colored by sample condition (blue: CIN^low^, red: CIN^high^) and the overlaid box plots denote the minima and maxima (within 1.5*IQR), median, and 1^st^ and 3^rd^ quartiles. **c**, Strip plot showing CIN-dependent differential abundance within human TNBC cohort. Same as Fig. 2b, but for all cell types in the human TNBC cohort. **d**, Strip plot showing conserved CIN-dependent differential abundance effects (mean enrichment must be both positive or both negative) of cell types in mouse and human TNBC data, ranked by the mean mouse CIN-dependent log_2_(fold change) within each cell subtype. Node size is scaled by p-value, so that more significant differential abundance neighborhoods are larger. Bar plots show the mean log_2_(fold change) of neighborhoods with significant (p-value <= 0.1) differential abundance scores; if fewer than two significant neighborhoods are detected, all neighborhoods are used in computing the mean. **e**, *ContactTracing* circos plot, as in Fig. 4a, intersected with CIN-dependent interactions detected in human TNBC; which are defined as exhibiting CIN-dependent differential expression of the ligand in human tumors (q < 0.05, log_2_FC must be in same direction as CIN- and STING- in mouse analysis), and we detection >= 10 CIN-dependent interaction effects in the target cell type. Data provided in Supplementary Table 9. **f**, Fraction of overlapping CIN-dependent interactions predicted in mouse and human TNBC samples as a function of the top ranked interactions per dataset; evaluated within the subset of interactions that can be mapped between the human and mouse. Each unique interaction (identified by receptor, target cell type, ligand) is ranked by the number of CIN-dependent interaction effects detected in the target cell type, multiplied by the identity function that expression of the ligand is also CIN-dependent in any cell type in the TME.

We then analysed CIN-dependent interaction effects in available scRNA-seq data from eight human TNBCs^25^ using sample-level karyotypic diversity and CIN-associated transcriptional signatures to stratify patient tumours into CIN^high^ and CIN^low^ cohorts (Extended Data Fig. 12a and Methods). There was a consistent cell-level correlation between CIN transcriptional signatures^2^ and cancer cell-intrinsic expression of ER stress-related genes, but not ISGs (Extended Data Fig. 12b), across patients. CIN^high^ tumours were likewise associated with an immune-suppressive TME characterized by enrichment of M2-like macrophages and dysfunctional T cells, whereas CIN^low^ tumours were enriched for M1-like macrophages and monocytes (Extended Data Fig. 12c,d). Finally, we applied ContactTracing to identify CIN- and STING-dependent cell–cell interactions in human TNBCs, and compared these with CIN-dependent interactions predicted in the mouse (Extended Data Fig. 12e,f). Many conserved interactions involved tumour ligands associated with ER stress, such as *APOE*, *IL11* and *CCL2*.

## Discussion

CIN and STING activation are poorly tolerated in normal cells, where they often promote cellular senescence and immune-mediated clearance^36–38^. This led to the idea that CIN may act as a tumour suppressor^12,13^. Furthermore, STING activation has been proposed as a checkpoint against cellular transformation^39,40^ or the re-awakening of dormant metastasis^41^. Paradoxically, advanced and metastatic human tumours often exhibit evidence for CIN, and, in this context, it is associated with immune evasion^2,7,8,14,42^. Similarly, in tumour models, CIN and persistent STING activation were shown to promote tumour cell survival as well as drive cancer progression, metastasis and immune suppression^2,4,31,34,43–49^. This dichotomy invokes key adaptive steps that must take place for cancer cells to tolerate—and co-opt—ongoing chromosome missegregation and downstream inflammatory signalling. Rather than the wholesale loss of STING protein from cancer cells, our data argue that the most parsimonious path toward tumour progression and metastasis is adaptive re-wiring of signalling downstream of STING—a process that can occur within days, thereby allowing tumours to simultaneously eschew the deleterious pro-inflammatory role of type I IFN while benefiting from immune-suppressive ER stress signalling (Fig. 5g).

Activators of the STING pathway are currently in clinical development^50,51^. The IFN-specific tachyphylaxis observed upon chronic STING activation, along with an immunosuppressive TME, might explain pre-existing resistance of chromosomally unstable tumours to STING agonists, which have thus far demonstrated limited efficacy in early-stage clinical trials despite evidence for adequate target engagement^50,51^. Critically, our results pave the way for a biomarker-based approach to stratify patients whose tumours still maintain the ability to mount an acute IFN-dominant response to STING activation (cGAS^low^STING^high^, Fig. 5e,f). Our paradigm also recognizes a subset of patients who might instead benefit from inhibition of cGAS–STING signalling to curb tumour-intrinsic chronic inflammation and its immune-suppressive sequalae (cGAS^high^STING^low^, Fig. 5e,f). Given ongoing efforts to develop selective inhibitors of cGAS, STING^33,52^ and ER stress sensors, such as PERK^53^, our work offers an exciting opportunity for therapeutic intervention in chromosomally unstable tumours for which there are currently few effective therapeutic options.

## Methods

### Cell culture

IMR90, 4T1, CT26, RAW264.7 and B16F10 cell lines were purchased from the American Type Culture Collection and cultured in MEM (IMR90), DMEM (B16F10, RAW264.7) or RPMI (4T1, IMR90, CT26) supplemented with 10% FBS in the presence of penicillin (50 U ml^−1^) and streptomycin (50 μg ml^−1^). All cells were found to be negative for mycoplasma upon repeated routine testing.

### The generation of KO and gene-overexpressing cell lines

Murine cancer cells with *Cgas*, *Sting1*, *Atf6*, *Ern1*, *Eif2ak3*, *Ccl2*, *Cxcl1* and *Il11* KO were generated by Cas9 ribonucleoprotein nucleofection using a Lonza 4D-Nucleofector and SF Pulse Code CM-150 Cell Line Kit. For *Cgas* and *Sting1* KO, four guides were screened per target and KO cell lines were confirmed using immunoblotting. For *Atf6*, *Ern1* and *Eif2ak3* KO, three guides were used simultaneously. For *Ccl2*, *Cxcl1* and *Il11* KO, two guides were used sequentially. Stable knockdown of *Cgas* or *Sting1* in 4T1 cells was achieved using shRNAs in pRRL (SGEP) plasmids obtained from the Memorial Sloan Kettering Cancer Center (MSKCC) RNA Interference Core. Four distinct shRNA hairpins were screened per target. Targeted shRNA and CRISPR guide RNA sequences are listed in Supplementary Table 1. To overexpress Kif2c or dnMCAK, Kif2c and dnMCAK complementary DNA sequences were cloned into the pEGFP vectors, which, then, were transfected to 4T1 cells. Cells were selected using 2 µg ml^−1^ puromycin. To exogenously express *Sting1*, *Cxcl1*, *Ccl2 or Il11*, cDNAs were cloned into viral pLenti-EF1a-Bsd-P2A vector and were transduced with the lentiviral system.

### cGAMP quantification

For cGAMP quantification in cell lysates, cancer cells were seeded in 15-cm culture dishes. When culture plates were 80–90% confluent, cells were washed with PBS twice then trypsinized for 5 min at 37 °C, and cells counts were measured. Cells were then centrifuged at ≥600*g* at 4 °C for 15 min. Whole cell lysates were generated by lysing the cell pellet in LP2 lysis buffer (Tris HCl pH 7.7 20 mM, NaCl 100 mM, NaF 10 mM, β-glycerophosphate 20 mM, MgCl_2_ 5 mM, Triton X-100 0.1% (v/v), glycerol 5% (v/v)). The homogenate was then subjected to centrifugation at 10,000*g* for 15 min. For tumour samples, the tumour tissues were homogenized in LP2 lysis buffer (1:10 w/v) with homogenizer. The homogenate was then subjected to centrifugation at 10,000*g* for 15 min. cGAMP ELISA was performed according to the manufacturer’s protocol using DetectX Direct 2′,3′-Cyclic GAMP Enzyme Immunoassay Kit (Arbor Assay).

### Immunoblotting

Cells were pelleted and lysed using RIPA buffer. Protein concentration was determined using BCA protein assay and 20–30 μg of total protein was loaded in each lane. Proteins were separated by gradient SDS–PAGE and transferred to PVDF or nitrocellulose membranes. Membranes were blocked with TBST buffer containing 5% BSA for 1 h and incubated with the primary antibody in 5% BSA TBST overnight at 4 °C. The primary antibody information is listed in Supplementary Table 2. After three washes with TBST, membranes were incubated with proper horseradish peroxidase (HRP)- or fluorescent dye-conjugated secondary antibodies in TBST containing 3% BSA for 1 h at room temperature. After three washes with TBST, membranes using fluorescent dye-conjugated secondary antibodies were imaged using the LI-COR Odyssey system. For membranes using HRP-conjugated secondary antibodies, signal was visualized using SuperSignal West Femto Maximum Sensitivity Substrate by Amersham Imager. Relative STING protein levels were quantified by measuring band intensities on immunoblots using ImageJ software, background subtracted and normalized to a loading control.

### Immunofluorescence microscopy

Cells were fixed with ice-cold (−20 °C) methanol for 15 min. Subsequently, cells were permeabilized using 1% Triton for 4 min. The primary antibody information is listed in Supplementary Table 3. TBS-BSA was used as a blocking agent during antibody staining. DAPI was added together with secondary antibodies. Cells were mounted with Prolong Diamond Antifade Mountant (Life Technologies, P36961).

### H&E staining of lung metastases

Lungs were excised from euthanized mice and submerged in 4% PFA overnight at 4 °C, and then were transferred to 70% ethanol. Tissue embedding, slide sectioning and H&E staining were performed by the Molecular Cytology Core Facility at MSKCC.

### Quantitative PCR

RNA was extracted from cells with Trizol (Invitrogen no. 15596026). cDNA was synthesized using the RNA to cDNA EcoDry Premix (Double Primed) kit (Takara no. 639549). Real-time PCR was performed to measure the relative messenger RNA expression levels of ISGs and the control GAPDH using Luna Universal qPCR Master Mix (NEB M3003L). The quantitative PCR reaction and analysis were performed on a QuantStudio 6 platform (Life Technology). The primer sequences are listed in Supplementary Table 4. Relative expression of analysed genes was determined, normalizing to human *Gusb* or mouse *Actb* housekeeping gene expression.

### Cell stimulation with APOE for bulk and single-cell RNA-seq

For APOE treatment assays, 1 × 10^5^ RAW264.7 cells were seeded in 24-well plates or 5 × 10^5^ RAW264.7 cells were seeded in 6-well plates. After 36 h, when culture plates were 80–90% confluent, medium with APOE (3 μg ml^−1^) was added to the wells for 2 h. For scRNA-seq experiments, treated and non-treated cells from 24-well plates were mixed at equal cellular concentrations to generate 5,000 Gel Bead-In-Emulsions (GEMs), with an average initial cell viability of 93%. RNA purification from the cells seeded in six-well plates was performed using the Monarch Total RNA Miniprep Kit (New England BioLabs), and samples with high-quality RNA (RNA integrity number > 8.5) as measured using 2200 TapeStation (Agilent Technologies) were used for bulk RNA-seq library preparation. cDNA was processed with TruSeq Stranded mRNA Library Preparation Kit (Illumina, 20020594) and sequenced with a NextSeq2000 instrument.

### In vitro TM treatment

For TM treatment, 0.5 × 10^4^ cells were seeded in 6-well plates. When cell confluence reached 70 per cent, media containing indicated concentrations of TM (63 ng ml^–1^ for 4T1, 126 ng ml^–1^ for CT26, 210 ng ml^–1^ for B16F10 and 84 ng ml^–1^ for EO771.LMB) or dimethylsulfoxide were added. Cell lysates were collected at indicated time points and were analysed (12 h for CT26, 11 h for B16F10 and 10 h for EO771.LMB). For the C-176 pretreatment experiment, cells were pretreated with 1 μM C-176 or vehicle for 3 weeks, during which the medium was replaced with freshly prepared medium with C-176 or vehicle and cells were split every 3 d. When cells were treated with TM and vehicle, C-176 and its vehicle were also present in the medium during treatment.

### In vitro cGAMP stimulation

IMR90 cells were seeded at a density of 1 × 10^4^ cells per well in 6-well plates on day 0. For single-dose cGAMP stimulation, medium was replaced with medium containing 10 μM cGAMP. For repetitive stimulation, medium was replaced with fresh medium containing cGAMP every day. Gene expression analysis and immunoblots were performed as described before. For 4-BPA (Enzo Life Technologies) treatment, cells were stimulated with cGAMP in the presence of 5 mM 4-BPA. For STING inhibitor treatment, cells were pretreated with 0.5 μM H-151 (Invivogen) followed by stimulation with cGAMP in the presence of H-151. For the poly(I:C) stimulation, cells were stimulated by transfecting 2 μg ml^−1^ poly(I:C) for 6 h at 24 h after the fifth cGAMP stimulation.

### Autophagy inhibition by BafA1

In 6-well plates, 0.5 × 10^6^ 4T1 WT and *Cgas*-KO cells were seeded per well on day 0. On day 1, cells were treated with 0.5 μM BafA1 or vehicle together with 25 μg ml^−1^ cycloheximide. Cell lysates were collected and were analysed as described before.

### NK killing assay

Primary NK cells were isolated from splenocytes of nude athymic mice using EasySep mouse NK cell isolation kit (Stemcell Technologies, 19855) in accordance with the manufacturer’s protocol. The isolated NK cells were then seeded with tumour cells at a ratio of 1:10 (tumour: NK cells) in media supplemented with 20 ng ml^−1^ IL-12 (BioLegend, 577002) and 10 ng ml^−1^ IL-15 (BioLegend, 566302). After 16 h of coculture, wells were washed with PBS twice to remove dying tumour cells and floating NK cells and the remaining adherent tumour cells were collected and counted.

### T cell killing assay

Primary T cells or CD8^+^ T cells were isolated from splenocytes of BALB/cJ mice using EasySep mouse T cell isolation kit (Stemcell Technologies, 19851) or CD8^+^ cell isolation kit (Stemcell Technologies, 19853) in accordance with the manufacturer’s protocol. Isolated T cells or CD8^+^ T cells were activated with 20 ng ml^−1^ IL-2 (BioLegend, 575402) for 24 h before being seeded with tumour cells at a ratio of 1:5 (tumour:T cells/CD8^+^ T cells). After 24 h of coculture, wells were washed with 1 × PBS twice and remaining adherent cells were collected and counted.

### Macrophage polarization assay

Primary macrophages were collected from bone marrow of BALB/cJ mice and differentiated into M1 macrophages as previously described^54^. After 7 d of the differentiation process, differentiated M1 macrophages were cultured with conditioned medium from tumour cells for 24 h. Then, macrophages were collected, and RNA isolation was performed using the RNAeasy mini plus kit (Qiagen, 74134). mRNA expression of Arginase1 from RT–PCR was employed as a proxy measurement of M1 polarization to M2 macrophages.

### Transwell migration assay

Splenocytes collected from spleens of BALB/cJ mice were seeded in the top compartment of a Transwell chamber with 3-µm pore size (Corning, 3462). Tumour cells were seeded in the bottom compartment 24 h before the addition of splenocytes. After 48 h of incubation, media from the bottom compartment were collected and numbers of immune cells were calculated.

### Flow cytometry analysis

Primary tumours arising by implanting 2.5 × 10^5^ GFP-expressing 4T1 cells in 100 μl of PBS:Matrigel (1:1) into the mammary fat pads were resected on day 10. Tumour pieces were digested to single-cell suspensions with Collagenase/Hyaluronidase (Stemcell Technologies, catalogue no. 07912) and DNAase I (Stemcell Technologies, catalogue no. 100-0762) according to the manufacturer’s manual, followed by filtration with 70-µM cell strainers. Cells were stained with Zombie NIR Fixable Viability Kit (BioLegend, catalogue no. 423105) for 10 min on ice, followed by blocking with TruStain FcX (anti-mouse CD16/32) antibody (BioLegend, catalogue no. 101319). Cells were then stained with fluorophore-conjugated antibody solution in PBS containing 2% FBS on ice for 30 min. The primary antibody information is listed in Supplementary Table 5. After washing with PBS, cells were analysed using the Cytek Aurora Flow Cytometry System. Data were analysed with FlowJo software.

### Animal metastasis studies

Animal experiments were performed in accordance with protocols approved by the MSKCC Institutional Animal Care and Use Committee. For survival experiments, power analysis indicated that 15 mice per group would be sufficient to detect a difference at relative hazard ratios of less than 0.25 or more than 4.0 with 80% power and 95% confidence, given a median survival of 58 d in the control group and a total follow-up period of 180 d, accounting for accidental animal death during procedures. For metastasis experiments relying on the tumour burden or lung surface metastasis number, the animal numbers were estimated based on previous experience with these models. For in vivo experiments, animals were randomly assigned to different groups. Investigators were not blinded to group allocation. For tail-vein injections, 5 × 10^4^ 4T1, 1 × 10^5^ 4T1-Luc, 2.5 × 10^4^ B16F10 or 10^5^ CT26 cells were injected into the tail-vein of 6–7-week-old BALB/c (4T1 and CT26) or C57BL/6 (B16F10) female mice. For experiments using immune-deficient mice, 2.5 × 10^4^ 4T1, 1 × 10^5^ 4T1-Luc, 1.25 × 10^4^ B16F10, 5 × 10^4^ CT26 or 2.5 × 10^5^ EO771.LMB cells were injected into 6–8-week-old NSG mice (JAX:005557). Metastasis was primarily assessed through overall survival. Overall survival end point was when the mice died or met the criteria for euthanasia under the Institutional Animal Care and Use Committee protocol. Pain and distress were monitored by observing the presence of rapid weight loss, weight loss exceeding 20% of body weight, hunched posture, lethargy, lack of movement, rapid growth of tumour masses, mass larger than 2 cm^3^, gait abnormalities, lesion interfering with eating and drinking, anuria, ulcerated tumour, change in stool shape and/or size, and vaginal bleeding. Mice exhibiting any of these signs were euthanized. Transplanted tumours were not to exceed 20% in any dimension or 10% of body weight. Surface lung metastases were assessed at end point by direct visual examination after euthanasia, at which point lungs were perfused and fixed in 4% paraformaldehyde (4T1, EO771.LMB and B16F10 experiments) or stained using India ink (CT26 experiments). Furthermore, lung metastasis after injection of 4T1 cells was qualitatively assessed using routine H&E staining. For 4T1 orthotopic tumour implantation, 2.5 × 10^5^ 4T1 cells in 50 μl of PBS were mixed 1:1 with Matrigel (BD Biosciences) and injected into the fourth mammary fat pad. For EO771.LMB orthotopic tumour implantation, 2.5 × 10^5^ EO771.LMB cells in 50 μl of Hanks’ Balanced Salt Solution were implanted. Only one tumour was implanted per animal. Primary tumours were surgically excised on day 7 (4T1) or day 14 (EO771.LMB) after implantation and metastatic dissemination was assessed by monitoring overall survival or through quantification of surface lung metastases upon euthanasia on day 30. The length (*L*) and width (*W*) of the primary tumours were measured using callipers. Tumour size was calculated according to the following formula: *L* × *W*^2^/2.

### Bioluminescence imaging to monitor metastatic progression

4T1 cells were transduced with lentiviral particles encoding firefly luciferase under control of the CAG promoter with an RFP–blasticidin fusion dual selection marker (Amsbio, LVP571). Transduced cells were grown in selection media containing 20 μg ml^−1^ blasticidin for 2 weeks, then sorted for a narrow range of medium RFP expression. Plasmids encoding enhanced specificity SpCas9 (eSpCas9), a customized guide RNA, and GFP were purchased from Genscript (eSpCas9-2A-GFP (PX458)). Guide sequences for murine *Cgas* were: 5′-GGCCAUGCAGAGAGCUUCCG-3′ and 5′-CGAGUCUCCGGCUGCCCCCG-3′. The guide sequence for murine *Trac* was: 5′-UUCUGGGUUCUGGAUGUCUG-3′. For *Cgas*-KO cells, RFP-luc-4T1 cells were transiently transfected with both *Cgas*-targeting plasmids simultaneously. For *Trac* KO (cutting, but non-expressing control) cells, RFP-luc-4T1 cells were transiently transfected with the *Trac*-targeting plasmid. After 2 d, cells were sorted for GFP expression. These cells were allowed to expand for 2 weeks. A second round of transient transfection and GFP-based sorting was performed to obtain polyclonal cell lines with greater than 95% KO efficiency by western blot. Experimental metastasis assays were performed by injecting 100,000 4T1 (Luc-RFP) cells in the tail-vein of female BALB/cJ (Jackson Laboratory, stock no. 000651) mice. For the metastasis assay with NSG mice, 50,000 4T1 (RFP-Luc) cells were injected in the tail-vein of female NSG mice (stock no. 005557). In all experiments, 5–7-week-old mice were used. The cells were re-suspended in PBS and passed through a 70-μm cell strainer and injected in a final volume of 100 μl of PBS. To detect lung metastasis, animals were injected retro-orbitally with 100 μl of luciferin (PerkinElmer, XenoLight d-Luciferin Potassium Salt, catalogue no. 122799) diluted in PBS (final concentration of 16.67 mg ml^−1^). Luminescence was measured twice a week with an IVIS spectrum device (PerkinElmer, CLS136331 IVIS Lumina LT Inst, Series III, 120 V), starting straight after the tail-vein injection on day 0. Mice were checked twice a day and euthanized when showing any signs of illness or distress.

### Analysis of cGAS and STING protein expression in breast tumour samples

Primary analysis of cGAS and STING protein expression was performed on a tissue microarray of 217 formalin-fixed, paraffin-embedded TNBC samples. Samples and follow-up data were collected under MSKCC Institutional Review Board approval. Patients gave consent according to the institutional review board-approved standard operating procedures for informed consent. Written, informed consent was obtained from all patients. The study was conducted in accordance with the Declaration of Helsinki and good clinical practice guidelines. There were three cores per tumour sample. Of the 217 samples, 183 and 180 samples had sufficient material for adequate assessment of cGAS and STING expression levels, respectively. This included 179 samples with adequate expression and quality to simultaneously quantify both proteins. Detailed clinical characteristics and clinical follow-up data were previously reported^55^. Immunohistochemistry for cGAS and STING was performed on the automated Discovery XT processor (Ventana Medical Systems) by the Molecular Cytology Core Facility at MSKCC^56^. Briefly, after deparaffinization and tumour tissue conditioning, the antigen was retrieved using standard CC1 (Ventana Medical Systems). Following blockage with Background Buster (Innovex), the slides were incubated with 1:100 diluted anti-STING antibody for 4 h, and then incubated with the biotinylated secondary antibody for 30 min. The Streptavidin-HRP D kit (DABMap kit, Ventana Medical Systems) and the Alexa Fluor 488 Tyramide SuperBoost Kit, Streptavidin (Life Technologies, catalogue no. B40932) were used to detect the signal according to the manufacturer instructions. A similar procedure was then applied to detect cGAS with 1:100 diluted anti-cGAS antibody and Alexa Fluor 594 Tyramide SuperBoost Kit, Streptavidin (Life Technologies, catalogue no. B40935). Slides were counterstained with haematoxylin and were mounted with Permount mounting medium. Slides of immunofluorescence and immunohistochemistry were scanned with a Pannoramic Flash 250 (3DHistech) with ×20/0.8 numerical aperture air objective by the Molecular Cytology Core Facility at MSKCC. cGAS and STING protein expression levels were assessed manually using scores of 0 (absent), 1 (weak), 2 (moderate) and 3 (strong). STING expression was assessed separately in the tumour and stromal compartments. cGAS was rarely localized to micronuclei in the stroma and therefore was primarily assessed in the tumour compartment. DMFS data were collected by reviewing medical records available at MSKCC. Tumours were categorized as having low (negative or weak) or high (moderate or strong) cGAS or STING expression.

### RNA-seq analysis

B16F10 cells were pretreated with 1 μM C-176 or dimethylsulfoxide for 48 h, and media with fresh drug was added at 24 h. RNA was extracted using the RNeasy Mini Kit (Qiagen, 74104). Non-strand-specific paired-end sequencing libraries were generated with TruSeq Stranded mRNA (Illumina, 20020594) and sequenced on the Illumina NovaSeq platform. Reads were mapped to the mouse reference GRCm38 with the Broad Picard Pipeline (http://broadinstitute.github.io/picard/). Gene expression levels were estimated with GenomicAlignments (v.1.18.1)^57^. Differential analysis was performed by DESeq2 (v.1.24.0)^58^. Gene set enrichment analysis was performed on the normalized reads estimated by DESeq2. Genes downregulated in C-176-treated cells were filtered by two cutoffs: adjusted *P* value less than 0.05 and log_2_-transformed FC (C-176 versus vehicle) less than −1. Genes downregulated in *Sting1* KO were filtered by two cutoffs: adjusted *P* value less than 0.1 and log_2_-transformed FC less than −1.

### Dissociation of murine tumours for scRNA-seq

Animal experiments were performed in accordance with protocols approved by the MSKCC Institutional Animal Care and Use Committee. First, 1.25 × 10^5^ 4T1 cells in 50 μl of PBS were mixed 1:1 with Matrigel (Corning) and injected into the fourth mammary fat pad of 7-week-old BALB/c immune competent mice. Primary tumours were resected under sterile conditions 14 d after orthotopic implantation. The entire tumour was immediately placed in RPMI medium (Corning) on ice and dissociated using both mechanical and enzymatic digestion (Mouse Tumor Dissociation Kit no. 130-096-730, Miltenyi Biotec), generally within 1 h of surgical resection. Tissues were minced with a razor blade in the Miltenyi enzyme mix according to the manufacturer’s specifications and transferred to a Gentle MACS Octo Dissociator with heaters (no. 30-096-427, 37 °C) for further mechanical dissociation. Upon dissociation, cell suspensions were passed through a 70-µm filter and washed twice with FACS buffer (2% heat-inactivated FBS, 1 mM EDTA and Pen/Strep in PBS without Ca or Mg). The remaining cell suspensions were subsequently flow sorted with a BD FACSAria II cell sorter fitted with a 100-µm nozzle to enrich for viable, single cells according to forward and side scattering, and DAPI exclusion. Cells were sorted directly into RPMI medium with 10% FBS, washed three times and re-suspended in PBS with 0.04% BSA for single-cell encapsulation. Final cell concentrations were determined with a haemocytometer.

### scRNA-seq library preparation

The 10X Genomics Chromium platform was used to generate a targeted 5,000 single-cell GEMs per sample, loaded with an average initial cell viability of 87%. scRNA-seq libraries were prepared following the 10X Genomics user guide (Single Cell 3′ V2 Reagent Kits User Guide PN-120233, 10X Genomics). After encapsulation, emulsions were transferred to a thermal cycler for reverse transcription at 53 °C for 45 min, followed by heat inactivation for 5 min at 85 °C. cDNA from the reverse transcription reaction was purified using DynaBeads MyOne Silane Beads (Thermo Fisher Scientific) and amplified for 12 cycles using Amplification mix and primers provided in the Single Cell 3′ reagents module 1 (10X Genomics). After purification with 0.6X SPRIselect beads (Beckman Coulter), cDNA quality and yield were evaluated using Agilent Bioanalyzer 2100. Using a fragmentation enzyme blend (10X Genomics), the libraries were fragmented, end-repaired and A-tailed. Products were double-side cleaned using 0.6X and 0.8X SPRIselect beads, and adaptors provided in the kit were ligated for 15 min at 30 °C. After cleaning ligation products, libraries were amplified and indexed with unique sample index i7 through PCR amplification. The number of PCR cycles was chosen based on cDNA yield for each sample individually. Final libraries were double-side cleaned using 0.6X and 0.8X SPRIselect beads and their quality and size were evaluated using an Agilent Bioanalyzer 2100. Libraries were pooled and sequenced on a HiSeq2500 (Illumina) paired-end read flow cell following recommendations in the 10X Genomics guide, sequenced for 26 cycles on the forward read (10X barcode + unique molecular identifier), followed by 8-base pair I7 index (sample index) and 98 base pairs on the reverse read.

### ContactTracing to identify and map the effects of conditionally dependent cell–cell interactions

ContactTracing exploits inter- and intrasample variability in single-cell data to ask whether putative interactions, identified based on the co-occurrence of complementary ligand–receptor pairs in the TME^59^, indeed yield a transcriptional response in target (receptor-expressing) cells that depends on condition-specific presence of ligand (Fig. 3a–c and Extended Data Fig. 4a–c). The model makes no assumptions about what this ligand effect looks like, but rather infers genes and processes associated with each cellular response based on intrinsic variability in receptor expression (within the target cell type) and ligand abundance in the TME (Extended Data Fig. 4f); here, we focus on ligands that are CIN- or STING-dependent. Finally, we map these cellular response states, that is, ligand effects, back to individual cells to ask whether multiple, distinct tumour subpopulations cooperatively shape the TME and whether their abundance is dependent on perturbation of tumour-intrinsic CIN or *Sting1* (Extended Data Fig. 4g,h).

#### Database of complementary ligand–receptor pairs

To obtain an interaction database as input to ContactTracing, we took the intersection of two databases: CellTalkDb^60^ (http://tcm.zju.edu.cn/celltalkdb/download.php, accessed 26 March 2021) and the database used by the CellPhoneDb^59^ (v.2.1.4) method. CellTalkDb has both human- and mouse- specific databases, and we used the appropriate one for each species. CellPhoneDb is a human database; for the mouse analysis we mapped the genes to the mouse genome as described in the next section, ‘Mouse to human gene mapping’. CellPhoneDb includes ‘complex’ ligands and receptors, where each complex consists of multiple genes. For any putative complex-mediated interactions, we added a corresponding ‘complex gene’ to our scRNA-seq expression matrix whose expression is the minimum expression of all genes comprising the complex. We removed any interactions where the ligand or receptor were filtered from our scRNA-seq database for low expression. The total mouse interaction database contains 1,885 interactions (1,261 from CellTalkDb, 917 from CellPhoneDB, 293 of which overlap). The total human interaction database contains 2,934 interactions (2,348 from CellTalkDb, 846 from CellPhoneDb; 260 overlapping).

#### Mouse to human gene mapping

Human–mouse orthologs annotated by the Jackson Laboratory (http://www.informatics.jax.org/downloads/reports/HOM_MouseHumanSequence.rpt, accessed 1 March 2021) were used to map 79.3% of our mouse genes to human genes one-to-one. An additional 23 mouse ligands and receptors were mapped to human genes through capitalization, that is, Lgasl9 → LGASL9. Finally, we manually dealt with six human genes that mapped to multiple mouse genes (HLA-A, SIRPB1, KLRB1, LILRB4, SAA1, CSF2RB). After inspecting expression patterns of these multi-mapped genes, we mostly used the average expression across multiple orthologs for each gene to represent that mapped ligand/receptor. The only exception was HLA-A, whose mouse orthologs exhibited several distinct patterns of expression and so was dropped from further analysis.

#### Testing for a transcriptional response in receptor-expressing target cells

We used the BioConductor package MAST^61^ (v.1.14.0) to perform a likelihood ratio test between receptor-expressing (any molecules detected, target^+^) and receptor-null (no molecules detected, target^−^) cells, within the target cell type, across all genes (Fig. 3b,c and Extended Data Fig. 4a,b). We refer to this as the target test. The MAST function, zlm, fits a Hurdle model to the log-normalized expression of each gene using generalized linear regression. We used the regression formula: $Y\approx {\rm{CDR}}+{\rm{condition}}+{\rm{target}}$, where CDR models the cellular detection rate (fraction of genes detected in a cell, an important covariate for modelling single-cell expression data), condition is a categorical variable indicating sample source (CIN^high^, CIN^low^ or *Sting1*^KD^) and target is a binary parameter indicating cell membership in the receptor-expressing subset (target^+^). The zlm function results in parameter estimates for each gene, including log_2_(FC) estimates for how expression relates to condition and target status. We then use MAST’s lrTest function to compute the change in likelihood when target is dropped from the model. This produces a *P* value for each gene indicating whether the model including target as a covariate fits significantly better than a model without. Thus, significant *P* values indicate genes whose expression is different between receptor-expressing (target^+^) and receptor-null (target^−^) subpopulations. We apply the Benjamini–Hochberg procedure to account for multiple hypothesis testing, yielding an FDR value per gene.

#### Testing for condition-specific responses to receptor engagement in target cells

Fitted parameter values from the target test can reflect associations and are not causal if there is unobserved confounding (correlation) between receptor expression and the expression of other genes. However, we may exploit secondary variability in ligand availability across conditions to distinguish genes that are ligand effects from those that happen to be co-expressed with the relevant receptor protein. Thus, for all interactions that involve a ligand that is differentially expressed across conditions (CIN- or STING-dependent in any cell type), we performed a second likelihood ratio test to determine whether model fit improves with the addition of a condition-specific interaction effect (Extended Data Fig. 4c). Thus, zlm fits the function: $Y\approx {\rm{CDR}}+{\rm{condition}}+{\rm{target}}+{\rm{condition}}\_{\rm{specific}}\_{\rm{interaction}}\_{\rm{effect}}$, where ${\rm{condition}}\_{\rm{specific}}\_{\rm{interaction}}\_{\rm{effect}}$ is a categorical variable indicating a cell that is both expressing the receptor (target^+^) and from a particular condition (that is, CIN^high^). The lrTest function evaluates the significance of including the ${\rm{condition}}\_{\rm{specific}}\_{\rm{interaction}}\_{\rm{effect}}$ covariate when modelling expression across all genes. The *P* values produced by this test are significant when the transcriptional response in receptor-expressing target cells differs across conditions (in this case, through perturbation of tumour CIN or *Sting1*), with condition-specific ligand availability. Again, we apply the Benjamini–Hochberg procedure to account for multiple hypothesis testing. Notably, the number of genes differentially expressed in receptor-expressing versus -null target cells is highly correlated across conditions, while those exhibiting an interaction effect (gene responses that differ in the presence of the ligand) are not (Extended Data Fig. 4d,e).

#### Defining ligand effects in target cells

Altogether, target and interaction tests were performed for all receptors and ligands in our database, crossed with all possible cell types in the TME. Target tests were performed within cells derived from the target cell type, conditioned on receptor expression; and interaction tests were performed in target cell types when their complementary ligand was differentially expressed across conditions in the TME. Thus, the output consists of *P* values and log_2_(FC) estimates across all genes for each component of a putative cell–cell interaction. To functionally define transcriptional responses to a ligand–receptor-mediated interaction, we compute $-{\log }_{10}\left({P}_{{\rm{adj}}}\right)\times {\log }_{2}({\rm{fold}}\;{\rm{change}})$ from the target likelihood ratio test for each gene, where ${P}_{{\rm{adj}}}$ is the Bonferroni-corrected *P* value. Ligand effects are then transcriptional response genes that exhibit a significant interaction effect in the presence of the condition-specific ligand.

For each cell type, we create a matrix of condition-specific transcriptional response vectors with rows corresponding to [receptor, target cell type] pairs and columns corresponding to all genes. Since each row of the matrix encodes both a cell type and a receptor, dependent transcriptional responses can be evaluated across multiple cell types. We then use scanpy to compute principal components on this matrix, choosing an optimal number of principal components for data dimensionality based on kneepoint analysis of the cumulative variance described by each component, and visualize in two dimensions with UMAP (Extended Data Fig. 4f). Phenotypic states associated with receptor expression in each cell type are computed according to $-{\log }_{10}\left({P}_{{\rm{adj}}}\right)\times {\log }_{2}({\rm{fold}}\;{\rm{change}})$ from the target likelihood ratio test for each gene, where ${P}_{{\rm{adj}}}$ is the Bonferroni-corrected *P* value. We compute principle components and the DC1 on this matrix using Palantir to identify genes that significantly correlate with this principle source of variance. After removing scores of zero and rescaling correlation values to the range [−1,1], we use these scores as input to gene set enrichment analysis (GSEA), along with cell-type-specific GMT files (provided in Supplementary Table 7), to assign pathways to these major axes of biological variation. For example, macrophage transcriptional responses largely reflected underlying single-cell heterogeneity in IFN-γ responsiveness and polarization (Fig. 4a).

#### Mapping ligand–receptor-mediated effects to cellular subpopulations

To assign ligand effects to subclusters within the target cell type, we took the dot-product between the transcriptional response score (defined above) and the log_2_(FC) of every gene in each cell subcluster versus all other cells using the MAST statistical framework^61^ (Extended Data Fig. 4g). The log_2_(FC) per gene per cluster is set to zero before computing this dot-product when it is not significant (FDR > 0.15). The dot-product score is standardized by normalizing to its max, and transcriptional response states (conditioned on receptor expression) are assigned to subclusters for standardized scores greater than 0.5; in this way, transcriptional response states can be assigned to more than one subcluster. Ligands are simply assigned to subclusters if they are positively enriched (FDR < 0.15, log_2_(FC) > 0) in that subcluster relative to all other cells in the donor cell type as determined by MAST^61^.

### Validating ligand effects predicted by ContactTracing

We downloaded the CytoSig database of human cytokine responses (https://cytosig.ccr.cancer.gov/download /, accessed 11 February 2022). This database provides measurements for 2,002 experiments in which cells were treated with a cytokine, and the log-fold expression change was measured across 19,918 genes. We mapped all genes in this database to mouse genes in our dataset, yielding a mapped database of 740 experiments with measurements in 13,013 genes. We then associated the ligands in the CytoSig database to their corresponding receptors in our set of mouse interactions, and focused on ligand–receptor pairs that are CIN-dependent (ligand log(FC) FDR < 0.05 and at least 1 significant interaction effect). We found 115 CIN-dependent ligand–receptor pairs, from 75 distinct receptors, that were in the mapped CytoSig database (in a total of 571 experiments across different cell types and conditions). We then compared every CIN-dependent transcriptional response measured by ContactTracing with each of the 571 cytokine responses measured by CytoSig. To compare the response vectors, we computed the connectivity score^62^, illustrated in Extended Data Fig. 5b, which is to test whether upregulated genes in one list are also upregulated in another, without making many assumptions about the distributions of values in the lists. ContactTracing upregulated genes have a log(FC) > 0 from the target test, and are CIN-specific (interaction test FDR < 0.05). We then apply the connectivity score to this set of cytokine response genes in CytoSig; the larger the score, the more these genes are also upregulated in CytoSig. We get a distribution of connectivity scores from our all-versus-all comparison. We then take a subset of these comparisons in which the target genes (receptor) are the same in each database, and the cell types are generally matched. There was a large variety of cell type names used in the CytoSig database; we manually created a mapping to ContactTracing cell types according to Supplementary Table 10 (many remain unmapped); we consider cell types ‘roughly matched’ if they both belong in one of the following sets: epithelial/stromal (tumour cells, fibroblast cells); myeloid (macrophages/myeloid-derived suppressor cells (mMDSC), plasmacytoid dendritic cells (pDC), classical dendritic cells (cDC), polymorphonuclear neutrophils (PMN)/granulocytic myeloid-derived suppressor cells (Gr-MDSC)) or lymphoid (T cells, B cells, NK cells). Extended Data Fig. 5c compares the distribution of all-versus-all connectivity scores, compared with the subset of those with matching target cell types and receptors. We used a Mann–Whitney test to determine that the connectivity scores are significantly higher in the matched subset (*P* = 0.0031).

### Benchmarking ContactTracing against existing methods that infer cell–cell interactions from single-cell data

To compare the top set of interactions predicted by ContactTracing with those predicted by other cell–cell interaction models, we evaluate their intersection (Fig. 3e and Extended Data Fig. 6a,b) and colocalization in matched spatial transcriptomics data (Fig. 3f and Extended Data Fig. 6c).

#### Implementation of alternative cell–cell interaction models

The expression counts and ligand–receptor databases used by ContactTracing were loaded using the typical workflows required by each respective tool. Counts matrices were split according to experimental condition. For all instances that required conversion between human and mouse gene names, we followed the same procedure described above (‘Mouse to human gene mapping’). Since some methods are unable to account for protein complex definitions, when necessary, complex interactions are split into all pairwise combinations of complex components to a given ligand/receptor. Common approaches to understanding ligand–receptor-mediated interactions are based on tests that compare co-expression of ligands and receptors across cell types. The most common example of such tests is CellPhoneDB^59^. As many methods are difficult to supply with custom ligand–receptor databases, we use the LIANA package (v.0.1.6)^63^, which reimplements many of these common methods. LIANA was configured to use the following methods: ‘cellphonedb’, ‘connectome’, ‘logfc’ (iTALK), ‘natmi’, ‘sca’ (SingleCellSignalR), ‘call_cellchat’ (CellChat) and ‘cytotalk’. Permutation-based tests were set to use 10,000 permutations, and CellChat was set to use 1,000 bootstraps. NicheNet^64^ v.1.1.0 was also implemented using a custom ‘ligand–receptor network’ with author-recommended settings, which allowed us to integrate the same database of complementary ligand–receptor pairs, while retaining the default ‘signalling’ and ‘gene regulatory’ networks. This new database was compiled using default optimized NicheNet hyperparameters. Since NicheNet is based on the Seurat toolkit, expression was preprocessed using a typical preprocessing workflow including its SCTransform ‘v2’ workflow (Seurat v.4.1.1, SCTransform v.0.3.3), with a consistent number of variable features as used for ContactTracing. NicheNet was run on all pairwise combinations of cell types with recommended parameters and ligand/receptor activity was scored using NicheNet’s Pearson correlation coefficients. A newer method for understanding cell–cell signalling is CellComm (part of the FUSCA package, v.1.3.1)^65^. Expression data were prepared for CellComm by following the typical FUSCA workflow demonstrated by the authors: counts were filtered to require a minimum of 100 genes expressed per cell and a minimum of 10 cells expressing each gene, then processed using the ‘Normalize’ and ‘scaleData’ functions. The CellComm algorithm was run by computing co-expression patterns with minimum mean expression set to 0.2, using 10,000 cluster permutations across cell types. CellComm *P* values were calculated using 1,000 permutations.

#### Application of cell–cell interaction models to human and mouse data

When running tools on spatially matched human TNBC and ER data^25^, we ran the typical workflow for each tool as described above on each condition independently so that each condition’s colocalization could be evaluated independently. To compare the results of ContactTracing with other tools in the mouse model of CIN, we ran LIANA-based methods on condition-specific counts matrices separately. As a substitute for the lack of condition-dependent analyses on those methods, we calculated a post hoc score for each method measuring the differential magnitude across conditions by computing the absolute value of the difference of CIN^high^ and CIN^low^ scores, and if *P* values were reported we selected the most significant value to be representative. These scores were then used to rank reported interactions from LIANA. To incorporate experimental conditions from the mouse model in NicheNet results, we used the full counts matrices (which includes both conditions) with the recently published ‘Differential NicheNet’ workflow, using ‘min_lfc’ specificity scores with an author-recommended cutoff of 0.15. While the typical Connectome scores are implemented in LIANA, the original implementation contains a ‘Differential Connectome’ workflow^66^ which would allow for explicit consideration of experimental conditions. Since it is also Seurat-based, we used the same data as prepared for Differential NicheNet and ran the method according to the author-recommended usage to analyse and calculate *P* values. While CellComm does not explicitly have a ‘Differential’ workflow, it has a ‘subcluster’ workflow which we used by setting experimental condition as the ‘cluster’ and cell-type annotation as the ‘subcluster’.

#### Comparing predicted interactions across models

As ContactTracing and alternative methods are run with a consistent ligand/receptor database, results differ only in terms of detection sensitivity and prioritization. Thus, interactions are compared in terms of set overlap (Fig. 3e and Extended Data Fig. 6a) and ranked differences (Extended Data Fig. 6b). For comparison, we required interactions to be present in both conditions and collapsed interactions to unique (target cell type, receptor) pairs. First, all methods that report a *P* value had results filtered using a 0.05 threshold. Next, for each target cell type/receptor pair, the maximum significant reported score (regardless of source cell type and ligand) was selected to be the representative score for each target cell type/receptor pair. Rankings were then determined by sorting target cell type/receptor pairs according to previously described maximum scores. Similarly, ContactTracing target cell type/receptor pairs were first filtered by requiring at least one significant interaction term (FDR < 0.05) in the target cell type for ligands that were differentially expressed across conditions in any donor cell type (absolute log_2_(FC) > 0 and FDR < 0.05). The ContactTracing target cell type/receptor pairs were then sorted by the number of significant interaction terms, with ties broken by secondarily sorting according to the number of DEGs for a given target cell type/receptor pair. Since the methods had variable numbers of results reported, overlap coefficients were calculated to represent set similarity. The overlap coefficient is a set size-invariant metric for similarity that is related to the Jaccard index. While the Jaccard index for sets *X* and *Y* is calculated as ${\rm{Jaccard}}=\frac{| \,X\cap Y\,| }{| \,X\cup Y\,| }$, the overlap coefficient corrects for set size difference by normalizing set intersection cardinality by minimum set cardinality rather than the cardinality of the union between sets, that is, ${\rm{Overlap}}=\frac{| \,X\cap Y\,| }{\min (| \,X\,| ,| \,Y\,| )}$. Similarly to the Jaccard index, overlap coefficients range from 0 to 1, where 1 represents the highest degree of overlap. All pairwise combinations of ranked result lists were then used to calculate corresponding overlap coefficients for various rank thresholds.

### Validation within an independent human breast cancer cohort

To validate the relevance of key biological findings in human breast cancers, we obtained scRNA-seq data from a publicly available cohort of 26 primary breast cancer tumours (11 ER^+^, 5 HER2^+^ and 10 TNBCs)^25^. To compare cell subtypes between the human and mouse cell atlases, we mapped the subtype annotations provided by Wu et. al.^25^ to the most similar cell subtype in the mouse for all immune cells where a corresponding cell subtype was present (Supplementary Table 10). This was done using subtype-specific DEGs and pathways provided by the original authors and recomputed using our pipeline. Most original DEGs and annotations published were validated by our analyses, except for the Myeloid:c8 S100A9^+^ cluster, which we classify as mMDSCs based on their upregulation of S100A8 and S100A9 (ref. ^67^). Following the detection of significant sample-specific effects, Harmony^68^ was applied for batch correction to the full log-transformed count matrix to generate the default *n* = 100 corrected Harmony principal components. Using the optimal number of principal components selected before and after batch correction (*n* = 17 and *n* = 19, respectively), sample mixing was noticeably improved in immune cell subsets; thus, corrected Harmony principal components were used for downstream differential abundance testing (Extended Data Fig. 12c,d). To validate CIN-dependent findings from the 4T1 mouse model, we focused on the eight TNBC samples that had tumour cells present in the data. To separate these eight samples into expected ‘CIN^low^’ and ‘CIN^high^’ groups, we used the standard inferCNV i6 HMM model^69^ to detect copy number variants (CNVs) within the tumour cell compartment for each sample (applied to raw data). As a measure of CIN, we computed the Shannon diversity index of the variant states, weighted by the number of copy number alterations in each variant, for all tumour cells in each sample:

$${\rm{CN}}{{\rm{V}}}_{{\rm{SDI}}}=\mathop{\sum }\limits_{i=1}^{n}-\,{s}_{i}\times {\rm{ln}}({s}_{i})$$

where *n* is the number of unique predicted variants in current sample

$${s}_{i}=\frac{{\rm{fre}}{{\rm{q}}}_{i}\times {\delta }_{i}}{{\sum }_{i=1}^{n}{\rm{fre}}{{\rm{q}}}_{i}\times {\delta }_{i}}$$

where freq_*i*_ is the proportion of variant *i* in current sample and *δ*_*i*_ is the sum of the absolute values of the predicted difference from normal across all chromosome positions for variant *i*. This CNV_SDI_ metric not only captures the diversity in the unique CNV states detected in the sample, but it also accounts for how altered these states are predicted to be from diploid. As expected, the CNV_SDI_ was markedly higher in CIN^high^ mouse samples (Extended Data Fig. 1l) and was used in conjunction with the mean tumour cell expression of key pathways (Type 1 IFN, CIN signature, Non-Canonical Nf-Kb and Hallmark UPR) to cluster the eight human TNBC samples into CIN^low^ (*n* = 4) and CIN^high^ (*n* = 4) subsets (Extended Data Fig. 12a). We then used the Milo^70^ python framework to compute differentially abundant neighbourhoods within the TNBC subset between the inferred CIN^low^ and CIN^high^ samples (*k* = 15, *P* = 0.5 and *d* = 22). The mapped cell subtype annotations were used to label each neighbourhood based on the mode cell subtype and log_2_(FC) values were mapped to the single-cell resolution in the same manner as described in Supplementary Note 5. ContactTracing was likewise applied to these human data to detect CIN-specific ligand effects, as described in the section above (‘ContactTracing to identify and map the effects of conditionally dependent cell–cell interactions’).

The breast cancer dataset also includes matched Visium spatial transcriptomics data from four of the samples: two patients with TNBC and two ER^+^ patients. We ran ContactTracing on the scRNA-seq data for these samples separately, comparing TNBC versus ER^+^ conditions. We used the output from ContactTracing to rank interactions relevant to each condition; interactions identified by [ligand, receptor, receptor cell type] are ranked by the number of significant interaction effects, multiplied by the identity function that indicates whether the ligand is upregulated in at least one cell type for the relevant condition. Therefore, there is a different ranking of interactions relevant to TNBC, and of those relevant to ER^+^. For each of the four patients, we then used the relevant ranking, and assessed whether top TNBC or ER^+^ interactions tended to colocalize in the spatial data for patients in corresponding breast cancer subtypes (Fig. 3f). Colocalization was determined by summing the product of [log(ligand expression), probability or Pr(target cell type), Identity(receptor expressed)] across all cells in the spatial data for an individual. Ligand expression was then permuted 100 times and the colocalization statistic recomputed to obtain a colocalization *P* value (Extended Data Fig. 6c). The probability of a target cell type in each Visium spot was determined using the deconvolution software SPOTlight^71^. The SPOTlight algorithm was seeded with scRNA-seq data from the same individuals, and the cell-type annotations described in the previous section.

### Data visualization

#### Two-dimensional embeddings

The global atlas of all cells in the TME, including diverse tumour, stroma, lymphoid and myeloid subsets, was visualized using a UMAP (Fig. 2a). This dimensionality reduction technique was appropriate given the diversity of cell types represented. Force-directed graphs^72^ were alternatively used to visualize continuous subpopulations within major cell types (Extended Data Figs. 2d, 3a and 7b,e), because these better capture cell state transitions and local relationships between cells. For both visualization methods, we used the optimal number of principal components and the default *k* = 15 nearest neighbours with scanpy.

#### Gene expression along within-cell-type trajectories

Heatmaps were generated using the CellRank^73^ heatmap plotting function, which uses a generalized additive model to smooth expression along the given trajectory. Imputed expression was used to generate these visuals and expression was normalized to a range of [0,1]. The order of genes was determined by expression peak along the trajectory. Transition genes did not exhibit an expression peak at either end point of the inferred continuum. Colour bars above heatmaps were generated by ranking the given variable along the given trajectory; continuous variables were smoothed using the CellRank methodology. Similarly, gene trend curves were generated using the built-in plotting method provided by CellRank using the same generalized additive model method as in the heatmap visual described above. Here, imputed expression was normalized to its max for each gene independently.

#### Neighbourhood differential abundance plots

After mapping cell subtypes to Milo neighbourhoods (Supplementary Note 5), differential abundance test results were visualized per neighbourhood using strip plots overlaid on mean bar plots for significantly differentially abundant neighbourhoods. In minority cell-type populations where fewer than two significant neighbourhoods were detected, all neighbourhoods were used for computing the log_2_(FC) mean. The size (or opacity) of the scatter points reflects the significance (*P* value) of the neighbourhoods, log_2_(FC) (Fig. 2b and Extended Data Figs. 2c,i, 3c,g, 7f and 12c,d).

### Statistics and reproducibility

Experiments showing representative images were independently repeated two (Fig. 5a,e, Extended Data Fig. 11a,d and Supplementary Note 6) or three (Extended Data Figs. 1a,e,f, 10a and 11c) times with similar results.

### Reporting summary

Further information on research design is available in the Nature Portfolio Reporting Summary linked to this article.

## Online content

Any methods, additional references, Nature Portfolio reporting summaries, source data, extended data, supplementary information, acknowledgements, peer review information; details of author contributions and competing interests; and statements of data and code availability are available at 10.1038/s41586-023-06464-z.

## Supplementary information

Supplementary Information This file contains Supplementary Figs. 1–3, Notes 1–6, Tables 1–5 and references.

Reporting Summary

Supplementary Table 6 Cell type marker file used for cellAssign.

Supplementary Table 7 Cell-type-specific GMT files for gene set enrichment.

Supplementary Table 8 Differentially expressed genes and pathways across cell subtypes and along cell trajectories for major cell types in Fig. 2a.

Supplementary Table 9 Data underlying the Circos plots in Fig. 4a and Extended Data Fig. 12e.

Supplementary Table 10 Cell type mappings between CytoSig and ContactTracing cell types in the Validating ligand effects predicted by ContactTracing section, and human and mouse cell types in the Validation within an independent human breast cancer cohort section.

## Data Availability

All scRNA-seq data generated in this study have been deposited in the NCBI’s Gene Expression Omnibus (GEO) database under accession code: GSE189856. The GRCm38 genome reference is available as a CellRanger reference package (v.mm10-3.0.0). All scRNA-seq data from the independent human cohort are available in the NCBI’s GEO under accession code: GSE176078, and the spatial data from the same study are at https://zenodo.org/record/4739739. CellPhoneDb can be found at https://www.cellphonedb.org (v.2.1.4 was used for this study), and the celltalkdb database is at http://tcm.zju.edu.cn/celltalkdb/download.php. An interactive web dashboard is made available at http://contacttracing.laughneylab.com to enable interactive exploration of data from this study, allowing users to visualize pairwise ligand–receptor-mediated interactions and systems-level interactions in Circos plots (similar to Fig. 4a and Extended Data Fig. 12e) using plotly v.5.11.0 and dash v.2.7.1. Processed scRNA-seq datasets appropriate for input to the ContactTracing method are available at 10.5281/zenodo.8061222. Source data are provided with this paper.

## Source data

Source Data Fig. 1
Source Data Fig. 5
Source Data Extended Data Fig. 1
Source Data Extended Data Fig. 2
Source Data Extended Data Fig. 8
Source Data Extended Data Fig. 9
Source Data Extended Data Fig. 11

## Code availability

Custom code, including docker environments with jupyter notebooks demonstrating the ContactTracing method, is available on the Laughney Lab GitHub (https://github.com/LaughneyLab/ContactTracing_tutorial, https://zenodo.org/record/8061480). Code for the interactive web dashboard is available on GitHub (https://github.com/LaughneyLab/ContactTracing-Viz/, https://zenodo.org/record/8067675). Circos software^74^ (v.0.69-9) was used to visualize ligand–receptor interactions based on measured transcriptional responses (ContactTracing). The network plot in Extended Data Fig. 4h was created with Cytoscape^75^ v.3.8.2.

## References

[1] Li, J. et al. Metastasis and immune evasion from extracellular cGAMP hydrolysis. *Cancer Discov.* **11**, 1212–1227 (2021). 10.1158/2159-8290.CD-20-0387 PMC8102348 33372007

[2] Bakhoum, S. F. et al. Chromosomal instability drives metastasis through a cytosolic DNA response. *Nature* **553**, 467–472 (2018). 10.1038/nature25432 PMC5785464 29342134

[3] Bakhoum SF, Cantley LC (2018). The multifaceted role of chromosomal instability in cancer and its microenvironment. Cell.

[4] Wormann SM (2021). APOBEC3A drives deaminase domain-independent chromosomal instability to promote pancreatic cancer metastasis. Nat. Cancer.

[5] Lengauer C, Kinzler KW, Vogelstein B (1997). Genetic instability in colorectal cancers. Nature.

[6] Lee AJ (2011). Chromosomal instability confers intrinsic multidrug resistance. Cancer Res..

[7] Taylor AM (2018). Genomic and functional approaches to understanding cancer aneuploidy. Cancer Cell.

[8] Davoli, T., Uno, H., Wooten, E. C. & Elledge, S. J. Tumor aneuploidy correlates with markers of immune evasion and with reduced response to immunotherapy. *Science*10.1126/science.aaf8399 (2017). 10.1126/science.aaf8399 PMC5592794 28104840

[9] Bakhoum SF (2014). The mitotic origin of chromosomal instability. Curr. Biol..

[10] Thompson SL, Compton DA (2008). Examining the link between chromosomal instability and aneuploidy in human cells. J. Cell Biol..

[11] Santaguida S (2017). Chromosome mis-segregation generates cell-cycle-arrested cells with complex karyotypes that are eliminated by the immune system. Dev. Cell.

[12] Laucius CD, Orr B, Compton DA (2019). Chromosomal instability suppresses the growth of K-Ras-induced lung adenomas. Cell Cycle.

[13] Hoevenaar WHM (2020). Degree and site of chromosomal instability define its oncogenic potential. Nat. Commun..

[14] Nguyen B (2022). Genomic characterization of metastatic patterns from prospective clinical sequencing of 25,000 patients. Cell.

[15] Watkins, T. B. K. et al. Pervasive chromosomal instability and karyotype order in tumour evolution. *Nature*10.1038/s41586-020-2698-6 (2020). 10.1038/s41586-020-2698-6 PMC7611706 32879494

[16] Bakhoum SF, Thompson SL, Manning AL, Compton DA (2009). Genome stability is ensured by temporal control of kinetochore-microtubule dynamics. Nat. Cell Biol..

[17] Hatch EM, Fischer AH, Deerinck TJ, Hetzer MW (2013). Catastrophic nuclear envelope collapse in cancer cell micronuclei. Cell.

[18] Mackenzie KJ (2017). cGAS surveillance of micronuclei links genome instability to innate immunity. Nature.

[19] Harding SM (2017). Mitotic progression following DNA damage enables pattern recognition within micronuclei. Nature.

[20] Ablasser, A. & Chen, Z. J. cGAS in action: expanding roles in immunity and inflammation. *Science*10.1126/science.aat8657 (2019). 10.1126/science.aat8657 30846571

[21] Moore AT (2005). MCAK associates with the tips of polymerizing microtubules. J. Cell Biol..

[22] Liu H (2018). Nuclear cGAS suppresses DNA repair and promotes tumorigenesis. Nature.

[23] Jiang P (2021). Systematic investigation of cytokine signaling activity at the tissue and single-cell levels. Nat. Methods.

[24] Bartneck M (2019). The CCR2^+^ macrophage subset promotes pathogenic angiogenesis for tumor vascularization in fibrotic livers. Cell. Mol. Gastroenterol. Hepatol..

[25] Wu SZ (2021). A single-cell and spatially resolved atlas of human breast cancers. Nat. Genet..

[26] Dhanda J (2014). SERPINE1 and SMA expression at the invasive front predict extracapsular spread and survival in oral squamous cell carcinoma. Br. J. Cancer.

[27] Jiang S (2021). Activation of WNT7b autocrine eases metastasis of colorectal cancer via epithelial to mesenchymal transition and predicts poor prognosis. BMC Cancer.

[28] Acharyya S (2012). A CXCL1 paracrine network links cancer chemoresistance and metastasis. Cell.

[29] Lim SY, Yuzhalin AE, Gordon-Weeks AN, Muschel RJ (2016). Targeting the CCL2-CCR2 signaling axis in cancer metastasis. Oncotarget.

[30] Johnstone CN, Chand A, Putoczki TL, Ernst M (2015). Emerging roles for IL-11 signaling in cancer development and progression: focus on breast cancer. Cytokine Growth Factor Rev..

[31] Hong C (2022). cGAS-STING drives the IL-6-dependent survival of chromosomally instable cancers. Nature.

[32] Adamson B (2016). A multiplexed single-cell CRISPR screening platform enables systematic dissection of the unfolded protein response. Cell.

[33] Haag SM (2018). Targeting STING with covalent small-molecule inhibitors. Nature.

[34] Dou Z (2017). Cytoplasmic chromatin triggers inflammation in senescence and cancer. Nature.

[35] Gui X (2019). Autophagy induction via STING trafficking is a primordial function of the cGAS pathway. Nature.

[36] Ishikawa H, Ma Z, Barber GN (2009). STING regulates intracellular DNA-mediated, type I interferon-dependent innate immunity. Nature.

[37] Wang RW, Vigano S, Ben-David U, Amon A, Santaguida S (2021). Aneuploid senescent cells activate NF-κB to promote their immune clearance by NK cells. EMBO Rep..

[38] Wang H (2017). cGAS is essential for the antitumor effect of immune checkpoint blockade. Proc. Natl Acad. Sci. USA.

[39] Ranoa DRE (2019). STING promotes homeostasis via regulation of cell proliferation and chromosomal stability. Cancer Res..

[40] Nassour J (2019). Autophagic cell death restricts chromosomal instability during replicative crisis. Nature.

[41] Hu, J. et al. STING inhibits the reactivation of dormant metastasis in lung adenocarcinoma. *Nature*10.1038/s41586-023-05880-5 (2023). 10.1038/s41586-023-05880-5 PMC10569211 36991128

[42] Rosenthal R (2019). Neoantigen-directed immune escape in lung cancer evolution. Nature.

[43] Fujiwara T (2005). Cytokinesis failure generating tetraploids promotes tumorigenesis in p53-null cells. Nature.

[44] Ahn J (2014). Inflammation-driven carcinogenesis is mediated through STING. Nat. Commun..

[45] Lemos H (2016). STING promotes the growth of tumors characterized by low antigenicity via IDO activation. Cancer Res..

[46] Foijer F (2014). Chromosome instability induced by Mps1 and p53 mutation generates aggressive lymphomas exhibiting aneuploidy-induced stress. Proc. Natl Acad. Sci. USA.

[47] Foijer, F. et al. Deletion of the MAD2L1 spindle assembly checkpoint gene is tolerated in mouse models of acute T-cell lymphoma and hepatocellular carcinoma. *eLife*10.7554/eLife.20873 (2017). 10.7554/eLife.20873 PMC5400506 28318489

[48] Shoshani O (2021). Transient genomic instability drives tumorigenesis through accelerated clonal evolution. Genes Dev..

[49] Frittoli, E. et al. Tissue fluidification promotes a cGAS-STING cytosolic DNA response in invasive breast cancer. *Nat. Mater.*10.1038/s41563-022-01431-x (2022). 10.1038/s41563-022-01431-x PMC10156599 36581770

[50] Meric-Bernstam F (2022). Phase I dose-escalation trial of MIW815 (ADU-S100), an intratumoral STING agonist, in patients with advanced/metastatic solid tumors or lymphomas. Clin. Cancer Res..

[51] Meric-Bernstam F (2023). Combination of the STING agonist MIW815 (ADU-S100) and PD-1 inhibitor spartalizumab in advanced/metastatic solid tumors or lymphomas: an open-label, multicenter, phase Ib study. Clin. Cancer Res..

[52] Lama L (2019). Development of human cGAS-specific small-molecule inhibitors for repression of dsDNA-triggered interferon expression. Nat. Commun..

[53] Calvo V (2021). Discovery of 2-amino-3-amido-5-aryl-pyridines as highly potent, orally bioavailable, and efficacious PERK kinase inhibitors. Bioorg. Med. Chem. Lett..

[54] Duits DEM, Wellenstein MD, de Visser KE (2020). In vitro assessment of cancer cell-induced polarization of macrophages. Methods Enzymol..

[55] Tozbikian G (2014). Mesothelin expression in triple negative breast carcinomas correlates significantly with basal-like phenotype, distant metastases and decreased survival. PLoS ONE.

[56] Yarilin D (2015). Machine-based method for multiplex in situ molecular characterization of tissues by immunofluorescence detection. Sci. Rep..

[57] Lawrence M (2013). Software for computing and annotating genomic ranges. PLoS Comput. Biol..

[58] Love MI, Huber W, Anders S (2014). Moderated estimation of fold change and dispersion for RNA-seq data with DESeq2. Genome Biol..

[59] Efremova M, Vento-Tormo M, Teichmann SA, Vento-Tormo R (2020). CellPhoneDB: inferring cell-cell communication from combined expression of multi-subunit ligand-receptor complexes. Nat. Protoc..

[60] Shao, X. et al. CellTalkDB: a manually curated database of ligand-receptor interactions in humans and mice. *Brief. Bioinform.*10.1093/bib/bbaa269 (2021). 10.1093/bib/bbaa269 33147626

[61] Finak G (2015). MAST: a flexible statistical framework for assessing transcriptional changes and characterizing heterogeneity in single-cell RNA sequencing data. Genome Biol..

[62] Musa A (2017). A review of connectivity map and computational approaches in pharmacogenomics. Brief. Bioinform..

[63] Dimitrov D (2022). Comparison of methods and resources for cell-cell communication inference from single-cell RNA-Seq data. Nat. Commun..

[64] Browaeys R, Saelens W, Saeys Y (2020). NicheNet: modeling intercellular communication by linking ligands to target genes. Nat. Methods.

[65] Lummertz da Rocha E (2022). CellComm infers cellular crosstalk that drives haematopoietic stem and progenitor cell development. Nat. Cell Biol..

[66] Guilliams M (2022). Spatial proteogenomics reveals distinct and evolutionarily conserved hepatic macrophage niches. Cell.

[67] Veglia F, Sanseviero E, Gabrilovich DI (2021). Myeloid-derived suppressor cells in the era of increasing myeloid cell diversity. Nat. Rev. Immunol..

[68] Korsunsky I (2019). Fast, sensitive and accurate integration of single-cell data with Harmony. Nat. Methods.

[69] Tickle, T., Tirosh, I., Georgescu, C., Brown, M. & Haas, B. inferCNV of the Trinity CTAT Project. Klarman Cell Observatory, Broad Institute of MIT and Harvard, Cambridge, MA, USA (2019).

[70] Dann, E., Henderson, N. C., Teichmann, S. A., Morgan, M. D. & Marioni, J. C. Differential abundance testing on single-cell data using *k*-nearest neighbor graphs. *Nat. Biotechnol.*10.1038/s41587-021-01033-z (2021). 10.1038/s41587-021-01033-z PMC7617075 34594043

[71] Elosua-Bayes M, Nieto P, Mereu E, Gut I, Heyn H (2021). SPOTlight: seeded NMF regression to deconvolute spatial transcriptomics spots with single-cell transcriptomes. Nucleic Acids Res..

[72] Jacomy M, Venturini T, Heymann S, Bastian M (2014). ForceAtlas2, a continuous graph layout algorithm for handy network visualization designed for the Gephi software. PLoS ONE.

[73] Lange, M. et al. CellRank for directed single-cell fate mapping. *Nat. Methods* **19**, 159–170 (2022). 10.1038/s41592-021-01346-6 PMC8828480 35027767

[74] Krzywinski M (2009). Circos: an information aesthetic for comparative genomics. Genome Res..

[75] Shannon P (2003). Cytoscape: a software environment for integrated models of biomolecular interaction networks. Genome Res..

