# Supplementary material for: Non-cell-autonomous cancer progression from chromosomal instability
Source: Nature. 2023 Aug 23;620(7976):1080–8. doi: 10.1038/s41586-023-06464-z (PMC10468402; doi:10.1038/s41586-023-06464-z)
Supplement: Supplementary file 1 — This file contains Supplementary Figs. 1–3, Notes 1–6, Tables 1–5 and references. [file 41586_2023_6464_MOESM1_ESM.pdf]

nature portfolio

https://doi.org/10.1038/s41586-023-06464-z

Supplementary information

Non-cell-autonomous cancer progression from chromosomal instability

In the format provided by the authors and unedited

Supplementary Information

Non-cell-autonomous cancer progression from chromosomal instability

Jun Li[1,2,*], Melissa J. Hubisz[3,4,5,6*], Ethan M. Earlie[3,4,5*], Mercedes A. Duran[1,2,*], Christy Hong[1,2], Austin A. Varela[3,4,5], Emanuele Lettera[1,2], Matthew Deyell[3,4,5], Bernardo Tavora[7], Jonathan J. Havel[7], Su M Phyu[8], Amit Dipak Amin[9,10], Karolina Budre[3,4,5], Erina Kamiya[3,4,5], Julie-Ann Cavallo[1,2], Christopher Garris[11,12], Simon Powell[2], Jorge S. Reis-Filho[13], Hannah Wen[13], Sarah Bettigole[7], Atif J. Khan[2], Benjamin Izar[9,10], Eileen E. Parkes[8], Ashley M. Laughney[3,4,5,$,#], Samuel F. Bakhoum[1,2,$,#]

[1] Human Oncology and Pathogenesis Program, Memorial Sloan Kettering Cancer Center, New York, NY

[2] Department of Radiation Oncology, Memorial Sloan Kettering Cancer Center, New York, NY

[3] Department of Physiology, Biophysics, and Systems Biology, Weill Cornell Medicine, New York, NY

[4] Meyer Cancer Center, Weill Cornell Medicine, New York, NY

[5] Institute for Computational Biomedicine, Weill Cornell Medicine, New York, NY

[6] Bioinformatics Facility, Institute of Biotechnology, Cornell University, Ithaca, NY

[7] Volastra Therapeutics Inc., New York, NY

[8] Department of Oncology, Medical Sciences Division, University of Oxford, Oxford, UK

[9] Columbia Center for Translational Immunology, New York, NY

[10] Columbia University Medical Center, Division of Hematology and Oncology, New York, NY

[11] Department of Pathology, Harvard Medical School, Boston, MA

[12] Center for Systems Biology, Massachusetts General Hospital, Boston, MA

[13] Department of Pathology and Laboratory Medicine, Memorial Sloan Kettering Cancer Center, New York, NY

[*] These authors contributed equally to this work

[$] These authors contributed equally to and co-lead this work

Co-corresponding and co-senior authors:
Samuel F. Bakhoum, M.D., Ph.D.
Human Oncology and Pathogenesis Program
Department of Radiation Oncology
Memorial Sloan Kettering Cancer Center
New York, N.Y., 10065
Email: samuel.bakhoum@gmail.com
Phone: 212-639-5749

Ashley M. Laughney, Ph.D.
Department of Physiology and Biophysics
Institute for Computational Biomedicine
Weill Cornell Medicine
New York, N.Y., 10021
Email: ashley.laughney@gmail.com
Phone: 646-962-2739

Table of Contents

**Contents**	**Page Number**
Table of Contents	1
Supplementary Figure 1	2
Supplementary Figure 2	4
Supplementary Figure 3	4
Supplementary Note 1	5
Supplementary Note 2	7
Supplementary Note 3	7
Supplementary Note 4	10
Supplementary Note 5	13
Supplementary Note 6	14
Supplementary Table 1	16
Supplementary Table 2	16
Supplementary Table 3	17
Supplementary Table 4	17
Supplementary Table 5	17
References	19

Supplementary Figure 1

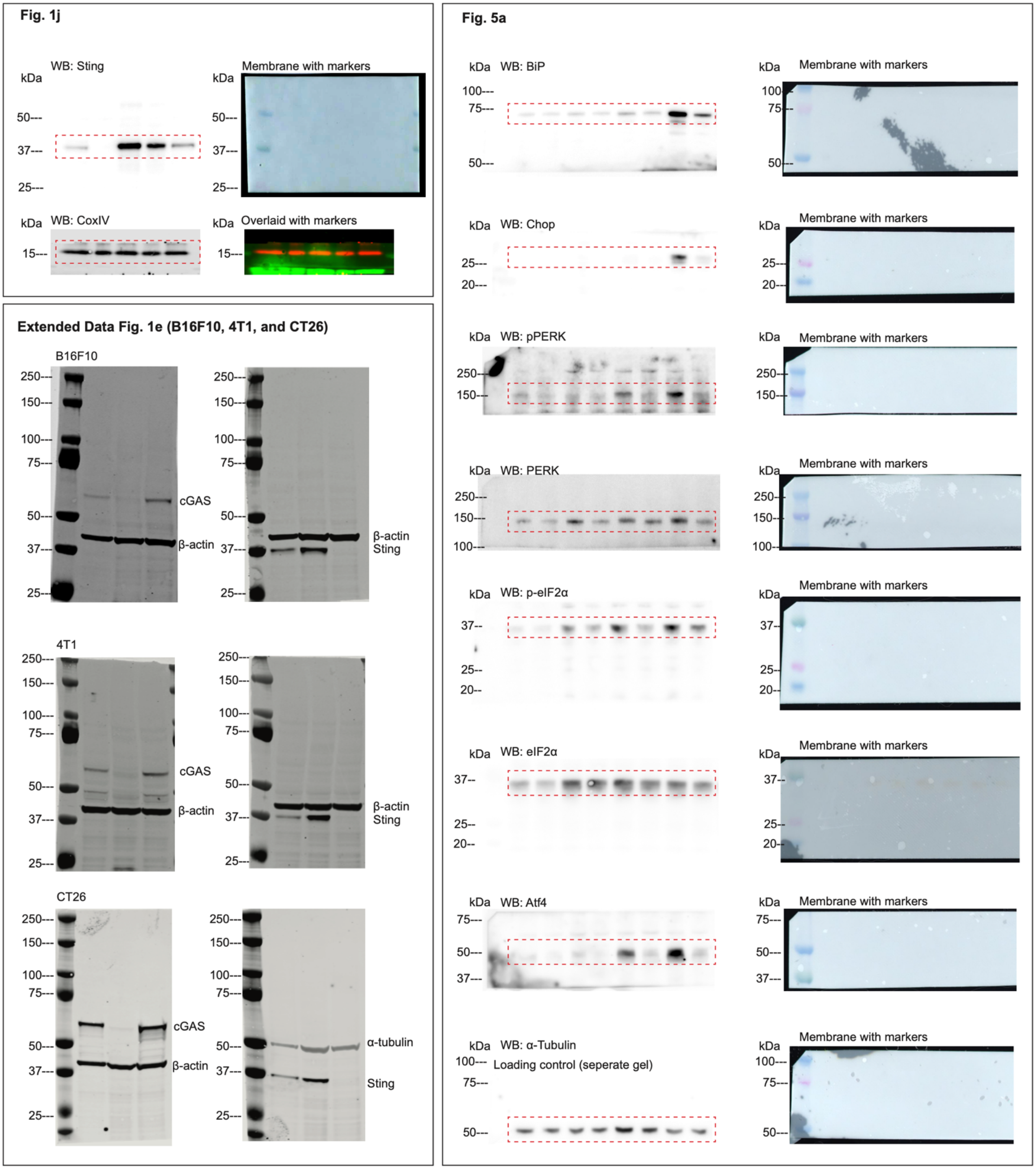

Supplementary Figure 1 (continued)

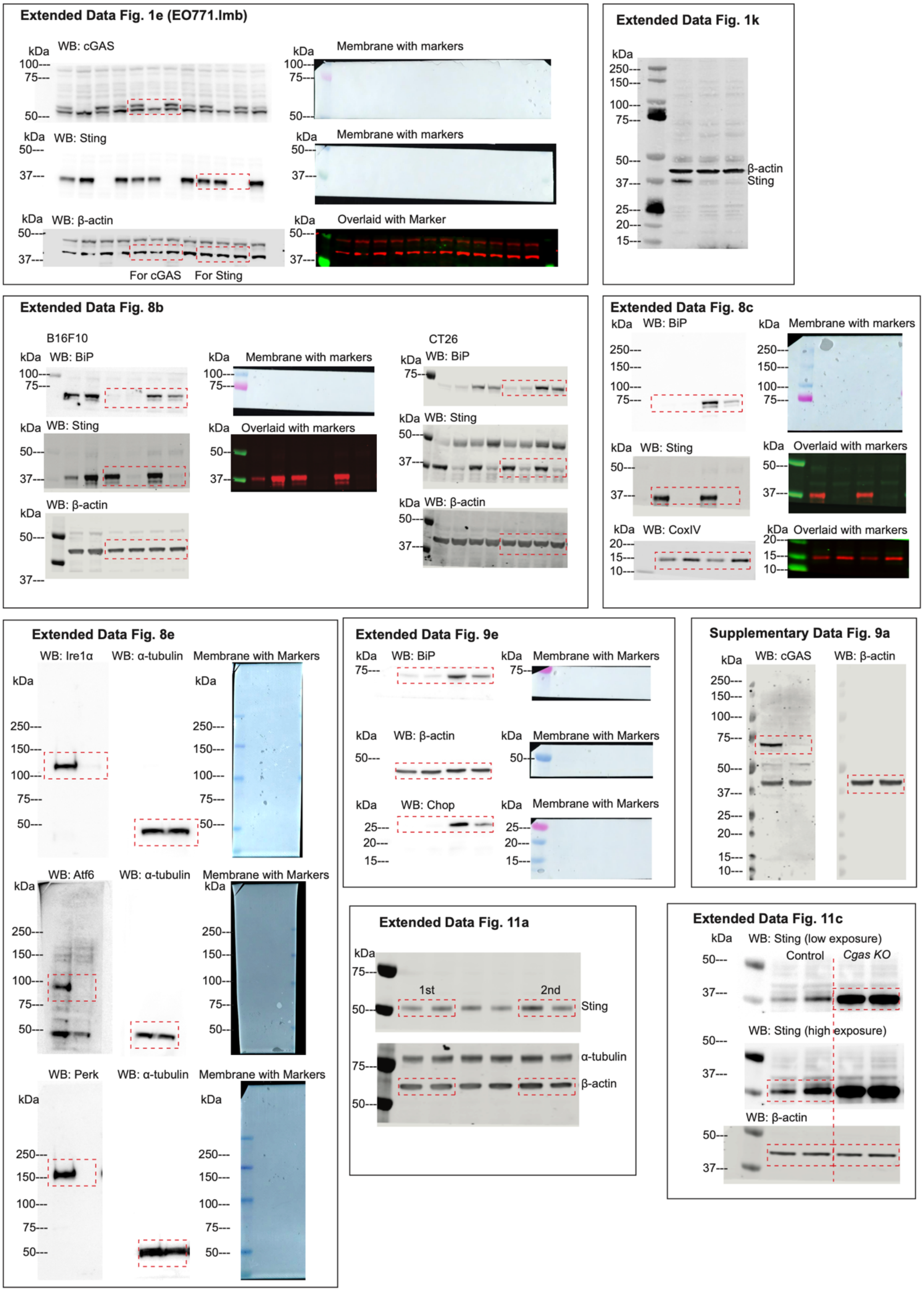

Supplementary Figure 1. Raw, unprocessed images of western blots reported in this study.

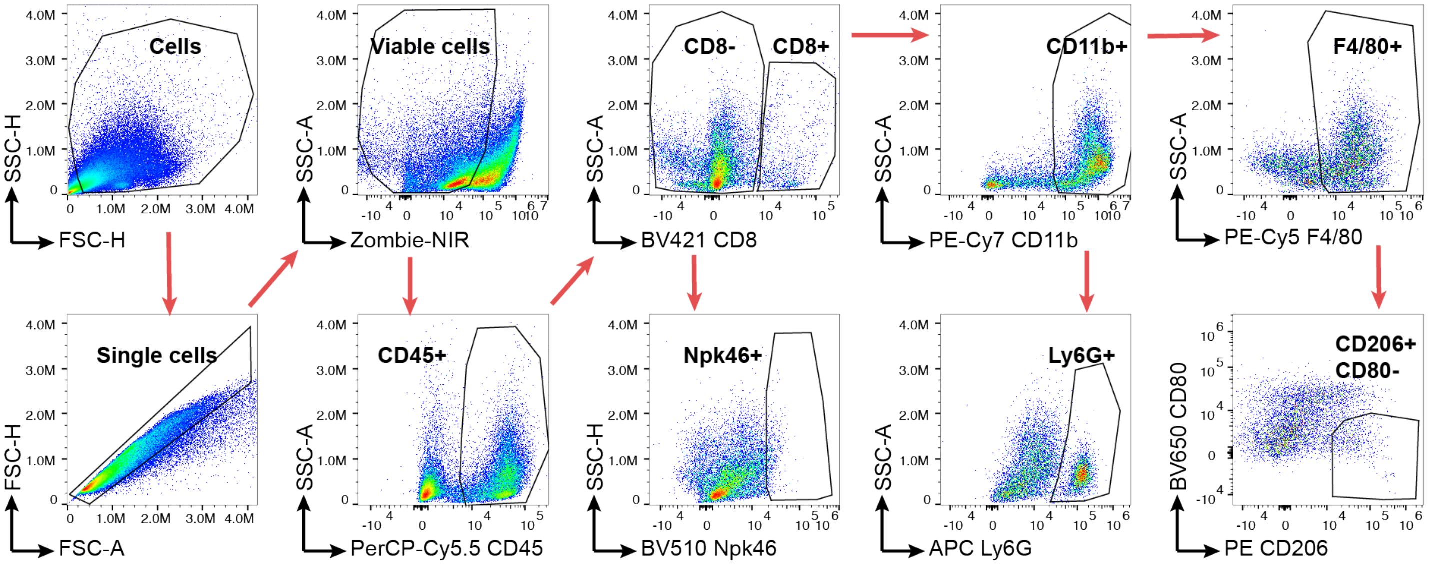

Supplementary Figure 2. Gating strategy used in flow cytometry analysis.

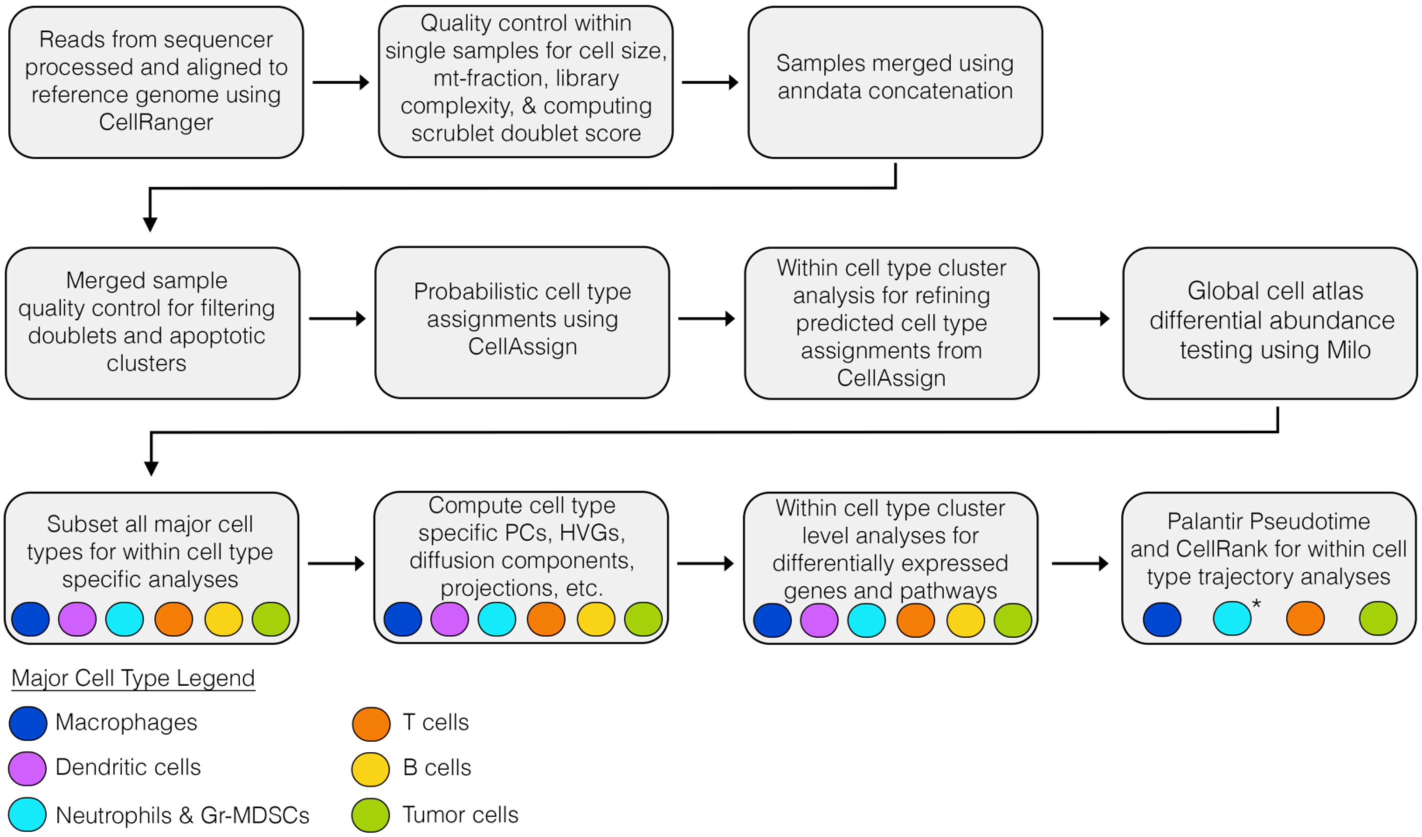

Supplementary Figure 3. Single cell workflow diagram. Graphical illustration of the single cell workflow outlined in Supplementary Notes 1-5 below. Major cell types used for within-cell type analyses are denoted by color dots. Astrix in "Palantir Pseudotime and CellRank" block indicates full trajectory analyses was not run on the granulocytic cell type.

Supplementary Note 1 | Single cell RNA-seq data pre-processing.

Pre-processing, cell selection, and filtering of individual scRNA-seq samples. CellRanger (v3.1.0) was utilized to construct a count matrix from raw reads, including sample demultiplexing, alignment to CellRanger's mm10-3.0.0 reference, barcode processing, and the generation of a raw digital expression matrix by collapsing groups of reads with the same unique molecular identifier (UMI), cell barcode and gene annotation. The count matrix was then loaded into python using scanpy[1] (*read_10x_h5*) for subsequent pre-processing and downstream analysis. Redundant gene instances (n = 35) were collapsed by summing their cumulative molecule counts. We then followed several filtering steps within each sample to ensure high data quality. Viable cells were distinguished from droplets consisting of ambient mRNA transcripts arising in solution due to premature lysis or cell death based on library size. As illustrated in **Supplementary Fig. 4a**, cells were ranked by library size (total molecule counts) in descending order. We then computed the first and second derivative of the normalized sum of this array (based on average of a 10-cell rolling window) and identified the inflection point, or first instance in which the second derivative is zero. All cells with a library size less than 0.9X the inflection point were discarded. Additionally, cells with > 10% of transcripts derived from mitochondria (**Supplementary Fig. 4b**), cells with low coverage defined by:

$$\frac{reads}{molecule} < \mu\left(\frac{reads}{molecule}\right) - 2.5 \times std\left(\frac{reads}{molecule}\right),$$

or cells with low complexity libraries (in which detected molecules align to a small subset of genes determined by at least 0.4X standard deviations from a linear fit) (**Supplementary Fig. 4c**) were discarded. Within individual samples, scrublet[2] was used to predict cell doublet scores based on simulated multiplets from the dataset using a prior expected double rate of 0.06 (based on targeted cell loading concentration). The filtered count matrix was normalized for library size per cell, whereby the expression level of each gene was divided by the cell's total library size and then scaled by a constant of 10,000. Finally, the normalized count matrix was log-transformed according to: $\log(x) = log_{10}(x + 0.1) - log_{10}(0.1)$, to generate the log-transformed count matrix.

Merged sample library generation. Following single sample pre-processing and filtering, all biological samples (n = 13 murine tumors) were merged to yield a total of 41,376 high-quality cells with a median library size of 2,439 transcripts per cell. Cell size, viability and complexity metrics were of high quality and largely consistent across samples (**Supplementary Fig. 4d**). Genes detected in fewer than 10 cells (n = 14,413) were excluded; highly variable genes (HVGs) were then computed per sample using the CellRanger flavor of scanpy[1]. Cell doublet scores computed in individual samples were subsequently assessed at the cluster and single cell level for the merged library. Towards the former, Principal Component Analysis (PCA) was applied to the log-transformed count matrix. Phenograph clustering[3] was computed on the top 50 principal components (*k-nearest neighbors* = 20) and the average doublet score was characterized per cluster. Three Phenograph clusters distinguished by high average double score and individual cells with doublet scores greater than a threshold (point where the first derivative of the doublet score distribution, smoothed using the Savitzky-Golay filter from SciPy[4], plateaus to zero) were removed. Altogether, this resulted in the removal of 474 putative doublet cells from the merged cell atlas.

Evaluating sample-specific batch effects. Library batch effects were ruled out by quantifying local sample mixing in non-tumor CD45+ cells before and after applying a fast mutual nearest neighbor (MNN) batch-correction[5]. Cell states were highly reproducible within each condition, showing similar levels of sample mixing within the non-tumor, CD45+ fraction before and after applying a fast mutual nearest neighbor (MNN) batch-correction (**Supplementary Fig. 4e**). Some sample-specific biological variance is expected across tumor cell states[6,7]. Sample mixing within each condition (CIN^{high}, CIN^{low}, CIN^{high}+*Sting1*^{KD}) was computed for a given cell, within a local neighborhood (30-nearest neighbors) according to the Shannon entropy:

$$entropy_i = -\sum_{j=1}^{N} p_{i,j} * \ln(p_{i,j}),\ \forall p_{i,j} > 0,$$

where N is the number of biological samples and $p_{i,j}$ is the fraction of the nearest neighbors of cell i that belong to sample j in the condition.

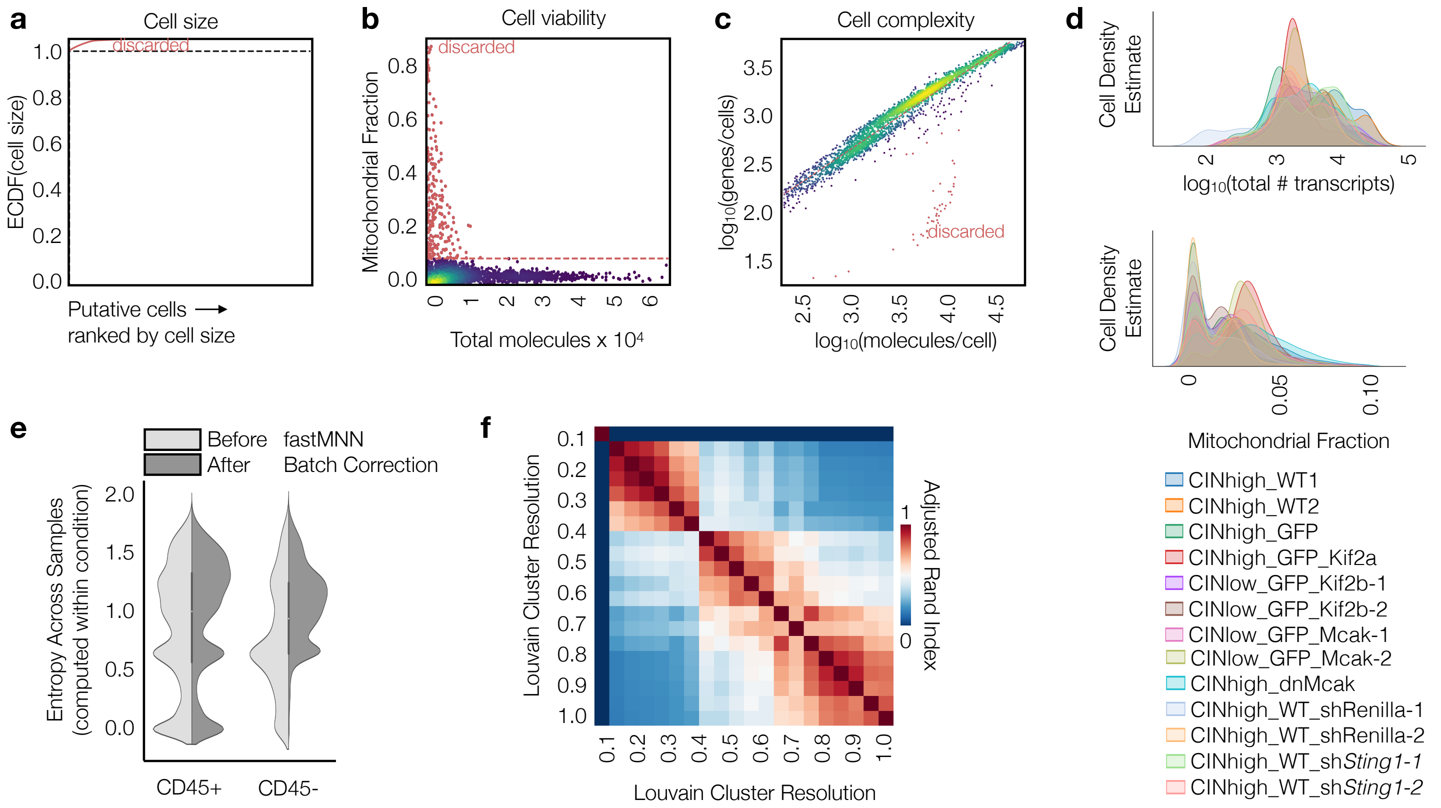

Supplementary Figure 4. Single cell pre-processing. a-c, Quality control plots for single cell RNA sequence data. Cells were filtered based on **(a)** cumulative number of transcript counts, **(b)** fraction of mitochondrial mRNA detected per cell and **(c)** cell complexity (*Pre-processing, cell selection and filtering of individual scRNA-seq samples*); shown here for one representative library. Excluded cells are labeled in red. Color on scatter plots represents cell density. **d,** Kernel Density Estimate (KDE) plots showing distribution across cells of total number of transcripts (top) and mitochondrial fraction (bottom) for each sample. **e,** Violin plots showing entropy of sample distribution within each condition, computed in a local neighborhood (nearest neighbor *k* = 30) around each cell in CD45+ (immune) and CD45- (tumor and stroma) cell subsets before and after applying fastMNN batch correction[5]. High entropy indicates most similar cells come from a well-mixed set of samples within the condition, whereas low entropy indicates most similar cells come from the same sample. **f,** Adjusted rand index (ARI) was used to assess robustness of Louvain cluster assignments[3,8] to cluster resolution *r* by evaluating co-occurrence of cells in clusters assigned across all pairwise values of *r.* For each pairwise comparison, we compute the ARI across all cells; visualized on a heatmap. Here we show optimization of Louvain cluster resolution for the macrophage subset (r = 0.15) because this was the first instance of *r* for which cell subtype categorical assignments were stable.

Supplementary Note 2 | Single cell RNA-seq macro cell type annotations.

CellAssign[9] was used to assign individual cells to all major cell types expected in murine TNBC models, without batch correction, based on a set of cell type-specific marker genes applied to the normalized count matrix. This cell type-marker gene list (annotated in **Supplementary Table 6**) was assembled using genes reported in the mouse cell atlas (MCA: http://bis.zju.edu.cn/MCA/), for all expected cell types in the TME, initially filtering for highly expressed and cell type specific genes using the Savitzky-Golay-smoothed first derivative filtering method described above (*Merged sample library generation)* for the pct_ratio (pct_1/pct_2) and avg_logFC. We then took the top 20 highest avg_logFC ranked genes and removed redundant genes widely expressed across multiple clusters and supplemented canonical markers curated from the literature, including keratins (K8, K18 and K19) to distinguish tumor-derived mammary epithelial cells[10]. CellAssign probabilistically assigns each cell, independent of clustering, to each of the 14 cell types in the gene marker file. Cells with probability assignments less than 95% were labeled "unassigned". We then evaluated the quality of these single cell assignments at the cluster level. Louvain clustering[1,3] computed on the top 50 principal components yielded 23 clusters and distinguished one small cluster (n = 1,174 cells) with a high fraction of unassigned cells (63%) and characteristically low average library size (< 1,000 molecules/cell); the unassigned cells (n = 745) from this cluster were removed as low-quality cells with an unclear phenotype. Otherwise, randomly dispersed unassigned cells were assigned to the mode cell assignment (excluding unassigned cells) of their Louvain cluster. More refined cell subtype annotations (**Fig. 2a**) were made on the full data matrix partitioned by cell type, to better characterize within-cell type biological variance (see **Supplementary Note 3**, *Within-cell type analyses*). The following phenotypically similar cell subsets were combined prior to within-cell type analyses: (1) macrophages and monocytes, (2) NK/T-cells with T-cells and NK-cells, and (3) Neutrophils and Gr-MDSCs. A filtering step was applied when evaluating clusters within each macro cell type to ensure high data quality. Specifically, three within-cell type Louvain clusters (comprising a total of 773 cells) exhibited features of apoptotic cells with low library size were removed and an additional 146 cells were clearly misassigned (clustering distinctly away from initial cell type annotation with other macro-cell types) and reassigned according to mode nearest neighbor annotations. Additionally, a contaminating subset of osteoclasts (n = 150 cells) were removed from downstream analyses. Final cell type assignments (**Fig. 2a**) were generated through one-to-one mapping of cell subtypes to the macro cell type for the global atlas. This yielded a filtered and annotated cell atlas comprised of 39,234 cells expressing 16,604 genes across 11 macro cell types and 28 cell subtypes (**Fig. 2a**) used for all downstream analyses.

Supplementary Note 3 | Single cell RNA-seq within-cell type analyses.

Phenotypes and state transitions were further characterized within each major cell type using Louvain clustering[1,3] (described here) or Markov state modeling of single cell trajectories using CellRank (see **Supplementary Note 4,** *Single cell trajectories using CellRank*)[11]. Within each major cell type, genes not expressed by any cells in the subset were removed and HVGs were computed per sample and merged across samples using the CellRanger flavor of scanpy[1], except for B cells that had HVGs computed across all samples together due to their small number (n < 10 cells) in some individual samples. Principal Component Analysis[12] was applied to the log-transformed count matrix, where the number of principal components were selected based on the knee point of the cumulative explained variance[13]. Diffusion components (DCs) were then computed on these top principal components, to better characterize major axes of variation within each cell type[14]. As implemented in Palantir[15], a cell-cell Euclidean distance matrix was computed based on the principal components of the log-transformed count matrix. An adaptive Gaussian kernel was then applied to convert distances into affinities, so that similarities between two cells decreases exponentially with distance. Finally, the affinity matrix was row-normalized

to construct a Markov transition matrix, whose eigenvectors are termed diffusion components. The optimal number of diffusion components required to explain variance within each cell type was determined using the eigengap method[15]. MAGIC imputation[16] was applied to selected diffusion components to further denoise and recover missing gene values using default parameters (t = 3, k = 15, implemented by Palantir), yielding a cell type-specific imputed count matrix for all genes. Louvain clustering was performed on principal components at twenty different cluster resolutions (r=0.05, 0.1, …, 1.0). The Adjusted Rand Index (ARI) was calculated for cluster assignments made across all pairs of r, creating a 20x20 matrix of ARI values that was used to select a value of r for which categorical assignments were stable. This was visualized as a two-dimensional heatmap (illustrated for macrophages in **Supplementary Fig. 4f**). Final cell subtype annotations were made at the level of Louvain clusters with the optimal cluster resolution r. The only exception to this approach was in the characterization of T cell subsets, which were alternatively annotated using the ProjecTILs[17] reference method (see below). The MAST[18] statistical framework was used to characterize differentially expressed genes (DEG) per cluster, compared to all other cells in the subset. MAST was applied to the log-transformed data and the Bonferroni correction was applied to correct for multiple hypothesis testing (p_{adj}). Gene set enrichment analysis (GSEA) was then performed for all DEG ranked and scored by:

$$-10log_{10}(p_{adj}) * log_2(fold\ change)$$

using cell type-specific pathway annotations (listed in **Supplementary Table 7**) to define associated biological processes. Specific details and exceptions to this generalized framework are outlined for all major cell types below.

<u>*Macrophages*</u>. Within the macrophage subset, 803 genes were not expressed in any cells and removed. This yielded a macrophage subset composed of 9,800 cells expressing 15,801 unique genes, of which 3,870 were highly variable across the cell type. The top 20 principal components and the top 2 diffusion components were used for downstream analyses. Louvain clustering was performed on selected principal components with a resolution of r=0.15, yielding three distinct macrophage subtypes, including two M1-like states and one M2-like state based on differentially expressed genes and pathways (**Extended Data Fig. 2d**).

<u>*Neutrophils/GR-MDSCs*</u>. Within the granulocytic subset, 2,428 genes were not expressed in any cells and removed. This yielded a PMN/GR-MDSC subset composed of 12,593 cells expressing 14,176 unique genes, of which 3,219 were highly variable. The top 16 principal components and 3 diffusion components were used for downstream analyses. Louvain clustering was performed on selected principal components at a resolution of r=0.35, yielding four distinct subtypes, including one ISG-Neutrophil and three granulocytic (Gr) MDSC subsets based on differentially expressed genes and pathways (**Fig. 2a**). When evaluating conditionally-dependent differential abundance of granulocytic subpopulations, the phenotypically similar MDSC subsets were merged (**Fig. 2b**).

<u>*Dendritic cells*</u>. Dendritic cells were composed of two transcriptionally distinct subpopulations of conventional dendritic cells (cDCs) and plasmacytoid dendritic cells (pDCs); which were treated separately following the generalized framework outlined above. Within the cDC subset, 2,829 genes were not expressed in any cells and removed, yielding a cDC subset of 762 cells expressing 13,775 unique genes, of which 3,938 were highly variable. Louvain clustering was performed on the top 13 principal components at a resolution of r=0.15, yielding three distinct cDC subclusters. These were merged with the pDC subset (313 cells) and HVGs were re-computed on the complete dendritic cell subset. Altogether, this yielded 1,075 dendritic cells expressing 14,227 unique genes, of which 3,652 were highly variable. The three cDC subtypes corresponded to the well characterized cDC1, cDC2 and activated (DC3, mregDC) dendritic cell states based on differential expression of canonical genes and pathways (**Extended Data Fig. 2h-i**).

T-cells. Rather than annotating T-cell phenotypic states based on Louvain clusters, which was challenging given their continuous nature and complex phenotypes, we projected the normalized count matrix of the T cell subset onto a comprehensive T cell reference atlas using the ProjecTILs[17] workflow (**Supplementary Fig. 5**). Pre-exhausted (CD8_Tpex) and terminally exhausted T cells (CD8_Tex) predicted by ProjecTILs were combined and annotated as "CD8+ Dysfunctional T cells", and CD4+ follicular helper (Tfh) and the CD4+ Th1-like cells (Th1) predicted by ProjecTIL were combined and annotated as "CD4+ Helper T cells." Trajectory inference was performed within the CD8+ T-cell subset (see **Supplementary Note 4,** *Single cell trajectories using CellRank*). This consisted of 4,797 CD8+ T-cells expressing 14,517 unique genes, of which 3,431 were highly variable. The top 14 principal components and 3 diffusion components computed within the CD8+ T-cell subset were used for downstream analyses.

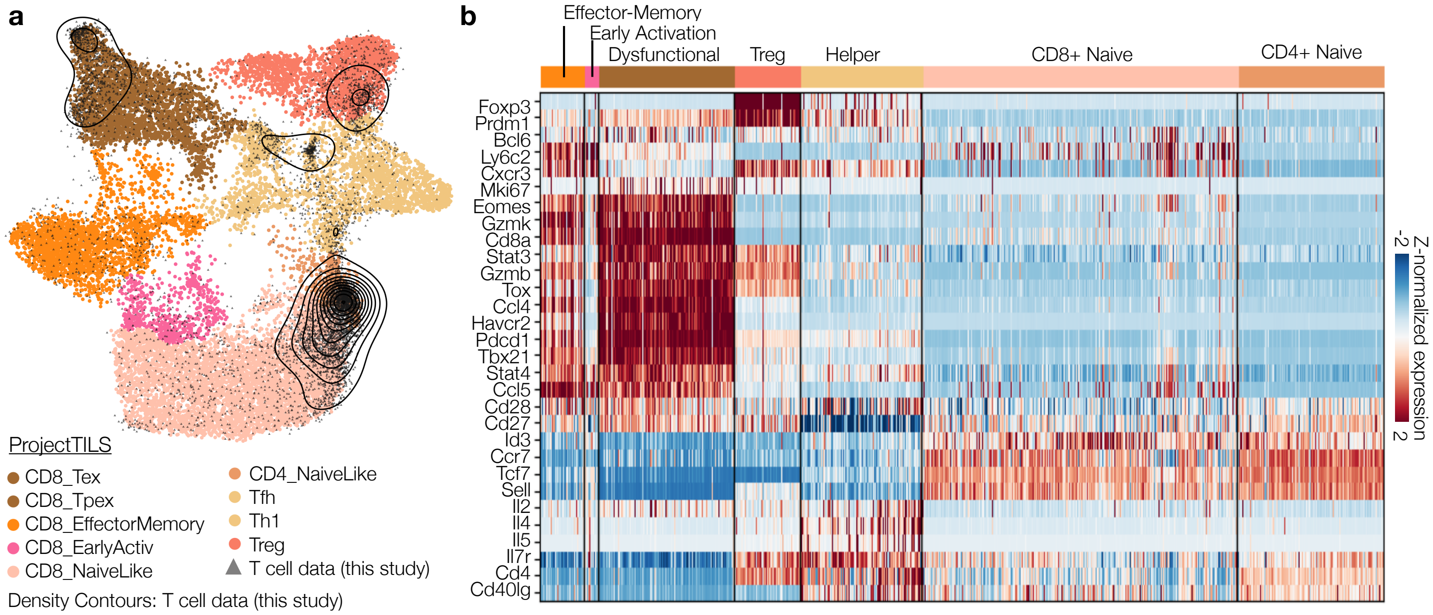

Supplementary Figure 5. Annotating T cells. a, UMAP projection of the complete ProjecTILs T cell reference atlas[17] colored by cell type with mapped T cell data from this study (n = 7,949 cells) overlaid as individual points and contours. **b,** Heatmap of canonical markers grouped by T cell subtype; genes were clustered by average Euclidean distance. Imputed expression was standardized by z-normalization for each gene across all T cells.

B-cells. Within the B cell subset, 3,868 genes were not expressed in any cells and removed. This yielded 2,337 B-cells expressing 12,736 unique genes, of which 3,251 were highly variable. Louvain clustering was performed on the top 11 principal components at a resolution of r = 0.25, which revealed one major IFN-responsive subpopulation and one bona fide plasma cell subpopulation, as well as three additional B cell states, based on differentially expressed genes and pathways (**Extended Data Fig. 3f**).

Tumor cells. Within the tumor subset, 801 genes were not expressed in any cells and removed. This yielded 3,596 tumor cells expressing 15,803 unique genes, of which 5,794 were highly variable across the subset. Louvain clustering was performed on the top 16 principal components within the tumor subset at a resolution of r=0.25, yielding five distinct tumor cell states – two mesenchymal stem-like states, one basal-like state, and two luminal-like subtypes, based on differentially expressed genes and pathways (**Extended Data Fig. 7b-e**). Diffusion components were likewise computed on the top 16 principal components. The top 3 diffusion components were selected for downstream analysis (see **Supplementary Note 4,** *Single cell trajectories using CellRank*).

Supplementary Note 4 | Single cell trajectories using CellRank.

For more in-depth analysis of cell state transitions and how these relate to tumor-host crosstalk, we performed trajectory analysis within four major cell types (macrophages, neutrophils/Gr-MDSCs, CD8+ T-cells, and tumor cells) using CellRank[11] and Palantir[15]. Initially, we explored using RNA splicing kinetics to infer directed cell-state trajectories using scVelo[19,20]. However, for all major cell types, the temporal response of pre-mRNAs being spliced into mature mRNA lacked the curvature necessary to confidently model RNA velocity per gene (dynamic, stochastic, or steady-state models). Instead RNA spliced vs. unspliced phase portraits were highly linear, a common scenario that limits inference of RNA velocity from single cell data[21]. Thus, we relied on Palantir[15] to determine the directionality of cell-state transitions, which requires prior biological knowledge of the initial cell state. Towards this, we first applied the Generalized Perron Cluster Cluster Analysis (G-PCCA)[22] to estimate cell macrostates based on the CellRank[11] connectivity kernel (undirected). Next, we selected the start cell for Palantir pseudotime with the max probability of belonging to the macrostate designated as initial based on prior phenotypic characterization of cell subtypes (i.e., **Extended Data Fig. 2d** and **Extended Data Fig. 7b-e**). The total number of macrostates was determined by the eigengap of the Schur decomposition of the CellRank connectivity kernel as previously described[11]. Palantir was then applied to diffusion components selected within each major cell type to compute the Palantir pseudotime cell-cell transition matrix using standard parameters (threshold scheme = 'hard', fraction to keep = 0.3). Finally, to reduce noise in the cell-cell transition matrix, the Palantir pseudotime and CellRank connectivity kernels were linearly combined (80:20 ratio) to generate a final combined transition matrix. This transition matrix is linearly embedded over low dimensional projections of the data as a vector stream using scVelo[19] for all velocity stream embeddings (**Extended Data Fig. 2d, Extended Data Fig. 3a, Extended Data Fig. 7e, and Supplementary Fig. 7a-b**). Macrostates were again computed using the G-PCCA estimator, but now based on the combined pseudotime and connectivity kernel (**Supplementary Fig. 6b**). Again, the number of coarse-grained macrostates was determined by the eigengap of the first 20 eigenvalues of its Schur decomposition. Terminal state probabilities were computed from this coarse-grained transition matrix using CellRank, whose built-in functions were also used for downstream analyses including identification of lineage-driver genes and plotting of gene-trend curves along cell state trajectories (**Extended Data Fig. 2e, Extended Data Fig. 2g, Extended Data Fig. 3b, Supplementary Fig. 7a-c**). To complete the CellRank workflow, the same methodology was implemented for the reverse combined kernel to estimate the initial state probability distribution (**Supplementary Fig. 7a**). Specific details and exceptions to this generalized framework for within-cell type trajectory analyses are outlined below.

Macrophages. Following the general outline described above, three macrostates were inferred from the macrophage connectivity kernel. The cell with the greatest initial state probability within the M1-like 1 macrostate was used to infer pseudotime from a pro-inflammatory state based on the top diffusion component using Palantir. Few macrophages showed an increased probability of expressing monocyte markers, according to CellAssign (described above). The M1-like 1 macrostate was used to seed the pseudotime trajectory because CellRank assigned them the max probability assignment to monocytes **(Extended Data Fig. 2d**). After combining the Palantir pseudotime kernel with the CellRank connectivity kernel (80:20 ratio) as described above, Schur decomposition of the combined kernel revealed one terminal state based on the large gap between the first and second eigenvalues. Thus, two macrostates (initial and terminal) were computed from the coarse-grained transition matrix. Of these, the M2-like macrostate was the most stable and labeled as a terminal state. Repeating the same process for the reverse combined kernel, two initial macrostates were identified belonging to M1-like subclusters (**Extended Data Fig. 2d**). Genes and pathways correlated with the Palantir pseudotime revealed interferon responsiveness as a major contributor to variation within this cell type (**Extended Data Fig. 2e**).

ISG-Neutrophils/Gr-MDSCs. Palantir pseudotime was not applied to the granulocytic subset because there was no biological basis to motivate selection of an initial cell state. Instead, G-PCCA was simply used to estimate the probability of each cell belonging to cell macrostates based on the undirected CellRank[11] connectivity kernel. Shur decomposition of the connectivity kernel revealed four stable macrostates (**Supplementary Fig. 6a**), consistent with four Louvain clusters previously characterized within this subset (**Supplementary Fig. 6b**). Gene set enrichment analysis along cells ranked by their probability of belonging to each granulocytic macrostate revealed mostly Gr-MDSCs, with one IFN-responsive subpopulation (ISG-Neutrophil) (**Extended Data Fig. 2g**).

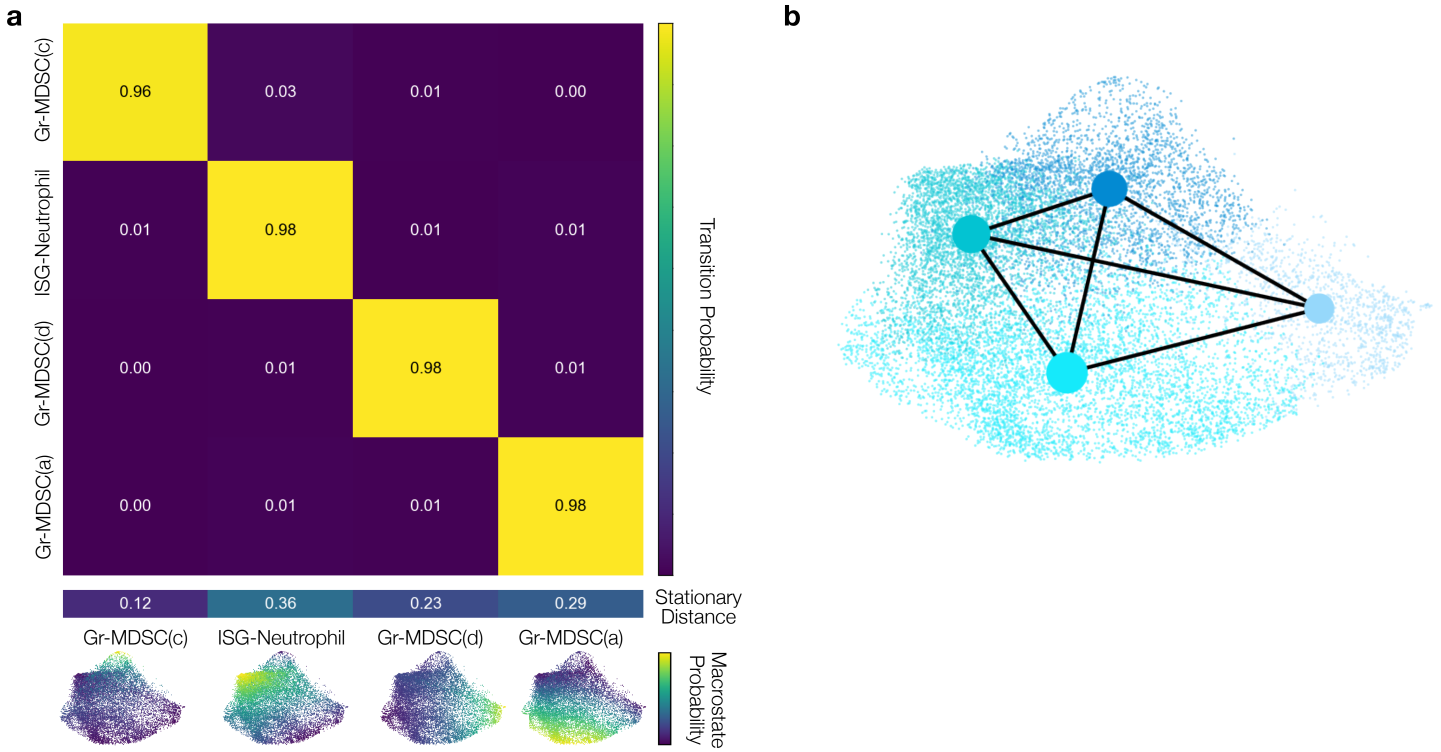

Supplementary Figure 6. Granulocytic infiltrate macro states. a, Coarse-grained macrostate transition matrix computed using CellRank[11] based on transcriptional similarity for all ISG-Neutrophils/Gr-MDSCs (n = 12,593 cells). Color bar below transition matrix shows the stationary distribution of the four identified macrostates as defined in [11]. Force-directed layouts[1,23] (lower) show the per-cell membership probability for each macrostate. **b,** Force-directed layout of all ISG-Neutrophils/Gr-MDSCs colored by subtype; Louvain clusters corresponds to the four stable macrostates. Overlaid partitioned-based graph abstraction (PAGA[24]) shows connectivity between subtypes with nodes scaled by relative subtype size and edge width scaled by connectivity confidence.

CD8+ T-cells. Within the annotated CD8+ T-cell subset, two macrostates were estimated by G-PCCA based on the CellRank[11] connectivity kernel. The cell with the highest initial state probability within the naïve T-cell subset was used to seed Palantir to infer the CD8+ T-cell differentiation trajectory (based on the top three diffusion components within this subset). Palantir was used to generate both a pseudotime kernel and differentiation kernel (1 – Palantir differentiation potential). Here, the differentiation kernel improved detection of the minority activated branch, which had less representation than the dominant dysfunctional branch. After linearly combining the Palantir pseudotime kernel with the Palantir differentiation kernel and CellRank connectivity kernel (40:40:20 ratio), Schur decomposition of the forward combined kernel revealed one initial (naïve) and two terminal CD8+ T-cell states (activated and dysfunctional). Repeating the same process for the reverse combined kernel, yielded a single initial macrostate corresponding to

the CD8+ Naïve population. Genes and pathways correlated with T-cells ranked by Palantir pseudotime reveal altered metabolism associated with dysfunction[25] (**Extended Data Fig. 3a-c** and **Supplementary Table 8**).

Tumor cells. Following the general outline described above, six macrostates were estimated in the tumor cells based on the CellRank[11] connectivity kernel. A trajectory from stem-like to more differentiated tumor lineages was inferred using Palantir based on the top three diffusion components. Palantir was initialized with a cell selected from Tumor Subtype C because it was distinguished by expression of canonical mammary stem cell markers and pathways (**Extended Data Fig. 7b-d**). After combining the Palantir pseudotime kernel with the CellRank connectivity kernel (80:20 ratio) as described above, Schur decomposition of the forward and reverse combined kernel revealed three terminal macrostates and one initial macrostate (**Supplementary Fig. 7**).

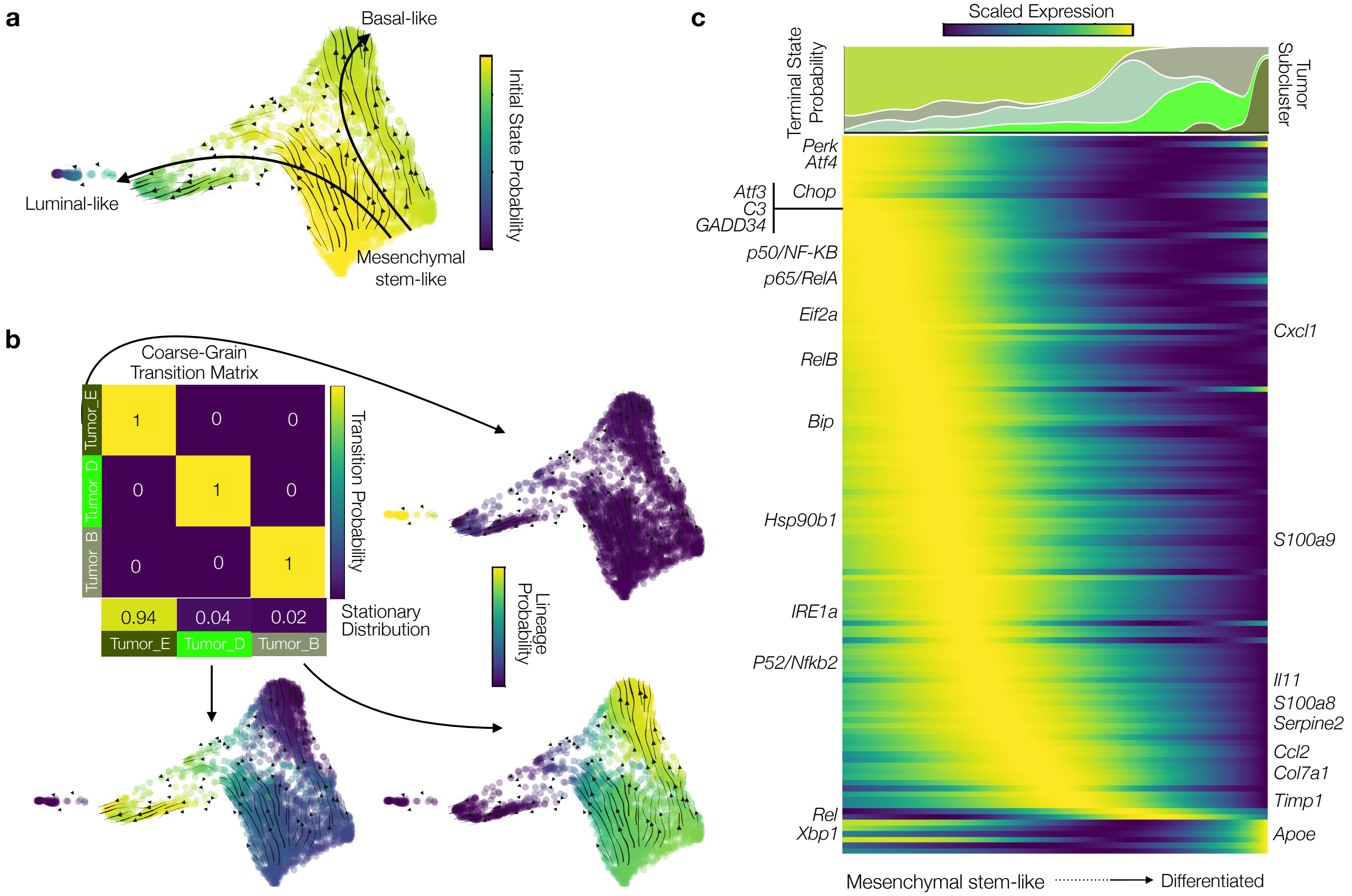

Supplementary Figure 7. Tumor cell differentiation from mesenchymal stem phenotype. a, Force-directed layout with CellRank transition vectors of all tumor cells colored by initial state probability. The overlayed arrows show two major branches from a more mesenchymal stem-like phenotype to more luminal- or basal-like states. Complete list of correlation ranked genes and gene set enrichment analysis (GSEA) results for cells ranked along this initial state probability, including nominal and corrected p-values, are provided in **Supplementary Table 8**. **b,** CellRank coarse-grained transition matrix for the forward combined connectivity and pseudotime kernel showing three stable terminal states and the long-term stationary distribution of these states over iterations of the Markov transition matrix, as previously described[11]. The probability distributions for each of these terminal states are projected onto three force-directed layouts with embedded transition vectors surround the heatmap. **c,** Scaled imputed expression of Unfolded

Protein Response genes (core genes, labeled *left*) and ligands emanating from CIN^{high} tumor cells that impart a significant CIN- and STING-dependent interaction effect on the TME as determined by ContactTracing (labeled *right*), for all tumor cells ranked by terminal state probability. Gene expression is smoothed using a GAM along Palantir pseudotime. Topmost stacked kernel density estimates show tumor subcluster assignment.

Supplementary Note 5 | Milo for differential abundance testing across conditions.

Conditionally-dependent differential abundance of major cell types was assessed in partially overlapping local neighborhoods on a *k*-nearest neighbor graph using the novel statistical framework Milo[26]; an approach insensitive to variations in cell numbers across samples (**Supplementary Fig. 8a-b**). We compared differential abundance of TME cell types when modulating tumor-intrinsic rates of CIN (CIN^{high} vs CIN^{low}) or STING in CIN^{high} tumor cells (*Sting1*^{WT} vs *Sting1*^{KD}) (two pairwise comparisons) using Milo with default parameters (k=10, p=0.1, and d=50). The CIN-and STING-dependent DA tests yielded 2,653 and 2,297 overlapping cell neighborhoods respectively, each characterized by a $\log_2$(fold change), p-value, and spatial FDR. DA neighborhoods were visualized on the UMAP computed on the full single-cell matrix or were mapped back to single cells for visualization by computing the median $\log_2$(fold change) and minimum p-value and spatial FDR across all neighborhoods to which the cell was mapped (**Supplementary Fig. 8a**). Milo[26] implements a refined sampling scheme to define a set of representative neighborhoods on the k-nearest neighbor graph. Thus, unsampled cells were assigned to the neighborhoods of their nearest neighbor. Conversely, neighborhoods were mapped to cell subtypes (**Supplementary Fig. 8c**) according to the mode subtype assignment of all cells within the neighborhood.

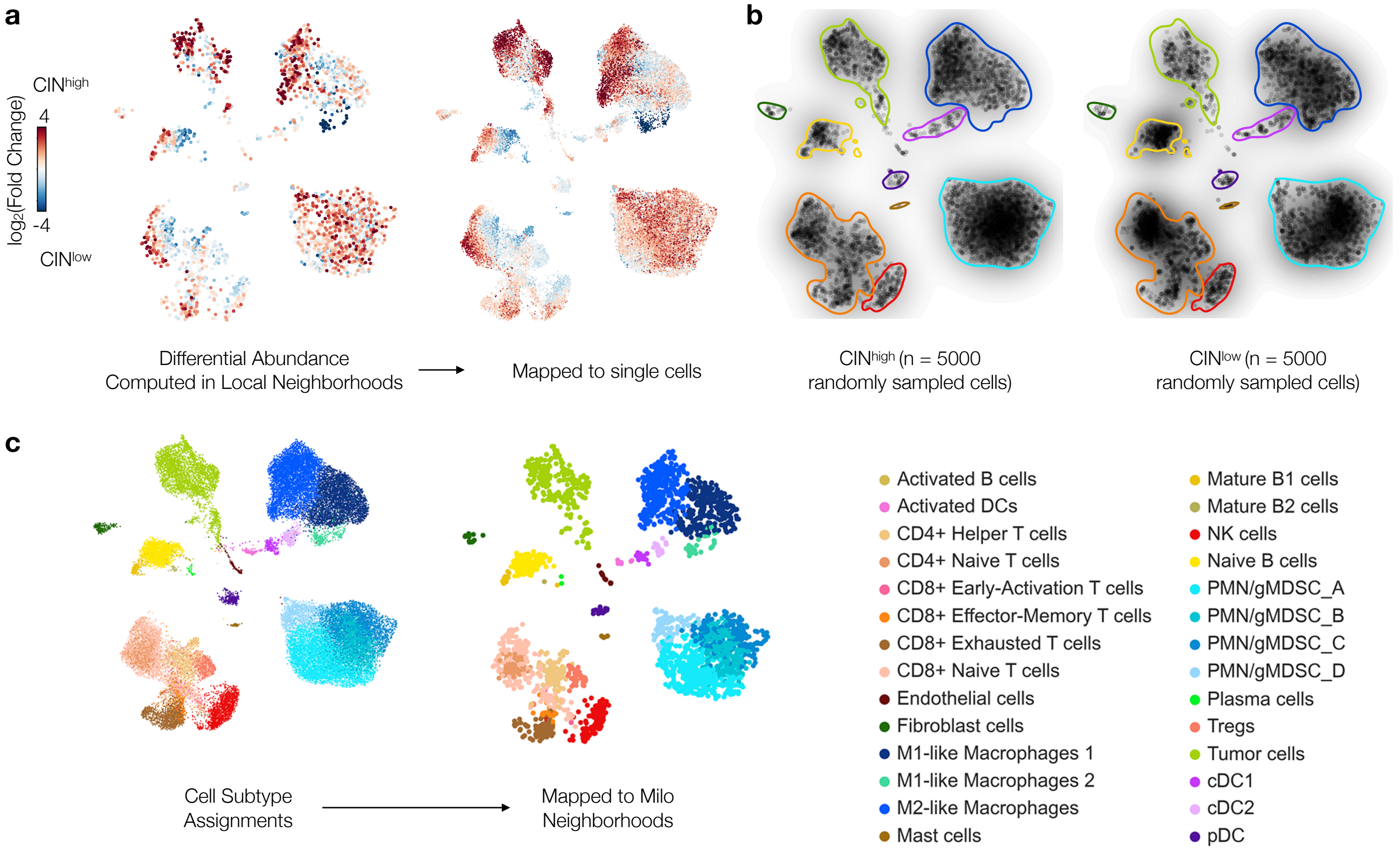

Supplementary Figure 8. Conditionally-dependent differential abundance testing. a, UMAP projections showing CIN-dependent differential abundance, $\log_2$(fold change) between CIN^{high} and CIN^{low} tumors, computed in local neighborhoods using Milo[26] (*left*) and

mapped to single cells (*right*). **b,** Cell density scatter plots showing random subsampling (n = 5,000 cells) from within the CIN^{high} and CIN^{low} conditions respectively to demonstrate Milo differential abundance measurements in **(a)** are consistent with within-condition relative cell densities independent of sample size. Overlaid contours color macro-level cell type annotations. **c,** UMAP projections showing cell subtype annotations determined at the single cell level (*left*) and mapped to Milo neighborhoods (*right*) based on the most common cell subtype in each neighborhood.

Supplementary Note 6 | Validation of anti-human-cGAS antibodies.

We sought to understand the impact of CIN-induced chronic STING signaling in human breast cancer. Prior reports have proposed that cGAS protein expression is lost in a large subset of tumors[27-29] based on immunohistochemical (IHC) analysis that is notable for its diffuse cytoplasmic pattern, in stark contrast to tumor cells in culture where cGAS is predominantly localized to micronuclei (**Extended Data Fig. 1f**). Using genetic depletion in paraformaldehyde-fixed MDA-MB-231 human TNBC cell pellets, we found diffuse cytoplasmic expression patterns in tumor cells to be non-specific and identified sensitive and specific anti-cGAS antibodies that appropriately recognize cGAS expression at micronuclei in human tumor samples (**Supplementary Fig. 9**). Specifically, cells of MDA-MD-231 dnMCAK-expressing control shRNA and MDA-MB-231 dnMCAK expressing *CGAS* shRNA were harvested from 15-cm dishes by cell scraper and washed with cold PBS. Then cells were resuspended in 4% Paraformaldehyde in PBS and were incubated at room temperature for 10 min. After replacing the fixation buffer with fresh 4% Paraformaldehyde, cells were incubated at 4°C overnight. Then the fixation buffer was replaced by 70% ethanol. After embedded by the low-melting agarose, cell pellets were processed to paraffin blocks and were sectioned by the Molecular Cytology Core Facility at MSKCC. Immunofluorescence expression with three different cGAS antibodies were performed individually as described before. Anti-cGAS antibody LS-757900 was applied with 1:200 dilution. Anti-cGAS antibodies ABF124 and HPA031700 were applied with concentration of 1ug/mL.

We investigated the protein expression of cGAS and STING in 179 human TNBCs for which we had long-term clinical follow-up data available. Prior reports had proposed that cGAS protein expression is lost in a large subset of tumors[27-29] based on immunohistochemical (IHC) analysis that is notable for its diffuse cytoplasmic pattern, in stark contrast to tumor cells in culture where cGAS is predominantly localized to micronuclei (e.g. **Extended Data Fig. 1f**). Using genetic depletion in paraformaldehyde-fixed MDA-MB-231 human TNBC cell pellets, we found diffuse cytoplasmic expression patterns of cGAS in tumor cells to be non-specific and identified sensitive and specific anti-cGAS antibodies that appropriately recognize cGAS expression in micronuclei in human tumors (**Extended Data Fig. 10d**). 92% of TNBC samples exhibited micronuclei with cGAS staining (cGAS+ micronuclei), arguing against its widespread loss in human cancer.

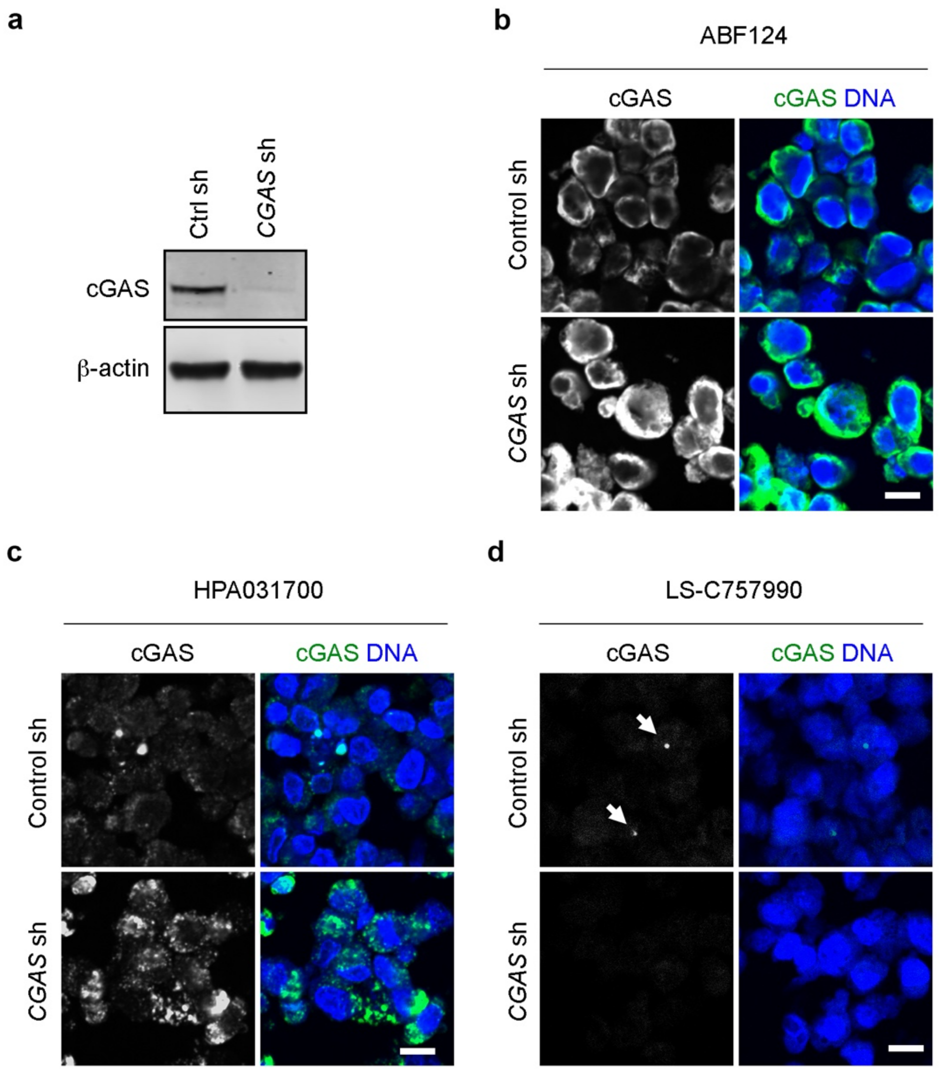

Supplementary Figure 9. Validation of anti-human-cGAS antibodies. a, Immunoblots for cGAS of control and *CGAS*-depleted MDA-MB-231 cell lysates with β-Actin as a loading control. **b-d,** Representative images of MDA-MB-231 TNBC cell pellets stained with DAPI (DNA) and three independent anti-human-cGAS antibodies; ABF124, HPA031700, and LS-C757990, respectively. White arrows denote cGAS expression in micronuclei. Scale bars, 10 μM.

Supplementary Table 1: shRNA hairpins and crRNA guide sequences

Gene target	crRNA vs. shRNA	Sequence
Cgas	crRNA	GCGAGGGTCCAGGAAGGAAC
Cgas	shRNA	TTCATATTCAATTTTCTTCGAT
Cgas	shRNA	TTCATAATATTCTTGTAGCTCA
Sting1	crRNA	CTACATAACAACATGCTCAG
Sting1	shRNA	TATATAAAATCCTTTGGCTGGG
Sting1	shRNA	TTCTACATATATAAAATCCTTT
Atf6	crRNA	AACACGAGTCTGTGGACCG
Atf6	crRNA	AGCAGACGACAGAACCGCA
Atf6	crRNA	AGAGTATATGCTAGGACTGG
Ern1 (Ire1α)	crRNA	CATGTATGACACCAAGACCC
Ern1 (Ire1α)	crRNA	TACTTCCCAACATACAGAGT
Ern1 (Ire1α)	crRNA	AGAGGATTCCATCTGAACTT
Eif2ak3 (Perk)	crRNA	AATATACCGAAGTTCAAAG
Eif2ak3 (Perk)	crRNA	CATGACCTTCCAATCAGCAA
Eif2ak3 (Perk)	crRNA	ATGGCGTTTAGTAGGAAGGG
Ccl2	crRNA	CAACACGTGGATGCTCCAGC
Ccl2	crRNA	GATGATCCCAATGAGTAGGC
Cxcl1	crRNA	AGGGCAACACCTTCAAGCTC
Cxcl1	crRNA	CTTGAGGTGAATCCCAGCCA
Il11	crRNA	CCAGGGGCAACGACTCTATC
Il11	crRNA	CCTACCTCCGGCATGTACAA

Supplementary Table 2: Antibodies used in immunoblots

**Antibodies against**	**Clone**	**Company**	**Catalog number**	**Dilution**
Mouse cGAS	D3O8O	Cell Signaling Technology	31659	1:1000
β-actin	AC-15	Abcam	ab6276	1:5000
STING	D2P2F	Cell Signaling Technology	13647	1:1000
STING	D1V5L	Cell Signaling Technology	50494	1:1000
α-tubulin	DM1A	Sigma-Aldrich	T9026	1:2000
p-PERK (Thr980)	16F8	Cell Signaling Technology	3179	1:1000
PERK	C33E10	Cell Signaling Technology	3192	1:1000
BiP	C50B12	Cell Signaling Technology	3177	1:1000
CHOP	L63F7	Cell Signaling Technology	2895	1:1000
ATF4	D4B8	Cell Signaling Technology	11815	1:1000
p-eIF2α (Ser51)	119A11	Cell Signaling Technology	3597	1:1000
eIF2α	D7D3	Cell Signaling Technology	5324	1:1000
CoxIV	polyclonal	Abcam	ab16056	1:1000

Supplementary Table 3: Antibodies used in immunofluorescence

Antibodies against	Clone	Company	Catalog number	Lot number	Dilution
Mouse cGAS	D3O8O	Cell Signaling Technology	31659		1:1000
Human cGAS	Polyclonal	Millipore Sigma	ABF124	3168722	1:200
Human cGAS	Polyclonal	Sigma Aldrich	HPA031700	D117238	1:200
Human cGAS	1697CT136.65.30	LSBio	LS-C757990	164559	1:200
Human centromere proteins		Antibodies Incorporated	15-234-0001		1:1000
STING	D2P2F	Cell Signaling Technology	13647		1:1000

Supplementary Table 4: Primer sequences used in qPCR

Target	Primer 1	Primer2
mouse Ccl5	5’-GCTGCTTTGCCTACCTCTCC-3’	5’-TCGAGTGACAAACACGACTGC-3’
mouse Cxcl10	5’-CCAAGTGCTGCCGTCATTTTC-3’	5’-GGCTCGCAGGGATGATTTCAA-3’
mouse Isg15	5-AAAGAAGCAGATTGCCCAGAA-3’	5’-TCTGCGTCAGAAAGACCTCA-3’
mouse Gapdh	5’-AGGTCGGTGTGAACGGATTTG-3’	5’-TGTAGACCATGTAGTTGAGGTCA-3’
human sXbp1	5’-CTGAGTCCGAATCAGGTGCAG-3’	5’-ATCCATGGGGAGATGTTCTGG-3’
human Atf3	5’-CCTCTGCGCTGGAATCAGTC-3’	5’TTCTTTCTCGTCGCCTCTTTTT-3’
human CHOP	5’-AGAACCAGGAAACGGAAACAGA-3’	5’-TCTCCTTCATGCGCTGCTTT-3’
human BiP	5’-TGTTCAACCAATTATCAGCAAACTC-3’	5’-TTCTGCTGTATCCTCTTCACCAGT-3’
human GUSB	5'-CTGACACCTCCAAGTATCCCAAG	5'-GTCGTGTACAGAAGTACAGACCGC-3'
human TNF α	5'-CCAGGGACCTCTCTCTAATCAGC	5'-GGTTTGCTACAACATGGGCTAC-3'
human INF1B	5'-TACTGCCTCAAGGACAGGATGAA	5'-GCATCTCATAGATGGTCAATGCG-3'
human Ccl2	5'-CTGTGATCTTCAAGACCATTGTG	5'-AGTTTGGGTTTGCTTGTCCAG-3'
human Oas2	5'-GAGCCAGTTGCAGAAAACCAG	5'-GCATTGTCGGCACTTTCCAA-3'
human Oas3	5'-GAAGCCCAGGCCTATCATCC	5'-TCATCCAGTAGGACCGCTGA-3'
human Cxcl10	5'-AGGAGTACCTCTCTCTAGAAC	5'-AAAGACCTTGGATTAACAGG-3'
human Ccl5	5'-GTCTTTGTCACCCGAAAG	5'-GACAAGAGCAAGCAGAAAC-3'
human Isg15	5'-CTCTGAGCATCCTGGTGAGGAA	5'-AAGGTCAGCCAGAACAGGTCGT-3'

Supplementary Table 5: Antibodies used in flow cytometry analysis

Antibodies against	Conjugated fluorophore	Clone	Company	Catalog number	Dilution
CD11b	PE-Cyanine7	M1/70	Thermo Fisher Scientific	25-0112-82	1:200
Ly-6G	APC	1A8-Ly6g	Thermo Fisher Scientific	17-9668-82	1:200
CD80	Brilliant Violet 650	16-10A1	BioLegend	104732	1:200
CD206	PE	C068C2	Biolegend	141706	1:200
F4/80	PE/Cyanine5	BM8	Biolegend	123111	1:200
MHC class II	Brilliant Violet 605	M5/114.15.2	BioLegend	107639	1:200
CD44	Alexa Fluor® 647	IM7	BioLegend	103017	1:200
NKp46	Brilliant Violet 510	29A1.4	BioLegend	137623	1:100

CD8a	Brilliant Violet 421	QA17A07	BioLegend	155010	1:200
CD45RB	PerCP/Cyanine5.5	C363-16A	BioLegend	103313	1:200
CD45R/B220	Alexa Fluor® 700	RA3-6B2	BioLegend	103231	1:200
Gr-1	APC/Fire™ 750	RB6-8C5	BioLegend	108455	1:200
**CD16/32**		93	BioLegend	101319	1:200

REFERENCES

1 Wolf, F. A., Angerer, P. & Theis, F. J. SCANPY: large-scale single-cell gene expression data analysis. *Genome Biol* **19**, 15, doi:10.1186/s13059-017-1382-0 (2018).

2 Wolock, S. L., Lopez, R. & Klein, A. M. Scrublet: Computational Identification of Cell Doublets in Single-Cell Transcriptomic Data. *Cell Syst* **8**, 281-291 e289, doi:10.1016/j.cels.2018.11.005 (2019).

3 Levine, J. H. *et al.* Data-Driven Phenotypic Dissection of AML Reveals Progenitor-like Cells that Correlate with Prognosis. *Cell* **162**, 184-197, doi:10.1016/j.cell.2015.05.047 (2015).

4 Virtanen, P. *et al.* SciPy 1.0: fundamental algorithms for scientific computing in Python. *Nat Methods* **17**, 261-272, doi:10.1038/s41592-019-0686-2 (2020).

5 Haghverdi, L., Lun, A. T. L., Morgan, M. D. & Marioni, J. C. Batch effects in single-cell RNA-sequencing data are corrected by matching mutual nearest neighbors. *Nat Biotechnol* **36**, 421-427, doi:10.1038/nbt.4091 (2018).

6 Laughney, A. M. *et al.* Regenerative lineages and immune-mediated pruning in lung cancer metastasis. *Nat Med* **26**, 259-269, doi:10.1038/s41591-019-0750-6 (2020).

7 Tirosh, I. *et al.* Dissecting the multicellular ecosystem of metastatic melanoma by single-cell RNA-seq. *Science* **352**, 189-196, doi:10.1126/science.aad0501 (2016).

8 Traag, V. A., Waltman, L. & van Eck, N. J. From Louvain to Leiden: guaranteeing well-connected communities. *Sci Rep* **9**, 5233, doi:10.1038/s41598-019-41695-z (2019).

9 Zhang, A. W. *et al.* Probabilistic cell-type assignment of single-cell RNA-seq for tumor microenvironment profiling. *Nat Methods* **16**, 1007-1015, doi:10.1038/s41592-019-0529-1 (2019).

10 Trask, D. K. *et al.* Keratins as markers that distinguish normal and tumor-derived mammary epithelial cells. *Proc Natl Acad Sci U S A* **87**, 2319-2323, doi:10.1073/pnas.87.6.2319 (1990).

11 Lange, M. *et al.* CellRank for directed single-cell fate mapping. *bioRxiv*, 2020.2010.2019.345983, doi:10.1101/2020.10.19.345983 (2020).

12 Halko N, M. P., Tropp JA. Finding structure with randomness: Probabilistic algorithms for constructing apprxoimate matrix decompositions. *ARXIV* (2009).

13 Valle, S., Li, W. H. & Qin, S. J. Selection of the number of principal components: The variance of the reconstruction error criterion with a comparison to other methods. *Ind Eng Chem Res* **38**, 4389-4401, doi:DOI 10.1021/ie990110i (1999).

14 Coifman, R. R. *et al.* Geometric diffusions as a tool for harmonic analysis and structure definition of data: diffusion maps. *Proc Natl Acad Sci U S A* **102**, 7426-7431, doi:10.1073/pnas.0500334102 (2005).

15 Setty, M. *et al.* Characterization of cell fate probabilities in single-cell data with Palantir. *Nat Biotechnol* **37**, 451-460, doi:10.1038/s41587-019-0068-4 (2019).

16 van Dijk, D. *et al.* Recovering Gene Interactions from Single-Cell Data Using Data Diffusion. *Cell*, doi:10.1016/j.cell.2018.05.061 (2018).

17 Andreatta, M. *et al.* Interpretation of T cell states from single-cell transcriptomics data using reference atlases. *Nat Commun* **12**, 2965, doi:10.1038/s41467-021-23324-4 (2021).

18 Finak, G. *et al.* MAST: a flexible statistical framework for assessing transcriptional changes and characterizing heterogeneity in single-cell RNA sequencing data. *Genome Biol* **16**, 278, doi:10.1186/s13059-015-0844-5 (2015).

19 Bergen, V., Lange, M., Peidli, S., Wolf, F. A. & Theis, F. J. Generalizing RNA velocity to transient cell states through dynamical modeling. *Nat Biotechnol* **38**, 1408-1414, doi:10.1038/s41587-020-0591-3 (2020).

20 La Manno, G. *et al.* RNA velocity of single cells. *Nature* **560**, 494-498, doi:10.1038/s41586-018-0414-6 (2018).

21 Bergen, V., Soldatov, R. A., Kharchenko, P. V. & Theis, F. J. RNA velocity-current challenges and future perspectives. *Mol Syst Biol* **17**, e10282, doi:10.15252/msb.202110282 (2021).

22 Reuter, B., Weber, M., Fackeldey, K., Roblitz, S. & Garcia, M. E. Generalized Markov State Modeling Method for Nonequilibrium Biomolecular Dynamics: Exemplified on Amyloid beta Conformational Dynamics Driven by an Oscillating Electric Field. *J Chem Theory Comput* **14**, 3579-3594, doi:10.1021/acs.jctc.8b00079 (2018).

23 Jacomy, M., Venturini, T., Heymann, S. & Bastian, M. ForceAtlas2, a continuous graph layout algorithm for handy network visualization designed for the Gephi software. *PLoS One* **9**, e98679, doi:10.1371/journal.pone.0098679 (2014).
24 Wolf, F. A. *et al.* PAGA: graph abstraction reconciles clustering with trajectory inference through a topology preserving map of single cells. *Genome Biol* **20**, 59, doi:10.1186/s13059-019-1663-x (2019).
25 Azizi, E. *et al.* Single-Cell Map of Diverse Immune Phenotypes in the Breast Tumor Microenvironment. *Cell* **174**, 1293-1308 e1236, doi:10.1016/j.cell.2018.05.060 (2018).
26 Dann, E., Henderson, N. C., Teichmann, S. A., Morgan, M. D. & Marioni, J. C. Differential abundance testing on single-cell data using k-nearest neighbor graphs. *Nat Biotechnol*, doi:10.1038/s41587-021-01033-z (2021).
27 Schadt, L. *et al.* Cancer-Cell-Intrinsic cGAS Expression Mediates Tumor Immunogenicity. *Cell Rep* **29**, 1236-1248 e1237, doi:10.1016/j.celrep.2019.09.065 (2019).
28 Xia, T., Konno, H. & Barber, G. N. Recurrent Loss of STING Signaling in Melanoma Correlates with Susceptibility to Viral Oncolysis. *Cancer Res* **76**, 6747-6759, doi:10.1158/0008-5472.CAN-16-1404 (2016).
29 Xia, T., Konno, H., Ahn, J. & Barber, G. N. Deregulation of STING Signaling in Colorectal Carcinoma Constrains DNA Damage Responses and Correlates With Tumorigenesis. *Cell Rep* **14**, 282-297, doi:10.1016/j.celrep.2015.12.029 (2016).
