# Supplementary material for: Non-cell-autonomous cancer progression from chromosomal instability
Source: Nature. 2023 Aug 23;620(7976):1080–8. doi: 10.1038/s41586-023-06464-z (PMC10468402; doi:10.1038/s41586-023-06464-z)
Supplement: Supplementary file 2 — Reporting Summary [file 41586_2023_6464_MOESM2_ESM.pdf]

nature portfolio

Corresponding author(s): Samuel Bakhoum and Ashley Laughney

Last updated by author(s): Jun 21, 2023

Reporting Summary

Nature Portfolio wishes to improve the reproducibility of the work that we publish. This form provides structure for consistency and transparency in reporting. For further information on Nature Portfolio policies, see our Editorial Policies and the Editorial Policy Checklist.

Statistics

For all statistical analyses, confirm that the following items are present in the figure legend, table legend, main text, or Methods section.

n/a	Confirmed	
☐	☒	The exact sample size (*n*) for each experimental group/condition, given as a discrete number and unit of measurement
☐	☒	A statement on whether measurements were taken from distinct samples or whether the same sample was measured repeatedly
☐	☒	The statistical test(s) used AND whether they are one- or two-sided<br>*Only common tests should be described solely by name; describe more complex techniques in the Methods section.*
☐	☒	A description of all covariates tested
☐	☒	A description of any assumptions or corrections, such as tests of normality and adjustment for multiple comparisons
☐	☒	A full description of the statistical parameters including central tendency (e.g. means) or other basic estimates (e.g. regression coefficient) AND variation (e.g. standard deviation) or associated estimates of uncertainty (e.g. confidence intervals)
☐	☒	For null hypothesis testing, the test statistic (e.g. *F*, *t*, *r*) with confidence intervals, effect sizes, degrees of freedom and *P* value noted<br>*Give P values as exact values whenever suitable.*
☐	☒	For Bayesian analysis, information on the choice of priors and Markov chain Monte Carlo settings
☐	☒	For hierarchical and complex designs, identification of the appropriate level for tests and full reporting of outcomes
☐	☒	Estimates of effect sizes (e.g. Cohen's *d*, Pearson's *r*), indicating how they were calculated

Our web collection on statistics for biologists contains articles on many of the points above.

Software and code

Policy information about availability of computer code

Data collection	Western blots were captured using ImageStudio software version 5.2 and Amersham ImageQuant™ 800 version 1.2.<br>Bioluminescence images were collected with IVIS Lumina LT Inst, Series III.<br>Zen 3.4 Blue Edition for image capture.<br>SpectroFlo (Cytek Biosciences) was used for flow cytometry data acquisition.<br><br>CellRanger (v3.1.0) was utilized to construct a count matrix from raw reads, including sample demultiplexing, alignment to CellRanger's mm10-3.0.0 reference (GRCm38, available as a CellRanger reference package on 10X Genomics Downloads page), barcode processing, and the generation of a raw digital expression matrix by collapsing groups of reads with the same unique molecular identifier (UMI), cell barcode and gene annotation. The count matrix was then loaded into python using scanpy (v1.7.2) (read_10x_h5) for subsequent pre-processing and downstream analysis.
Data analysis	Western blots images were analyzed with ImageJ (1.52m 20) and ImageStudio software version 5.2<br><br>Bulk RNA sequencing analysis of B16F10 was done with:<br>Broad Picard Pipeline (2.19.1)<br>GenomicAlignments (v1.18.1)<br>DESeq2 (v1.24.0)<br><br>Flow cytometry data was analyzed with FlowJo software, version 10.8.2.

Custom code, including docker environments with jupyter notebooks demonstrating the ContactTracing method, are available on the Laughney Lab GitHub (https://github.com/LaughneyLab/ContactTracing_tutorial, https://zenodo.org/record/8061480). Code for the interactive web dashboard is available on GitHub (https://github.com/LaughneyLab/ContactTracing-Viz/, https://zenodo.org/record/8067675), respectively, Circos software93 (v0.69-9) was used to visualize ligand-receptor interactions based on measured transcriptional responses (ContactTracing). Network plot in Extended Data Fig. 4h was created with Cytoscape94 v3.8.2.

For manuscripts utilizing custom algorithms or software that are central to the research but not yet described in published literature, software must be made available to editors and reviewers. We strongly encourage code deposition in a community repository (e.g. GitHub). See the Nature Portfolio guidelines for submitting code & software for further information.

Data

Policy information about availability of data

All manuscripts must include a data availability statement. This statement should provide the following information, where applicable:

- Accession codes, unique identifiers, or web links for publicly available datasets
- A description of any restrictions on data availability
- For clinical datasets or third party data, please ensure that the statement adheres to our policy

All single cell RNA sequencing data generated in this study have been deposited in the NCBI's Gene Expres-sion Omnibus (GEO) database under accession code: GSE189856 (https://www.ncbi.nlm.nih.gov/geo/query/acc.cgi?acc=GSE189856).

The GRCm38 genome reference is available as a CellRanger reference package (version mm10-3.0.0).

All single cell RNA sequencing from the independent human cohort is available in the NCBI's GEO under accession code: GSE176078, and the spatial data from the same study is at https://zenodo.org/record/4739739.

CellPhoneDb can be found at https://www.cellphonedb.org (v2.1.4 was used for this study), and celltalkdb database is at http://tcm.zju.edu.cn/celltalkdb/download.php.

An interactive web dashboard is made available at http://contacttracing.laughneylab.com/ to enable interactive exploration of data from this study, allowing users to visualize pair-wise ligand-receptor-mediated interactions and systems-level interactions in circos plots (like Fig. 4a and Extended Data Fig. 12e) using plotly v5.11.0 and dash v2.7.1. Processed scRNA-seq data sets appropriate for input to the ContactTracing method are available at https://doi.org/10.5281/zenodo.8061222.

Research involving human participants, their data, or biological material

Policy information about studies with human participants or human data. See also policy information about sex, gender (identity/presentation), and sexual orientation and race, ethnicity and racism.

Reporting on sex and gender	N/A
Reporting on race, ethnicity, or other socially relevant groupings	N/A
Population characteristics	This was an existing tissue microarray of breast tumor samples
Recruitment	N/A
Ethics oversight	Memorial Sloan Kettering Cancer Center Institutional Review Board

Note that full information on the approval of the study protocol must also be provided in the manuscript.

Field-specific reporting

Please select the one below that is the best fit for your research. If you are not sure, read the appropriate sections before making your selection.

☒ Life sciences ☐ Behavioural & social sciences ☐ Ecological, evolutionary & environmental sciences

For a reference copy of the document with all sections, see nature.com/documents/nr-reporting-summary-flat.pdf

Life sciences study design

All studies must disclose on these points even when the disclosure is negative.

Sample size	Power analysis was used to estimate the numbers of animals for survival experiments. For metastasis experiments relying on the tumor burden or lung surface metastasis number, the animal numbers were estimated based on prior experience with the models
Data exclusions	Cell selection and filtering are detailed in the Methods section. Viable cells were distinguished from droplets consisting of ambient mRNA transcripts arising in solution due to premature lysis or cell death based on library size. Cells were ranked by library size (total molecule

counts) in descending order. We then computed the first and second derivative of the normalized sum of this array (based on average of a 10-cell rolling window) and identified the inflection point, or first instance in which the second derivative is zero. All cells with a library size less than 0.9X the inflection point were discarded. Additionally, cells with > 10% of transcriptions derived from mitochondria cells with low coverage, or cells with low complexity libraries (in which detected molecules align to a small subset of genes determined by at least 0.4X standard deviations from a linear fit) were discarded. Following single sample pre-processing and filtering, all biological samples (n = 13 murine tumors) were merged. Cell doublet scores computed in individual samples were subsequently assessed at the cluster and single cell level for the merged library. Three clusters distinguished by high average double score and individual cells with doublet scores greater a threshold were removed. Altogether, this resulted in the removal of 474 putative doublet cells from the merged cell atlas. Louvain clustering distinguished one small cluster (n = 1,174 cells) with a high fraction of unassigned cells (63%) and characteristically low average library size (< 1,000 molecules/cell); (n=745) unassigned cells from this cluster were removed as low-quality cells with an unclear phenotype. Three within-cell type clusters (comprising a total of 773 cells) exhibited features of apoptotic cells with low library size and were removed. Additionally, a contaminating subset of osteoclasts (n = 150 cells) were removed from downstream analyses.

This strategy resulted in 39,234 cells obtained from 14 surgically removed mouse primary tumor samples.

Replication

No attempts for the replication failed. Replicates were stated in the figure legends or in the Method section.

Randomization

For in vivo experiments, animals were randomly assigned to different groups.

Blinding

Investigators were not blind to group allocation as this information was essential for experiment conducting.

Reporting for specific materials, systems and methods

We require information from authors about some types of materials, experimental systems and methods used in many studies. Here, indicate whether each material, system or method listed is relevant to your study. If you are not sure if a list item applies to your research, read the appropriate section before selecting a response.

Materials & experimental systems

n/a	Involved in the study	
☐	☒	Antibodies
☐	☒	Eukaryotic cell lines
☒	☐	Palaeontology and archaeology
☐	☒	Animals and other organisms
☐	☒	Clinical data
☒	☐	Dual use research of concern
☒	☐	Plants

Methods

n/a	Involved in the study	
☒	☐	ChIP-seq
☐	☒	Flow cytometry
☒	☐	MRI-based neuroimaging

Antibodies

Antibodies used

Following antibodies were used in immunoblots:
Anti-Mouse cGAS (Cell Signaling Technology, Cat# 31659, D3O8O)
Anti-β-actin (Abcam, Cat# ab6276, AC-15)
Anti-STING (Cell Signaling Technology, Cat# 13647, D2P2F)
Anti-STING (Cell Signaling Technology, Cat# 50494, D1V5L)
Anti-α-tubulin (Sigma-Aldrich, Cat# T9026, DM1A)
Anti-p-PERK (Thr980) (Cell Signaling Technology, Cat# 3179, 16F8)
Anti-PERK (Cell Signaling Technology, Cat# 3192, C33E10)
Anti-BiP (Cell Signaling Technology, Cat# 3177, C50B12)
Anti-CHOP (Cell Signaling Technology, Cat# 2895, L63F7)
Anti-ATF4 (Cell Signaling Technology, Cat# 11815, D4B8)
Anti-p-eIF2α (Ser51) (Cell Signaling Technology, Cat# 3597, 119A11)
Anti-eIF2α (Cell Signaling Technology, Cat# 5324, D7D3)
Anti-CoxIV (Abcam, Cat# ab16056, polyclonal)

Following antibodies were used in immunofluorescence staining:
Anti-Mouse cGAS (Cell Signaling Technology, Cat# 31659,D3O8O)
Anti-Human cGAS (Millipore Sigma, Cat# ABF124,Polyclonal) Lot# 3168722
Anti-Human cGAS (Sigma Aldrich, Cat# HPA031700,Polyclonal) Lot# D117238
Anti-Human cGAS (LSBio, Cat# LS-C757990,1697CT136.65.30) Lot# 164559
Anti-Human centromere proteins (Antibodies Incorporated, Cat# 15-234-0001,)
Anti-STING (Cell Signaling Technology, Cat# 13647,D2P2F)

Following antibodies were used in the flow cytometry analysis:
Anti-CD11b-PE-Cyanine7 (Thermo Fisher Scientific, Cat# 25-0112-82, M1/70)
Anti-Ly-6G-APC (Thermo Fisher Scientific, Cat# 17-9668-82, 1A8-Ly6g)
Anti-CD80-Brilliant Violet 650 (BioLegend, Cat# 104732, 16-10A1)
Anti-CD206-PE (Biolegend, Cat# 141706, C068C2)
Anti-F4/80-PE/Cyanine5 (Biolegend, Cat# 123111, BM8)

Anti-MHC class II-Brilliant Violet 605 (BioLegend, Cat# 107639, M5/114.15.2)
Anti-CD44-Alexa Fluor® 647 (BioLegend, Cat# 103017, IM7)
Anti-NKp46-Brilliant Violet 510 (BioLegend, Cat# 137623, 29A1.4)
Anti-CD8a-Brilliant Violet 421 (BioLegend, Cat# 155010, QA17A07)
Anti-CD45RB-PerCP/Cyanine5.5 (BioLegend, Cat# 103313, C363-16A)
Anti-CD45R/B220-Alexa Fluor® 700 (BioLegend, Cat# 103231, RA3-6B2)
Anti-Gr-1-APC/Fire™ 750 (BioLegend, Cat# 108455, RB6-8C5)
Anti-CD16/32 (BioLegend, Cat# 101319, 93)

Validation

The primary antibodies used were purchased from reputable sources validated for the species and application (immunoblotting, flow cytometry, or immunofluorescence) by their respective manufacturers in their website's validation statements.

Anti-mouse CD11b-PE-Cyanine7 (https://www.thermofisher.com/antibody/product/CD11b-Antibody-clone-M1-70-Monoclonal/25-0112-82)
Anti-mouse Ly-6G-APC (https://www.thermofisher.com/antibody/product/Ly-6G-Antibody-clone-1A8-Ly6g-Monoclonal/17-9668-82)
Anti-mouse CD80-Brilliant Violet 650 (https://www.biolegend.com/en-us/products/brilliant-violet-650-anti-mouse-cd80-antibody-7642)
Anti-mouse CD206-PE (https://www.biolegend.com/en-us/products/pe-anti-mouse-cd206-mmr-antibody-7424)
Anti-mouse F4/80-PE/Cyanine5 (https://www.biolegend.com/en-us/products/pe-cyanine5-anti-mouse-f4-80-antibody-4069)
Anti-mouse MHC class II-Brilliant Violet 605 (https://www.biolegend.com/en-us/products/brilliant-violet-605-anti-mouse-i-a-i-e-antibody-11988)
Anti-mouse CD44-Alexa Fluor® 647 (https://www.biolegend.com/en-us/products/alexa-fluor-647-anti-mouse-human-cd44-antibody-3098)
Anti-mouse NKp46-Brilliant Violet 510 (https://www.biolegend.com/en-us/products/brilliant-violet-510-anti-mouse-cd335-nkp46-antibody-9578)
Anti-mouse CD8a-Brilliant Violet 421 (https://www.biolegend.com/en-us/products/brilliant-violet-421-anti-mouse-cd8a-recombinant-antibody-18186)
Anti-mouse CD45RB-PerCP/Cyanine5.5 (https://www.biolegend.com/en-us/products/percp-cyanine5-5-anti-mouse-cd45rb-antibody-6225)
Anti-mouse CD45R/B220-Alexa Fluor® 700 (https://www.biolegend.com/en-us/products/alexa-fluor-700-anti-mouse-human-cd45r-b220-antibody-3408)
Anti-mouse Gr-1-APC/Fire™ 750 (https://www.biolegend.com/en-us/products/apc-fire-750-anti-mouse-ly-6g-ly-6c-gr-1-antibody-13202)
Anti-mouse CD16/32- (https://www.biolegend.com/en-us/products/trustain-fcx-anti-mouse-cd16-32-antibody-5683)

Anti-Mouse cGAS (https://www.cellsignal.com/products/primary-antibodies/cgas-d3o8o-rabbit-mab/31659)
Anti-b-actin (https://www.abcam.com/products/primary-antibodies/beta-actin-antibody-ac-15-ab6276.html)
Anti-STING (https://www.cellsignal.com/products/primary-antibodies/sting-d2p2f-rabbit-mab/13647)
Anti-STING (https://www.cellsignal.com/products/primary-antibodies/sting-d1v5l-rabbit-mab/50494)
Anti-α-tubulin (https://www.sigmaaldrich.com/US/en/product/sigma/t9026)
Anti-p-PERK (Thr980) (https://www.cellsignal.com/products/primary-antibodies/sting-d1v5l-rabbit-mab/50494)
Anti-PERK (https://www.cellsignal.com/products/primary-antibodies/perk-c33e10-rabbit-mab/3192)
Anti-BiP (https://www.cellsignal.com/products/primary-antibodies/bip-c50b12-rabbit-mab/3177)
Anti-CHOP (https://www.cellsignal.com/products/primary-antibodies/chop-l63f7-mouse-mab/2895)
Anti-ATF4 (https://www.cellsignal.com/products/primary-antibodies/atf-4-d4b8-rabbit-mab/11815)
Anti-p-eIF2α (Ser51) (https://www.cellsignal.com/products/primary-antibodies/phospho-eif2a-ser51-119a11-rabbit-mab/3597)
Anti-eIF2α (https://www.cellsignal.com/products/primary-antibodies/eif2a-d7d3-xp-rabbit-mab/5324)
Anti-CoxIV (https://www.abcam.com/products/primary-antibodies/cox-iv-antibody-mitochondrial-loading-control-ab16056.html)
Anti-β-Tubulin (https://www.thermofisher.com/antibody/product/beta-Tubulin-Antibody-clone-2-28-33-Monoclonal/32-2600)

Anti-Human cGAS (https://www.sigmaaldrich.com/US/en/product/mm/abf124)
Anti-Human cGAS (https://www.sigmaaldrich.com/US/en/product/sigma/hpa031700)
Anti-Human cGAS (https://www.lsbio.com/antibodies/c6orf150-antibody-mb21d1-antibody-clone-1697ct136.65.30-elisa-wb-western-ls-c757990/783701)
Anti-Human centromere proteins (https://www.antibodiesinc.com/products/anti-centromere-protein-antibody-15-234)

For human cGAS antibody in immunofluorescence staining, we validated them with human cell lines with Cgas shRNA knockdown samples.

Eukaryotic cell lines

Policy information about cell lines and Sex and Gender in Research

Cell line source(s)

4T1 (CRL-2539), B16F10 (CRL-6475), THP-1 (TIB-202), B16F0(CRL-6322), B16F1 (CRL-6323), IMR90 (CCL-186), EO771.lmb (CRL-3405), RAW 264.7 (TIB-71), and CT26 (CRL-2638) cells were purchased from American Type Culture Collection (ATCC).

Authentication

All cell lines used in this manuscript were authenticated by ATCC which used morphology, karyotyping and PCR-based techniques.

Mycoplasma contamination

All cell lines tested negative for mycoplasma using Lonza MycoAlert® Detection Kit.

Commonly misidentified lines (See ICLAC register)	None.

Animals and other research organisms

Policy information about studies involving animals; ARRIVE guidelines recommended for reporting animal research, and Sex and Gender in Research

Laboratory animals	All mice were purchased from Jackson Laboratories. 6-8-week-old female were used.<br>NOD-scid IL2Rgammanull (NSG) (Jackson Laboratories strain 005557)<br>Athymic nude (Jackson Laboratories strain 002019)<br>BALB/cJ (Jackson Laboratories strain 000651)<br>C57BL/6 (Jackson Laboratories strain 000664)<br><br>Animals were housed under the following conditions: 12-12 hour light-dark cycle, 21.1-22.2 °C, 30%-70% humidity.
Wild animals	No wild animals were used in the study.
Reporting on sex	All mice are female. EO771.lmb and 4T1 are breast cancer model. For B16F10 and CT26, female are widely used in literatures.
Field-collected samples	No field-collected samples were used in this study.
Ethics oversight	Animal experiments were performed in accordance with protocols approved by the MSKCC Institutional Animal Care and Use Committee.

Note that full information on the approval of the study protocol must also be provided in the manuscript.

Clinical data

Policy information about clinical studies
All manuscripts should comply with the ICMJE guidelines for publication of clinical research and a completed CONSORT checklist must be included with all submissions.

Clinical trial registration	N/A Retrospective Biospecimen protocol
Study protocol	MSKCC Institutional Review Board
Data collection	N/A
Outcomes	N/A

Flow Cytometry

Plots

Confirm that:

☒ The axis labels state the marker and fluorochrome used (e.g. CD4-FITC).

☒ The axis scales are clearly visible. Include numbers along axes only for bottom left plot of group (a 'group' is an analysis of identical markers).

☒ All plots are contour plots with outliers or pseudocolor plots.

☒ A numerical value for number of cells or percentage (with statistics) is provided.

Methodology

Sample preparation	Tumor pieces were digested to single-cell suspension with Colla- genase/Hyaluronidase (Stemcell Technologies, Catalog # 07912) and DNAase I (Stemcell Technologies, Catalog # 100-0762) according to the manufactural manual, followed by filtered with 70-µM cell strainers. Cells were stained with Zombie NIRTM Fixable Viability Kit (BioLegend Catalog # 423105) for 10 minutes on ice and followed by blocking with TruStain FcXTM (anti- mouse CD16/32) Antibody (BioLegend Catalog # 101319). Cells were then stained with fluorophore-conjugated antibody solution in PBS containing 2% FBS on ice for 30 minutes.
Instrument	Analysis was performed on a Cytek Aurora instrument.
Software	Data was analyzed with FlowJo software, version 10.8.2.
Cell population abundance	We didn't have any data using FACS sorted cells.

Gating strategy

Gating Strategy was illustrated in Supplementary Information.

☒ Tick this box to confirm that a figure exemplifying the gating strategy is provided in the Supplementary Information.
